# Supplementary material for: Effects of nutritional interventions on nutritional and immunological status and adherence to antiretroviral treatment among adults living with HIV in low- and middle-income countries: Systematic review and meta-analysis
Source: PLoS One. 2025 Jun 3;20(6):e0319843. doi: 10.1371/journal.pone.0319843 (PMC12132990; doi:10.1371/journal.pone.0319843)
Supplement: S1 Table — (DOCX) [file pone.0319843.s002.docx]

| **S1 Table.** Medline search strategy for the effects of nutritional interventions on nutritional status and health of people living with HIV/AIDS. *(Research question: In adults living with HIV/AIDS, in low- and middle-income countries(P), how nutritional and medical care (I) compared to medical care only (C), could improve nutritional status, adherence and response to antiretroviral therapy (ART) (O).* | | |
| --- | --- | --- |
| **NAME OF DATABASE (interface): MEDLINE (via the PubMed interface)** | | |
| **Concept** | **Line number** | **Search strategy** |
| **Concept 1**:  Acquired immunodeficiency syndrome | acquired immunodeficiency syndrome | "acquired immunodeficiency syndrome"[MeSH Terms] OR ("acquired"[TIAB] AND "immunodeficiency"[TIAB] AND "syndrome*"[TIAB]) OR "AIDS"[TIAB] NOT ("audiovisual aids"[MeSH Terms] OR ("audiovisual"[TIAB] AND "aids"[TIAB]) OR ("visual"[TIAB] AND "aids"[TIAB])) |
|  | HIV Infections | "HIV Infections"[Mesh:NoExp] OR "hiv"[MeSH Terms] OR "hiv"[TIAB] OR ("human"[TIAB] AND "immunodeficiency"[TIAB] AND "virus"[TIAB]) |
|  | Acute Retroviral Syndrome | "acute retroviral syndrome"[MeSH Terms] OR ("acute"[TIAB] AND "retroviral"[TIAB] AND "syndrome"[TIAB]) |
| **Concept 2:**  Nutritional intervention | Diet therapy | "diet therapy"[MeSH Subheading] OR ("diet*"[TIAB] AND "therapy"[TIAB]) OR "diet therapy"[MeSH Terms] OR ("diet*"[TIAB] AND "intervention"[TIAB]) OR ("diet*"[TIAB] AND "treatment"[TIAB]) |
|  | Food basket | ("food"[MeSH Terms] OR "food"[TIAB]) AND "basket*"[TIAB] |
|  | Food supplements | (("food"[MeSH Terms] OR "food"[TIAB] OR "diet"[MeSH Terms] OR "diet*"[TIAB]) AND "supplement*"[TIAB]) OR "dietary supplements"[MeSH Terms] |
|  | Micronutrient supplementation | ("micronutrients"[MeSH Terms] OR "micronutrient*"[TIAB] OR "micronutriment*"[TIAB] OR "trace elements"[MeSH Terms] OR ("trace"[TIAB] AND "element*"[TIAB])) AND ("supplement*"[TIAB] OR "powder*"[TIAB] OR "powders"[MeSH Terms]) |
|  | Multiple micronutrient | "multiple*"[TIAB] AND ("micronutrients"[MeSH Terms] OR "micronutrient*"[TIAB] OR "micronutriment*"[TIAB] OR "trace elements"[MeSH Terms] OR ("trace"[TIAB] AND "elements"[TIAB])) |
|  | Macronutrient supplementation | ("nutrients"[MeSH Terms] OR "nutrient*"[TIAB] OR "macronutrient*"[TIAB]) AND ("supplement*"[TIAB]) |
|  | Protein supplementation | ("protein*"[TIAB] OR "proteins"[MeSH Terms]) AND "supplement*"[TIAB] |
|  | High protein diet | "Diet, High-Protein"[Mesh:NoExp] OR ("diet"[TIAB] AND "high"[TIAB] AND "protein"[TIAB]) OR ("Protein-rich"[TIAB] AND ("food"[MeSH Terms] OR "food*"[TIAB])) |
|  | Amino acid supplementation | ("amino acids"[MeSH Terms] OR ("amino"[TIAB] AND "acid*"[TIAB])) AND ("supplement*"[TIAB]) |
|  | Legumes | "fabaceae"[MeSH Terms] OR "fabaceae"[TIAB] OR "legume*"[TIAB] |
|  | Spirulina | "spirulina"[MeSH Terms] OR "spirulina"[TIAB] |
|  | Alga supplementation | ("algal proteins"[MeSH Terms] OR "alga*"[TIAB]) AND "supplement*"[TIAB] |
|  | Animal source food | ("animals"[MeSH Terms:noexp] OR "animal"[TIAB]) AND ("source*"[TIAB]) AND ("food"[MeSH Terms] OR "food*"[TIAB]) |
|  | Meat consumption | ("meat"[MeSH Terms] OR "meat"[TIAB]) AND ("consumption*"[TIAB] OR "intake*"[TIAB]) |
|  | Fish intake | ("fishes"[MeSH Terms] OR "fish*"[TIAB]) AND ("consumption*"[TIAB] OR "intake*"[TIAB]) |
|  | Egg intake | ("ovum"[MeSH Terms] OR "ovum"[TIAB] OR "egg"[TIAB] OR "eggs"[TIAB]) AND ("consumption*"[TIAB] OR "intake*"[TIAB]) |
|  | Soya flour | ("soybeans"[MeSH Terms] OR "soybean*"[TIAB] OR "soya"[TIAB]) AND ("flour"[MeSH Terms] OR "flour*"[TIAB]) |
|  | Corn Soy blend | ("maize*"[TIAB] OR "zea mays"[MeSH Terms] OR ("zea"[TIAB] AND "mays"[TIAB]) OR "corn"[TIAB]) AND ("soybeans"[MeSH Terms] OR "soybean*"[TIAB] OR "soya"[TIAB]) AND "blend*"[TIAB] |
|  | Wheat soy blend | ("triticum"[MeSH Terms] OR "triticum"[TIAB] OR "wheat*"[TIAB]) AND ("soybeans"[MeSH Terms] OR "soybean*"[TIAB] OR "soya"[TIAB]) AND "blend*"[TIAB] |
|  | Nutritional rehabilitation | ("nutrition*"[TIAB] OR "nutritive"[TIAB]) AND ("rehabilit*"[TIAB] OR "rehabilitation"[MeSH Terms] OR "rehabilitation"[MeSH Subheading]) |
|  | Lipid-based nutrient supplements | ("Lipid-based"[TIAB] OR "Lipid based"[TIAB]) AND ("nutrient*"[TIAB] OR "nutrients"[MeSH Terms] OR "supplement*"[TIAB]) |
|  | Nutritional support | "nutritional support"[MeSH Terms] OR ("nutritional"[TIAB] AND "support"[TIAB]) OR "nutrition therapy"[MeSH Terms] OR ("nutrition"[TIAB] AND "therapy"[TIAB]) |
|  |  |  |
|  | Fortified Food | "food, fortified"[MeSH Terms] OR ("food*"[TIAB] AND "fortifi*"[TIAB]) |
| **Concept 3**:  Nutritional status | Nutritional status | "nutritional status"[MeSH Terms] OR ("nutrition*"[TIAB] AND "status"[TIAB]) |
|  | Body composition | "body composition"[MeSH Terms] OR ("body"[TIAB] AND "composition"[TIAB]) |
|  | Body Weight gain/Body weight loss/underweight/wasting/undernutrition | "weight gain"[MeSH Terms] OR "weight loss"[MeSH Terms] OR ("body"[TIAB] AND "weight"[TIAB] AND ("loss"[TIAB] OR "gain"[TIAB] OR "increase"[TIAB] OR "reduction"[TIAB] OR "loss"[TIAB] OR "decrease"[TIAB] OR "insufficiency"[TIAB])) OR "thinness"[MeSH Terms] OR "thinness"[TIAB] OR "underweight*"[TIAB] OR "cachexia"[MeSH Terms] OR "cachexia"[TIAB] OR "wasting"[TIAB] OR "undernutrition*"[TIAB] OR "wasting syndrome"[MeSH Terms] OR ("nutrition*"[TIAB] AND ("deficien*"[TIAB] OR "deficiency"[MeSH Subheading])) |
|  | Lean mass/ Fat free mass | ("lean"[TIAB] AND "mass"[TIAB]) OR ("fat"[TIAB] AND "free"[TIAB] AND "mass"[TIAB]) |
|  | Micronutrient deficiencies | ("micronutrients"[MeSH Terms] OR "micronutrient*"[TIAB] OR "micronutriment*"[TIAB] OR "trace elements"[MeSH Terms] OR ("trace"[TIAB] AND "element*"[TIAB])) AND ("deficien*"[TIAB] "deficiency"[MeSH Subheading]) |
|  | Anorexia | "anorexia"[MeSH Terms] OR "anorexi*"[TIAB] |
|  | Acute malnutrition | "acute*"[TIAB] AND ("malnutrition"[MeSH Terms] OR "malnutrition*"[TIAB] OR "malnourish*"[TIAB]) |
|  | Body mass index | "body mass index"[MeSH Terms] OR ("body"[TIAB] AND "mass"[TIAB] AND "index"[TIAB]) |
|  | Emaciation | "emaciation"[MeSH Terms] OR "emaciat*"[TIAB] |
| **Concept 4:**  Adherence and response to ART | HIV drug side effects | ("hiv"[MeSH Terms] OR "hiv"[TIAB]) AND ("drug related side effects and adverse reactions"[MeSH Terms] OR ("drug"[TIAB] AND "side"[TIAB] AND "effect*"[TIAB])) |
|  | Antiretroviral adherence | ("anti retroviral agents"[MeSH Terms] OR "anti retroviral"[TIAB] OR "antiretroviral*"[TIAB] OR "arv"[TIAB]) AND ("adherance"[TIAB] OR "adhere*"[TIAB]) |
|  | Viral load | "viral load"[MeSH Terms] OR ("viral"[TIAB] AND "load"[TIAB]) |
|  | CD3 and CD4 | "CD3"[TIAB] OR "CD4"[TIAB] |
| **Concept 5**:  Low and middle income countries  **#1b OR #2 OR #1** | #1b | afghanistan[Mesh:NoExp] OR albania[Mesh:NoExp] OR algeria[Mesh:NoExp] OR american samoa[Mesh:NoExp] OR angola[Mesh:NoExp] OR antigua and barbuda[Mesh:NoExp] OR argentina[Mesh:NoExp] OR armenia[Mesh:NoExp] OR aruba[Mesh:NoExp] OR azerbaijan[Mesh:NoExp] OR bahrain[Mesh:NoExp] OR bangladesh[Mesh:NoExp] OR barbados[Mesh:NoExp] OR republic of belarus[Mesh:NoExp] OR belize[Mesh:NoExp] OR benin[Mesh:NoExp] OR bhutan[Mesh:NoExp] OR bolivia[Mesh:NoExp] OR bosnia and herzegovina[Mesh:NoExp] OR botswana[Mesh:NoExp] OR brazil[Mesh:NoExp] OR bulgaria[Mesh:NoExp] OR burkina faso[Mesh:NoExp] OR burundi[Mesh:NoExp] OR cabo verde[Mesh:NoExp] OR cambodia[Mesh:NoExp] OR cameroon[Mesh:NoExp] OR central african republic[Mesh:NoExp] OR chad[Mesh:NoExp] OR chile[Mesh:NoExp] OR china[Mesh:NoExp] OR colombia[Mesh:NoExp] OR comoros[Mesh:NoExp] OR democratic republic of the congo[Mesh:NoExp] OR congo[Mesh:NoExp] OR costa rica[Mesh:NoExp] OR cote d’ivoire[Mesh:NoExp] OR croatia[Mesh:NoExp] OR cuba[Mesh:NoExp] OR cyprus[Mesh:NoExp] OR czech republic[Mesh:NoExp] OR djibouti[Mesh:NoExp] OR dominica[Mesh:NoExp] OR dominican republic[Mesh:NoExp] OR ecuador[Mesh:NoExp] OR egypt[Mesh:NoExp] OR el salvador[Mesh:NoExp] OR equatorial guinea[Mesh:NoExp] OR eritrea[Mesh:NoExp] OR estonia[Mesh:NoExp] OR swaziland[Mesh:NoExp] OR ethiopia[Mesh:NoExp] OR fiji[Mesh:NoExp] OR gabon[Mesh:NoExp] OR gambia[Mesh:NoExp] OR ghana[Mesh:NoExp] OR gibraltar[Mesh:NoExp] OR greece[Mesh:NoExp] OR grenada[Mesh:NoExp] OR guam[Mesh:NoExp] OR guatemala[Mesh:NoExp] OR guinea[Mesh:NoExp] OR guinea bissau[Mesh:NoExp] OR guyana[Mesh:NoExp] OR haiti[Mesh:NoExp] OR honduras[Mesh:NoExp] OR hungary[Mesh:NoExp] OR india[Mesh:NoExp] OR indonesia[Mesh:NoExp] OR iran[Mesh:NoExp] OR iraq[Mesh:NoExp] OR jamaica[Mesh:NoExp] OR jordan[Mesh:NoExp] OR kazakhstan[Mesh:NoExp] OR kenya[Mesh:NoExp] OR democratic people’s republic of korea[Mesh:NoExp] OR republic of korea[Mesh:NoExp] OR kosovo[Mesh:NoExp] OR kyrgyzstan[Mesh:NoExp] OR laos[Mesh:NoExp] OR latvia[Mesh:NoExp] OR lebanon[Mesh:NoExp] OR lesotho[Mesh:NoExp] OR liberia[Mesh:NoExp] OR libya[Mesh:NoExp] OR lithuania[Mesh:NoExp] OR macau[Mesh:NoExp] OR republic of north macedonia[Mesh:NoExp] OR madagascar[Mesh:NoExp] OR malawi[Mesh:NoExp] OR malaysia[Mesh:NoExp] OR indian ocean islands[Mesh:NoExp] OR mali[Mesh:NoExp] OR malta[Mesh:NoExp] OR micronesia[Mesh:NoExp] OR palau[Mesh:NoExp] OR mauritania[Mesh:NoExp] OR mauritius[Mesh:NoExp] OR mexico[Mesh:NoExp] OR moldova[Mesh:NoExp] OR mongolia[Mesh:NoExp] OR montenegro[Mesh:NoExp] OR morocco[Mesh:NoExp] OR mozambique[Mesh:NoExp] OR myanmar[Mesh:NoExp] OR namibia[Mesh:NoExp] OR nepal[Mesh:NoExp] OR netherlands antilles[Mesh:NoExp] OR nicaragua[Mesh:NoExp] OR niger[Mesh:NoExp] OR nigeria[Mesh:NoExp] OR oman[Mesh:NoExp] OR pakistan[Mesh:NoExp] OR panama[Mesh:NoExp] OR papua new guinea[Mesh:NoExp] OR paraguay[Mesh:NoExp] OR peru[Mesh:NoExp] OR philippines[Mesh:NoExp] OR poland[Mesh:NoExp] OR portugal[Mesh:NoExp] OR puerto rico[Mesh:NoExp] OR romania[Mesh:NoExp] OR russia[Mesh:NoExp] OR rwanda[Mesh:NoExp] OR samoa[Mesh:NoExp] OR sao tome and principe[Mesh:NoExp] OR saudi arabia[Mesh:NoExp] OR senegal[Mesh:NoExp] OR serbia[Mesh:NoExp] OR seychelles[Mesh:NoExp] OR sierra leone[Mesh:NoExp] OR slovakia[Mesh:NoExp] OR slovenia[Mesh:NoExp] OR melanesia[Mesh:NoExp] OR somalia[Mesh:NoExp] OR south africa[Mesh:NoExp] OR south sudan[Mesh:NoExp] OR sri lanka[Mesh:NoExp] OR saint kitts and nevis[Mesh:NoExp] OR saint lucia[Mesh:NoExp] OR saint vincent and the grenadines[Mesh:NoExp] OR sudan[Mesh:NoExp] OR suriname[Mesh:NoExp] OR syria[Mesh:NoExp] OR tajikistan[Mesh:NoExp] OR tanzania[Mesh:NoExp] OR thailand[Mesh:NoExp] OR timor leste[Mesh:NoExp] OR togo[Mesh:NoExp] OR tonga[Mesh:NoExp] OR trinidad and tobago[Mesh:NoExp] OR tunisia[Mesh:NoExp] OR turkey[Mesh:NoExp] OR turkmenistan[Mesh:NoExp] OR uganda[Mesh:NoExp] OR ukraine[Mesh:NoExp] OR uruguay[Mesh:NoExp] OR uzbekistan[Mesh:NoExp] OR vanuatu[Mesh:NoExp] OR venezuela[Mesh:NoExp] OR vietnam[Mesh:NoExp] OR middle east[Mesh:NoExp] OR yemen[Mesh:NoExp] OR yugoslavia[Mesh:NoExp] OR zambia[Mesh:NoExp] OR zimbabwe[Mesh:NoExp] OR africa south of the sahara[Mesh:NoExp] OR africa, central[Mesh:NoExp] OR africa, northern[Mesh:NoExp] OR africa, southern[Mesh:NoExp] OR africa, eastern[Mesh:NoExp] OR africa, western[Mesh:NoExp] OR west indies[Mesh:NoExp] OR indian ocean islands[Mesh:NoExp] OR caribbean region[Mesh:NoExp] OR central america[Mesh:NoExp] OR latin america[Mesh:NoExp] OR south america[Mesh:NoExp] OR asia, central[Mesh:NoExp] OR asia, northern[Mesh:NoExp] OR asia, southeastern[Mesh:NoExp] OR asia, western[Mesh:NoExp] OR europe, eastern[Mesh:NoExp] OR developing countries[Mesh:NoExp] |
|  | #2 | afghanistan[Text Word] OR albania[Text Word] OR algeria[Text Word] OR american samoa[Text Word] OR angola[Text Word] OR antigua[Text Word] OR barbuda[Text Word] OR argentina[Text Word] OR armenia[Text Word] OR armenian[Text Word] OR aruba[Text Word] OR azerbaijan[Text Word] OR bahrain[Text Word] OR bangladesh[Text Word] OR barbados[Text Word] OR belarus[Text Word] OR byelarus[Text Word] OR belorussia[Text Word] OR byelorussian[Text Word] OR belize[Text Word] OR british honduras[Text Word] OR benin[Text Word] OR dahomey[Text Word] OR bhutan[Text Word] OR bolivia[Text Word] OR bosnia[Text Word] OR herzegovina[Text Word] OR botswana[Text Word] OR bechuanaland[Text Word] OR brazil[Text Word] OR brasil[Text Word] OR bulgaria[Text Word] OR burkina faso[Text Word] OR burkina fasso[Text Word] OR upper volta[Text Word] OR burundi[Text Word] OR urundi[Text Word] OR cabo verde[Text Word] OR cape verde[Text Word] OR cambodia[Text Word] OR kampuchea[Text Word] OR khmer republic[Text Word] OR cameroon[Text Word] OR cameron[Text Word] OR cameroun[Text Word] OR central african republic[Text Word] OR ubangi shari[Text Word] OR chad[Text Word] OR chile[Text Word] OR china[Text Word] OR colombia[Text Word] OR comoros[Text Word] OR comoro islands[Text Word] OR mayotte[Text Word] OR congo[Text Word] OR zaire[Text Word] OR costa rica[Text Word] OR cote d’ivoire[Text Word] OR cote d’ ivoire[Text Word] OR cote divoire[Text Word] OR cote d ivoire[Text Word] OR ivory coast[Text Word] OR croatia[Text Word] OR cuba[Text Word] OR cyprus[Text Word] OR czech republic[Text Word] OR czechoslovakia[Text Word] OR djibouti[Text Word] OR french somaliland[Text Word] OR dominica[Text Word] OR dominican republic[Text Word] OR ecuador[Text Word] OR egypt[Text Word] OR united arab republic[Text Word] OR el salvador[Text Word] OR equatorial guinea[Text Word] OR spanish guinea[Text Word] OR eritrea[Text Word] OR estonia[Text Word] OR eswatini[Text Word] OR swaziland[Text Word] OR ethiopia[Text Word] OR fiji[Text Word] OR gabon[Text Word] OR gabonese republic[Text Word] OR gambia[Text Word] OR georgia[Text Word] OR georgian[Text Word] OR ghana[Text Word] OR gold coast[Text Word] OR gibraltar[Text Word] OR greece[Text Word] OR grenada[Text Word] OR guam[Text Word] OR guatemala[Text Word] OR guinea[Text Word] OR guyana[Text Word] OR guiana[Text Word] OR haiti[Text Word] OR hispaniola[Text Word] OR honduras[Text Word] OR hungary[Text Word] OR india[Text Word] OR indonesia[Text Word] OR timor[Text Word] OR iran[Text Word] OR iraq[Text Word] OR isle of man[Text Word] OR jamaica[Text Word] OR jordan[Text Word] OR kazakhstan[Text Word] OR kazakh[Text Word] OR kenya[Text Word] OR korea[Text Word] OR kosovo[Text Word] OR kyrgyzstan[Text Word] OR kirghizia[Text Word] OR kirgizstan[Text Word] OR kyrgyz republic[Text Word] OR kirghiz[Text Word] OR laos[Text Word] OR lao pdr[Text Word] OR lao people's democratic republic[Text Word] OR latvia[Text Word] OR lebanon[Text Word] OR lesotho[Text Word] OR basutoland[Text Word] OR liberia[Text Word] OR libya[Text Word] OR libyan arab jamahiriya[Text Word] OR lithuania[Text Word] OR macau[Text Word] OR macao[Text Word] OR macedonia[Text Word] OR madagascar[Text Word] OR malagasy republic[Text Word] OR malawi[Text Word] OR nyasaland[Text Word] OR malaysia[Text Word] OR maldives[Text Word] OR indian ocean[Text Word] OR mali[Text Word] OR malta[Text Word] OR micronesia[Text Word] OR kiribati[Text Word] OR marshall islands[Text Word] OR nauru[Text Word] OR northern mariana islands[Text Word] OR palau[Text Word] OR tuvalu[Text Word] OR mauritania[Text Word] OR mauritius[Text Word] OR mexico[Text Word] OR moldova[Text Word] OR moldovian[Text Word] OR mongolia[Text Word] OR montenegro[Text Word] OR morocco[Text Word] OR ifni[Text Word] OR mozambique[Text Word] OR portuguese east africa[Text Word] OR myanmar[Text Word] OR burma[Text Word] OR namibia[Text Word] OR nepal[Text Word] OR netherlands antilles[Text Word] OR nicaragua[Text Word] OR niger[Text Word] OR nigeria[Text Word] OR oman[Text Word] OR muscat[Text Word] OR pakistan[Text Word] OR panama[Text Word] OR papua new guinea[Text Word] OR paraguay[Text Word] OR peru[Text Word] OR philippines[Text Word] OR philipines[Text Word] OR phillipines[Text Word] OR phillippines[Text Word] OR poland[Text Word] OR polish people's republic[Text Word] OR portugal[Text Word] OR portuguese republic[Text Word] OR puerto rico[Text Word] OR romania[Text Word] OR russia[Text Word] OR russian federation[Text Word] OR ussr[Text Word] OR soviet union[Text Word] OR union of soviet socialist republics[Text Word] OR rwanda[Text Word] OR ruanda[Text Word] OR samoa[Text Word] OR pacific islands[Text Word] OR polynesia[Text Word] OR samoan islands[Text Word] OR sao tome and principe[Text Word] OR saudi arabia[Text Word] OR senegal[Text Word] OR serbia[Text Word] OR seychelles[Text Word] OR sierra leone[Text Word] OR slovakia[Text Word] OR slovak republic[Text Word] OR slovenia[Text Word] OR melanesia[Text Word] OR solomon island[Text Word] OR solomon islands[Text Word] OR norfolk island[Text Word] OR somalia[Text Word] OR south africa[Text Word] OR south sudan[Text Word] OR sri lanka[Text Word] OR ceylon[Text Word] OR saint kitts and nevis[Text Word] OR st kitts and nevis[Text Word] OR saint lucia[Text Word] OR st lucia[Text Word] OR saint vincent[Text Word] OR st vincent[Text Word] OR grenadines[Text Word] OR sudan[Text Word] OR suriname[Text Word] OR surinam[Text Word] OR syria[Text Word] OR syrian arab republic[Text Word] OR tajikistan[Text Word] OR tadjikistan[Text Word] OR tadzhikistan[Text Word] OR tadzhik[Text Word] OR tanzania[Text Word] OR tanganyika[Text Word] OR thailand[Text Word] OR siam[Text Word] OR timor leste[Text Word] OR east timor[Text Word] OR togo[Text Word] OR togolese republic[Text Word] OR tonga[Text Word] OR trinidad[Text Word] OR tobago[Text Word] OR tunisia[Text Word] OR turkey[Text Word] OR turkmenistan[Text Word] OR turkmen[Text Word] OR uganda[Text Word] OR ukraine[Text Word] OR uruguay[Text Word] OR uzbekistan[Text Word] OR uzbek[Text Word] OR vanuatu[Text Word] OR new hebrides[Text Word] OR venezuela[Text Word] OR vietnam[Text Word] OR viet nam[Text Word] OR middle east[Text Word] OR west bank[Text Word] OR gaza[Text Word] OR palestine[Text Word] OR yemen[Text Word] OR yugoslavia[Text Word] OR zambia[Text Word] OR zimbabwe[Text Word] OR northern rhodesia[Text Word] OR global south[Text Word] OR africa south of the sahara[Text Word] OR sub saharan africa[Text Word] OR subsaharan africa[Text Word] OR central africa[Text Word] OR north africa[Text Word] OR northern africa[Text Word] OR magreb[Text Word] OR maghrib[Text Word] OR sahara[Text Word] OR southern africa[Text Word] OR east africa[Text Word] OR eastern africa[Text Word] OR west africa[Text Word] OR western africa[Text Word] OR west indies[Text Word] OR indian ocean islands[Text Word] OR caribbean[Text Word] OR central america[Text Word] OR latin america[Text Word] OR south america[Text Word] OR central asia[Text Word] OR north asia[Text Word] OR northern asia[Text Word] OR southeastern asia[Text Word] OR south eastern asia[Text Word] OR southeast asia[Text Word] OR south east asia[Text Word] OR western asia[Text Word] OR east europe[Text Word] OR eastern europe[Text Word] OR least developed countries[Text Word] OR least developed country[Text Word] OR developing country[Text Word] OR developing countries[Text Word] OR developing nation[Text Word] OR developing nations[Text Word] OR developing population[Text Word] OR developing populations[Text Word] OR developing world[Text Word] OR less developed country[Text Word] OR less developed countries[Text Word] OR less developed nation[Text Word] OR less developed nations[Text Word] OR less developed world[Text Word] OR lesser developed countries[Text Word] OR lesser developed nations[Text Word] OR under developed country[Text Word] OR under developed countries[Text Word] OR under developed nations[Text Word] OR under developed world[Text Word] OR underdeveloped country[Text Word] OR underdeveloped countries[Text Word] OR underdeveloped nation[Text Word] OR underdeveloped nations[Text Word] OR underdeveloped population[Text Word] OR underdeveloped populations[Text Word] OR underdeveloped world[Text Word] OR middle income country[Text Word] OR middle income countries[Text Word] OR middle income nation[Text Word] OR middle income nations[Text Word] OR middle income population[Text Word] OR middle income populations[Text Word] OR low income country[Text Word] OR low income countries[Text Word] OR low income nation[Text Word] OR low income nations[Text Word] OR low income population[Text Word] OR low income populations[Text Word] OR lower income country[Text Word] OR lower income countries[Text Word] OR lower income nations[Text Word] OR lower income population[Text Word] OR lower income populations[Text Word] OR underserved countries[Text Word] OR underserved nations[Text Word] OR underserved population[Text Word] OR underserved populations[Text Word] OR under served population[Text Word] OR under served populations[Text Word] OR deprived countries[Text Word] OR deprived population[Text Word] OR deprived populations[Text Word] OR poor country[Text Word] OR poor countries[Text Word] OR poor nation[Text Word] OR poor nations[Text Word] OR poor population[Text Word] OR poor populations[Text Word] OR poor world[Text Word] OR poorer countries[Text Word] OR poorer nations[Text Word] OR poorer population[Text Word] OR poorer populations[Text Word] OR developing economy[Text Word] OR developing economies[Text Word] OR less developed economy[Text Word] OR less developed economies[Text Word] OR underdeveloped economies[Text Word] OR middle income economy[Text Word] OR middle income economies[Text Word] OR low income economy[Text Word] OR low income economies[Text Word] OR lower income economies[Text Word] OR low gdp[Text Word] OR low gnp[Text Word] OR low gross domestic[Text Word] OR low gross national[Text Word] OR lower gdp[Text Word] OR lower gross domestic[Text Word] OR "low and middle income country"[Text Word] OR "low and middle income countries"[Text Word] OR lmic[Text Word] OR lmics[Text Word] OR third world[Text Word] OR lami country[Text Word] OR lami countries[Text Word] OR transitional country[Text Word] OR transitional countries[Text Word] OR emerging economies[Text Word] OR emerging nation[Text Word] OR emerging nations[Text Word] |
|  | #1 | afghan[Text Word] OR afghans[Text Word] OR afghani[Text Word] OR albanian[Text Word] OR albanians[Text Word] OR algerian[Text Word] OR algerians[Text Word] OR american samoan[Text Word] OR american samoans[Text Word] OR angolan[Text Word] OR angolans[Text Word] OR antiguan[Text Word] OR antiguans[Text Word] OR barbudan[Text Word] OR argentine[Text Word] OR argentines[Text Word] OR argentinian[Text Word] OR argentinians[Text Word] OR argentinean[Text Word] OR argentineans[Text Word] OR armenian[Text Word] OR armenians[Text Word] OR aruban[Text Word] OR arubans[Text Word] OR azerbaijani[Text Word] OR azerbaijanis[Text Word] OR bahraini[Text Word] OR bahrainis[Text Word] OR bangladeshi[Text Word] OR bangladeshis[Text Word] OR bangalees[Text Word] OR bajan[Text Word] OR belarusian[Text Word] OR belarusians[Text Word] OR byelorussian[Text Word] OR byelorussians[Text Word] OR belizean[Text Word] OR belizeans[Text Word] OR beninese[Text Word] OR benineses[Text Word] OR bhutanese[Text Word] OR bolivian[Text Word] OR bolivians[Text Word] OR bosnian[Text Word] OR bosnians[Text Word] OR botswana[Text Word] OR batswana[Text Word] OR brazilian[Text Word] OR brazilians[Text Word] OR brasilian[Text Word] OR brasilians[Text Word] OR bulgarian[Text Word] OR bulgarians[Text Word] OR burkinabe[Text Word] OR burundian[Text Word] OR burundians[Text Word] OR cape verdean[Text Word] OR cape verdeans[Text Word] OR cabo verdean[Text Word] OR cabo verdeans[Text Word] OR cambodian[Text Word] OR cambodians[Text Word] OR khmer[Text Word] OR cameroonian[Text Word] OR cameroonians[Text Word] OR central african[Text Word] OR central africans[Text Word] OR chadian[Text Word] OR chadians[Text Word] OR chilean[Text Word] OR chileans[Text Word] OR chinese[Text Word] OR colombian[Text Word] OR colombians[Text Word] OR comorian[Text Word] OR comorians[Text Word] OR congolese[Text Word] OR costa rican[Text Word] OR costa ricans[Text Word] OR ivorian[Text Word] OR ivorians[Text Word] OR croatian[Text Word] OR croatians[Text Word] OR cuban[Text Word] OR cubans[Text Word] OR cypriot[Text Word] OR cypriots[Text Word] OR czech[Text Word] OR czechs[Text Word] OR djiboutian[Text Word] OR djiboutians[Text Word] OR dominican[Text Word] OR dominicans[Text Word] OR ecuadorian[Text Word] OR ecuadorians[Text Word] OR egyptian[Text Word] OR egyptians[Text Word] OR salvadoran[Text Word] OR salvadorans[Text Word] OR equatorial guinean[Text Word] OR equatorial guineans[Text Word] OR equatoguinean[Text Word] OR equatoguineans[Text Word] OR eritrean[Text Word] OR eritreans[Text Word] OR estonian[Text Word] OR estonians[Text Word] OR swazi[Text Word] OR swazis[Text Word] OR swati[Text Word] OR ethiopian[Text Word] OR ethiopians[Text Word] OR fijian[Text Word] OR fijians[Text Word] OR gabonese[Text Word] OR gambian[Text Word] OR gambians[Text Word] OR georgian[Text Word] OR georgians[Text Word] OR ghanaian[Text Word] OR ghanaians[Text Word] OR gibraltarian[Text Word] OR gibraltarians[Text Word] OR greek[Text Word] OR greeks[Text Word] OR grenadian[Text Word] OR grenadians[Text Word] OR guamanian[Text Word] OR guamanians[Text Word] OR guatemalan[Text Word] OR guatemalans[Text Word] OR guinean[Text Word] OR guineans[Text Word] OR bissau guinean[Text Word] OR bissau guineans[Text Word] OR guyanese[Text Word] OR haitian[Text Word] OR haitians[Text Word] OR honduran[Text Word] OR hondurans[Text Word] OR hungarian[Text Word] OR hungarians[Text Word] OR indian[Text Word] OR indians[Text Word] OR indonesian[Text Word] OR indonesians[Text Word] OR iranian[Text Word] OR iranians[Text Word] OR iraqian[Text Word] OR iraqi[Text Word] OR iraqis[Text Word] OR manx[Text Word] OR jamaican[Text Word] OR jamaicans[Text Word] OR jordanian[Text Word] OR jordanians[Text Word] OR kazakhstani[Text Word] OR kazakhstanis[Text Word] OR kenyan[Text Word] OR kenyans[Text Word] OR korean[Text Word] OR koreans[Text Word] OR kosovar[Text Word] OR kosovars[Text Word] OR kosovan[Text Word] OR kosovans[Text Word] OR kyrgyzstani[Text Word] OR kyrgyz[Text Word] OR lao[Text Word] OR laotian[Text Word] OR laotians[Text Word] OR latvian[Text Word] OR latvians[Text Word] OR lebanese[Text Word] OR mosotho[Text Word] OR basotho[Text Word] OR liberian[Text Word] OR liberians[Text Word] OR libyan[Text Word] OR libyans[Text Word] OR lithuanian[Text Word] OR lithuanians[Text Word] OR macanese[Text Word] OR macedonian[Text Word] OR macedonians[Text Word] OR malagasy[Text Word] OR madagascan[Text Word] OR madagascans[Text Word] OR malawian[Text Word] OR malawians[Text Word] OR malaysian[Text Word] OR malaysians[Text Word] OR maldivian[Text Word] OR maldivians[Text Word] OR malian[Text Word] OR malians[Text Word] OR maltese[Text Word] OR marshallese[Text Word] OR mauritanian[Text Word] OR mauritanians[Text Word] OR mauritian[Text Word] OR mauritians[Text Word] OR mexican[Text Word] OR mexicans[Text Word] OR micronesian[Text Word] OR micronesians[Text Word] OR moldovan[Text Word] OR moldovans[Text Word] OR mongolian[Text Word] OR mongolians[Text Word] OR mongol[Text Word] OR montenegrin[Text Word] OR montenegrins[Text Word] OR moroccan[Text Word] OR moroccans[Text Word] OR mozambican[Text Word] OR mozambicans[Text Word] OR burmese[Text Word] OR myanma[Text Word] OR namibian[Text Word] OR namibians[Text Word] OR nauruan[Text Word] OR nauruans[Text Word] OR nepali[Text Word] OR nepalese[Text Word] OR netherlands antillean[Text Word] OR netherlands antilleans[Text Word] OR nicaraguan[Text Word] OR nicaraguans[Text Word] OR nigerien[Text Word] OR nigeriens[Text Word] OR nigerian[Text Word] OR nigerians[Text Word] OR northern mariana islander[Text Word] OR northern mariana islanders[Text Word] OR mariana[Text Word] OR marianas[Text Word] OR omani[Text Word] OR omanis[Text Word] OR pakistani[Text Word] OR pakistanis[Text Word] OR palauan[Text Word] OR palauans[Text Word] OR panamanian[Text Word] OR panamanians[Text Word] OR papua new guinean[Text Word] OR papua new guineans[Text Word] OR paraguayan[Text Word] OR paraguayans[Text Word] OR peruvian[Text Word] OR peruvians[Text Word] OR philippine[Text Word] OR philippines[Text Word] OR philipine[Text Word] OR philipines[Text Word] OR phillipine[Text Word] OR phillipines[Text Word] OR phillippine[Text Word] OR phillippines[Text Word] OR filipino[Text Word] OR filipinos[Text Word] OR filipina[Text Word] OR filipinas[Text Word] OR polish[Text Word] OR pole[Text Word] OR poles[Text Word] OR portuguese[Text Word] OR puerto rican[Text Word] OR puerto ricans[Text Word] OR romanian[Text Word] OR romanians[Text Word] OR russian[Text Word] OR russians[Text Word] OR soviet people[Text Word] OR soviet population[Text Word] OR rwandan[Text Word] OR rwandans[Text Word] OR rwandese[Text Word] OR ruandans[Text Word] OR ruandese[Text Word] OR samoan[Text Word] OR samoans[Text Word] OR sao tomean[Text Word] OR sao tomeans[Text Word] OR saudi arabian[Text Word] OR saudi arabians[Text Word] OR saudi[Text Word] OR saudis[Text Word] OR senegalese[Text Word] OR serbian[Text Word] OR serbians[Text Word] OR montenegrin[Text Word] OR montenegrins[Text Word] OR seychellois[Text Word] OR seychelloise[Text Word] OR sierra leonean[Text Word] OR sierra leoneans[Text Word] OR slovak[Text Word] OR slovaks[Text Word] OR slovene[Text Word] OR slovenes[Text Word] OR solomon islander[Text Word] OR solomon islanders[Text Word] OR somali[Text Word] OR somalis[Text Word] OR south african[Text Word] OR south africans[Text Word] OR south sudanese[Text Word] OR sri lankan[Text Word] OR sri lankans[Text Word] OR ceylonese[Text Word] OR kittitian[Text Word] OR kittitians[Text Word] OR saint lucian[Text Word] OR saint lucians[Text Word] OR vincentian[Text Word] OR vincentians[Text Word] OR sudanese[Text Word] OR surinamese[Text Word] OR syrian[Text Word] OR syrians[Text Word] OR tajik[Text Word] OR tajiks[Text Word] OR tajikistani[Text Word] OR tanzanian[Text Word] OR tanzanians[Text Word] OR tanganyikan[Text Word] OR thai[Text Word] OR timorese[Text Word] OR timoreses[Text Word] OR togolese[Text Word] OR tongan[Text Word] OR tongans[Text Word] OR trinidadian[Text Word] OR trinidadians[Text Word] OR tobagonian[Text Word] OR tobagonians[Text Word] OR tunisian[Text Word] OR tunisians[Text Word] OR turk[Text Word] OR turks[Text Word] OR turkish[Text Word] OR turkmen[Text Word] OR turkmens[Text Word] OR tuvaluan[Text Word] OR tuvaluans[Text Word] OR ugandan[Text Word] OR ugandans[Text Word] OR ukrainian[Text Word] OR ukrainians[Text Word] OR uruguayan[Text Word] OR uruguayans[Text Word] OR uzbek[Text Word] OR uzbeks[Text Word] OR vanuatu[Text Word] OR vanuatuan[Text Word] OR vanuatuans[Text Word] OR venezuelan[Text Word] OR venezuelans[Text Word] OR vietnamese[Text Word] OR yemeni[Text Word] OR yemenis[Text Word] OR yemenite[Text Word] OR yemenites[Text Word] OR yemenese[Text Word] OR yugoslav[Text Word] OR yugoslavs[Text Word] OR yugoslavian[Text Word] OR yugoslavians[Text Word] OR zambian[Text Word] OR zambians[Text Word] OR zimbabwean[Text Word] OR zimbabweans[Text Word] |
| **Concept 6**: study design | 6 | "randomized controlled trial"[pt] OR "controlled clinical trial"[pt] OR "randomized"[TIAB] OR placebo[tiab] OR "drug therapy"[SH]OR "randomly"[tiab] OR "trial"[tiab] OR "groups"[tiab] NOT ("animals"[mh] NOT "humans"[mh]) |
