# Supplementary material for: Effects of nutritional interventions on nutritional and immunological status and adherence to antiretroviral treatment among adults living with HIV in low- and middle-income countries: Systematic review and meta-analysis
Source: PLoS One. 2025 Jun 3;20(6):e0319843. doi: 10.1371/journal.pone.0319843 (PMC12132990; doi:10.1371/journal.pone.0319843)
Supplement: S2 Table — (DOCX) [file pone.0319843.s003.docx]

| **S2 Table.** Embase search strategy for the effects of nutritional interventions on nutritional status and health of people living with HIV/AIDS. *(Research question: In adults living with HIV/AIDS, in low- and middle-income countries(P), how nutritional and medical care (I) compared to medical care only (C), could improve nutritional status, adherence and response to antiretroviral therapy (ART) (O).* | | |
| --- | --- | --- |
| **NAME OF DATABASE (interface): Embase (via the embase.com)** | | |
| **Concept** | **Line number** | **Search strategy** |
| **Concept 1**:  Acquired immunodeficiency syndrome | acquired immunodeficiency syndrome | 'acquired immune deficiency syndrome'/exp OR (acquired:ta,ab,kw AND ((('immune'/exp OR immune:ta,ab,kw) AND ('deficiency'/exp OR deficiency:ta,ab,kw)) OR 'immunodeficiency'/exp OR immunodeficiency:ta,ab,kw) AND ('syndrome'/exp OR syndrome:ta,ab,kw)) OR (aids:ta,ab,kw NOT ('audiovisual aids'/exp OR (audiovisual:ta,ab,kw AND ('aids'/exp OR aids:ta,ab,kw)))) |
|  | HIV Infections | 'human immunodeficiency virus infection'/exp OR (('human'/exp OR human:ta,ab,kw) AND ('immunodeficiency'/exp OR immunodeficiency:ta,ab,kw) AND ('virus'/exp OR virus:ta,ab,kw)) OR hiv:ta,ab,kw |
|  | Acute Retroviral Syndrome | 'acute hiv infection'/exp OR (acute:ta,ab,kw AND retroviral:ta,ab,kw AND ('syndrome'/exp OR syndrome:ta,ab,kw)) |
| **Concept 2:**  Nutritional intervention | Diet therapy | 'diet therapy'/exp OR (('diet'/exp OR diet*:ta,ab,kw) AND ('therapy'/exp OR therapy:ta,ab,kw OR 'intervention'/exp OR intervention:ta,ab,kw OR 'treatment'/exp OR treatment:ta,ab,kw)) OR 'diet intervention'/exp OR 'diet treatment'/exp OR 'dietary intervention'/exp OR 'dietary therapy'/exp OR 'dietary treatment'/exp |
|  | Food basket | ('food'/exp OR food*:ta,ab,kw) AND basket*:ta,ab,kw |
|  | Food supplement | ('food'/exp OR food*:ta,ab,kw) AND ('supplementation'/exp OR supplement*:ta,ab,kw) |
|  | Micronutrient supplementation | ('micronutrient'/exp OR micronutrient*:ta,ab,kw OR micronutriment*:ta,ab,kw OR 'trace element':ta,ab,kw) AND ('supplementation'/exp OR supplement*:ta,ab,kw OR 'powder'/exp OR powder*:ta,ab,kw) |
|  | Multiple micronutrient | multiple:ta,ab,kw AND ('micronutrient'/exp OR micronutrient*:ta,ab,kw OR micronutriment*:ta,ab,kw OR 'trace element':ta,ab,kw) |
|  | Macronutrient supplementation | ('macronutrient'/exp OR macronutrient*:ta,ab,kw OR 'nutrient'/exp OR nutrient*:ta,ab,kw) AND ('supplementation'/exp OR supplement*:ta,ab,kw) |
|  | Protein supplementation | 'protein supplementation'/exp OR (('protein'/exp OR protein*:ta,ab,kw) AND ('supplementation'/exp OR supplement*:ta,ab,kw)) |
|  | High protein diet | 'high protein diet'/exp OR (high:ta,ab,kw AND ('protein'/exp OR protein:ta,ab,kw) AND ('diet'/exp OR diet:ta,ab,kw OR 'food'/exp OR food:ta,ab,kw)) |
|  | Amino acid supplementation | 'amino acid supplementation'/exp OR (amino:ta,ab,kw AND ('acid'/exp OR acid*:ta,ab,kw) AND ('supplementation'/exp OR supplement*:ta,ab,kw)) |
|  | Legumes | 'legume'/exp OR legume*:ta,ab,kw OR 'leguminous vegetables'/exp OR (leguminous:ta,ab,kw AND ('vegetables'/exp OR vegetable*:ta,ab,kw)) |
|  | Spirulina | 'spirulina'/exp OR spirulina:ta,ab,kw |
|  | Alga supplementation | ('alga'/exp OR alga*:ta,ab,kw) AND ('supplementation'/exp OR supplement*:ta,ab,kw) |
|  | Animal source food | ('animal'/exp OR animal*:ta,ab,kw) AND source:ta,ab,kw AND ('food'/exp OR food*:ta,ab,kw) |
|  | Meat consumption | 'meat consumption'/exp OR (('meat'/exp OR meat:ta,ab,kw) AND ('consumption'/exp OR consumption:ta,ab,kw OR intake:ta,ab,kw)) OR 'meat intake'/exp |
|  | Fish intake | 'fish consumption'/exp OR (('fish'/exp OR fish:ta,ab,kw) AND ('consumption'/exp OR consumption:ta,ab,kw OR intake:ta,ab,kw)) OR 'fish intake'/exp |
|  | Egg intake | ('egg'/exp OR egg:ta,ab,kw) AND ('consumption'/exp OR consumption:ta,ab,kw OR intake:ta,ab,kw) |
|  | Soya flour | 'soya flour'/exp OR (('soya'/exp OR soya:ta,ab,kw OR 'soybean'/exp OR soybean:ta,ab,kw) AND ('flour'/exp OR flour*:ta,ab,kw)) |
|  | Corn Soya blend | ('corn'/exp OR corn:ta,ab,kw) AND ('soya'/exp OR soya:ta,ab,kw OR 'soybean'/exp OR soybean:ta,ab,kw) AND blend:ta,ab,kw |
|  | Wheat soy blend | ('wheat'/exp OR wheat:ta,ab,kw) AND ('soya'/exp OR soya:ta,ab,kw OR 'soybean'/exp OR soybean:ta,ab,kw) AND blend:ta,ab,kw |
|  | Nutritional rehabilitation | 'nutritional rehabilitation'/exp OR (nutrition*:ta,ab,kw AND ('rehabilitation'/exp OR rehabilitat*:ta,ab,kw)) |
|  | Lipid-based nutrient supplements | lipid:ta,ab,kw AND ('nutrient'/exp OR nutrient*:ta,ab,kw) AND ('supplementation'/exp OR supplement*:ta,ab,kw) |
|  | Nutritional support | 'nutritional support'/exp OR (nutrition*:ta,ab,kw AND ('support'/exp OR support*:ta,ab,kw)) |
|  | Diet supplementation | 'diet supplementation'/exp OR (('diet'/exp OR diet*:ta,ab,kw) AND ('supplementation'/exp OR supplement*:ta,ab,kw)) OR 'nutritional supplementation'/exp OR (nutrition*:ta,ab,kw AND ('supplementation'/exp OR supplement*:ta,ab,kw)) |
|  | Fortified Food | 'fortified food'/exp OR (fortif*:ta,ab,kw AND ('food'/exp OR food*:ta,ab,kw)) |
| **Concept 3**:  Nutritional status | Nutritional status | 'nutritional status'/exp OR (nutrition*:ta,ab,kw AND status:ta,ab,kw) OR 'nutritional state'/exp OR (nutrition*:ta,ab,kw AND ('state'/exp OR state:ta,ab,kw)) |
|  | Body composition | 'body composition'/exp OR (('body'/exp OR body:ta,ab,kw) AND composition:ta,ab,kw) |
|  | Body Weight gain/ Body weight loss/underweight/wasting/undernutrition | 'body weight gain'/exp OR (('body'/exp OR body:ta,ab,kw) AND ('weight'/exp OR weight:ta,ab,kw) AND (gain:ta,ab,kw OR increase:ta,ab,kw OR 'reduction'/exp OR reduction:ta,ab,kw OR 'loss'/exp OR loss:ta,ab,kw OR decrease:ta,ab,kw OR insufficiency:ta,ab,kw)) OR 'body weight increase'/exp OR 'weight gain'/exp OR 'weight increase'/exp OR 'body weight loss'/exp OR 'body weight decrease'/exp OR 'body weight reduction'/exp OR 'underweight'/exp OR underweight:ta,ab,kw OR 'thinness'/exp OR thinness:ta,ab,kw OR 'weight insufficiency'/exp OR 'malnutrition'/exp OR malnutrition:ta,ab,kw OR 'deficient nutrition'/exp OR (deficient:ta,ab,kw AND ('nutrition'/exp OR nutrition:ta,ab,kw)) |
|  | Lean mass / Fat free mass | 'lean mass'/exp OR (lean:ta,ab,kw AND ('mass'/exp OR mass:ta,ab,kw)) OR 'fat free mass'/exp OR (('fat'/exp OR fat:ta,ab,kw) AND free:ta,ab,kw AND ('mass'/exp OR mass:ta,ab,kw)) |
|  | Micronutrient deficiencies | ('micronutrient'/exp OR micronutrient*:ta,ab,kw) AND deficien*:ta,ab,kw |
|  | Anorexia | 'anorexia'/exp OR anorexia:ta,ab,kw |
|  | Acute malnutrition | acute*:ta,ab,kw AND ('malnutrition'/exp OR malnutrition:ta,ab,kw OR malnourish*:ta,ab,kw) |
|  | Body mass index | 'body mass index'/exp OR (('body'/exp OR body:ta,ab,kw) AND ('mass'/exp OR mass:ta,ab,kw) AND ('index'/exp OR index:ta,ab,kw)) |
|  | Emaciation | 'emaciation'/exp OR emaciat*:ta,ab,kw |
|  | cachexia | 'cachexia'/exp OR cachexia:ta,ab,kw |
| **Concept 4:**  Adherence and response to ART | HIV drug side effects | ('hiv'/exp OR hiv:ta,ab,kw) AND ('drug'/exp OR drug:ta,ab,kw) AND side:ta,ab,kw AND effect*:ta,ab,kw |
|  | Antiretroviral adherence | (antiretroviral:ta,ab,kw OR 'anti retroviral':ta,ab,kw) AND ('adherence'/exp OR adherence:ta,ab,kw) |
|  | Viral load | 'viral load'/exp OR (viral:ta,ab,kw AND ('load'/exp OR load:ta,ab,kw)) |
|  | CD3 and CD4 | 'cd3'/exp OR cd3:ta,ab,kw OR 'cd4'/exp OR cd4:ta,ab,kw |
| **Concept 5**: Low and middle income countries |  | 'Afghanistan'/exp OR 'afghan*':ti,ab,kw OR 'Albania'/exp OR 'albania*':ti,ab,kw OR 'Algeria'/exp OR 'algeria*':ti,ab,kw OR 'American Samoa'/exp OR 'american samoa*':ti,ab,kw OR 'Angola'/exp OR 'angola*':ti,ab,kw OR 'Antigua and Barbuda'/exp OR 'antigua*':ti,ab,kw OR 'barbuda*':ti,ab,kw OR 'Argentina'/exp OR 'argentin*':ti,ab,kw OR 'Armenia'/exp OR 'armenia*':ti,ab,kw OR 'Aruba'/exp OR 'aruba*':ti,ab,kw OR 'Azerbaijan'/exp OR 'azerbaijan*':ti,ab,kw OR 'Bahrain'/exp OR 'bahrain*':ti,ab,kw OR 'Bangladesh'/exp OR 'bangladesh*':ti,ab,kw OR 'bangalees':ti,ab,kw OR 'Barbados'/exp OR 'barbados*':ti,ab,kw OR 'bajan*':ti,ab,kw OR 'Belarus'/exp OR 'belarus*':ti,ab,kw OR 'republic of belarus':ti,ab,kw OR 'byelarus*':ti,ab,kw OR 'belorussia*':ti,ab,kw OR 'byelorussian*':ti,ab,kw OR 'Belize'/exp OR 'belize*':ti,ab,kw OR 'british honduras*':ti,ab,kw OR 'Benin'/exp OR 'benin*':ti,ab,kw OR 'dahomey*':ti,ab,kw OR 'Bhutan'/exp OR 'bhutan*':ti,ab,kw OR 'Bolivia'/exp OR 'bolivia*':ti,ab,kw OR 'Bosnia and Herzegovina'/exp OR 'Bosnia and Herzegovina':ti,ab,kw OR 'bosnia*':ti,ab,kw OR 'herzegovina*':ti,ab,kw OR 'Botswana'/exp OR 'botswana*':ti,ab,kw OR 'batswana*':ti,ab,kw OR 'bechuanaland*':ti,ab,kw OR 'Brazil'/exp OR 'brazil*':ti,ab,kw OR 'brasil*':ti,ab,kw OR 'Bulgaria'/exp OR 'bulgaria*':ti,ab,kw OR 'Burkina Faso'/exp OR 'burkina fasso*':ti,ab,kw OR 'burkinabe*':ti,ab,kw OR 'burkinese*':ti,ab,kw OR 'upper volta*':ti,ab,kw OR 'Burundi'/exp OR 'burundi*':ti,ab,kw OR 'urundi*':ti,ab,kw OR 'cabo verde*':ti,ab,kw OR 'Cape Verde'/exp OR 'cape verde*':ti,ab,kw OR 'Cambodia'/exp OR 'cambodia*':ti,ab,kw OR 'kampuchea':ti,ab,kw OR 'khmer republic':ti,ab,kw OR 'khmer':ti,ab,kw OR 'Cameroon'/exp OR 'cameroon*':ti,ab,kw OR 'cameron':ti,ab,kw OR 'cameroun':ti,ab,kw OR 'Central African Republic'/exp OR 'central african republic':ti,ab,kw OR 'central african*':ti,ab,kw OR 'ubangi shari':ti,ab,kw OR 'Chad'/exp OR 'chad*':ti,ab,kw OR 'Chile'/exp OR 'chile':ti,ab,kw OR 'China'/exp OR 'china':ti,ab,kw OR 'chinese':ti,ab,kw OR 'Colombia'/exp OR 'colombia*':ti,ab,kw OR 'Comoros'/exp OR 'comoros':ti,ab,kw OR 'comoro islands':ti,ab,kw OR 'iles comores':ti,ab,kw OR 'comorian*':ti,ab,kw OR 'Mayotte'/exp OR 'mayotte':ti,ab,kw OR 'democratic republic of the congo':ti,ab,kw OR 'Democratic Republic Congo'/exp OR 'congo'/exp OR 'congo*':ti,ab,kw OR 'zaire':ti,ab,kw OR 'Costa Rica'/exp OR 'costa rica*':ti,ab,kw OR 'cote d` Ivoire'/exp OR 'cote d` Ivoire':ti,ab,kw OR 'cote d` Ivoire':ti,ab,kw OR 'cote divoire':ti,ab,kw OR 'cote d ivoire':ti,ab,kw OR 'ivory coast':ti,ab,kw OR 'ivorian*':ti,ab,kw OR 'Croatia'/exp OR 'crotia*':ti,ab,kw OR 'Cuba'/exp OR 'cuba*':ti,ab,kw OR 'Cyprus'/exp OR 'cyprus':ti,ab,kw OR 'cypriot*':ti,ab,kw OR 'Czech Republic'/exp OR 'czech*':ti,ab,kw OR 'Czechoslovakia'/exp OR 'czechoslovakia':ti,ab,kw OR 'Djibouti'/exp OR 'djibouti*':ti,ab,kw OR 'Somaliland'/exp OR 'french somaliland':ti,ab,kw OR 'Dominica'/exp OR 'dominica*':ti,ab,kw OR 'Dominican Republic'/exp OR 'Ecuador'/exp OR 'ecuador*':ti,ab,kw OR 'Egypt'/exp OR 'egypt*':ti,ab,kw OR 'united arab republic':ti,ab,kw OR 'El Salvador'/exp OR 'el salvador':ti,ab,kw OR 'salvadoran*':ti,ab,kw OR 'Equatorial Guinea'/exp OR 'equatorial guinea*':ti,ab,kw OR 'equatoguinean*':ti,ab,kw OR 'spanish guinea':ti,ab,kw OR 'Eritrea'/exp OR 'eritrea*':ti,ab,kw OR 'Estonia'/exp OR 'estonia*':ti,ab,kw OR 'Eswatini'/exp OR 'eswatini':ti,ab,kw OR 'swaziland':ti,ab,kw OR 'swazi*':ti,ab,kw OR 'swati*':ti,ab,kw OR 'Ethiopia'/exp OR 'ethiopia*':ti,ab,kw OR 'Fiji'/exp OR 'fiji*':ti,ab,kw OR 'Gabon'/exp OR 'gabon*':ti,ab,kw OR 'gabonese republic':ti,ab,kw OR 'Gambia'/exp OR 'gambia*':ti,ab,kw OR 'Georgia Republic'/exp OR 'Georgia'/exp OR 'georgia*':ti,ab,kw OR 'Ghana'/exp OR 'ghana*':ti,ab,kw OR 'gold coast':ti,ab,kw OR 'Gibraltar'/exp OR 'gibraltar*':ti,ab,kw OR 'Greece'/exp OR 'greece':ti,ab,kw OR 'greek*':ti,ab,kw OR 'Grenada'/exp OR 'grenada':ti,ab,kw OR 'grenadian*':ti,ab,kw OR 'Guam'/exp OR 'guam*':ti,ab,kw OR 'Guatemala'/exp OR 'guatemala*':ti,ab,kw OR 'Guinea'/exp OR 'guinea*':ti,ab,kw OR 'Guinea-Bissau'/exp OR 'Guinea-Bissau':ti,ab,kw OR 'guinea bissau':ti,ab,kw OR 'Guyana'/exp OR 'French Guyana'/exp OR 'British Guiana'/exp OR 'guyana':ti,ab,kw OR 'guyanese':ti,ab,kw OR 'Haiti'/exp OR 'haiti*':ti,ab,kw OR 'hispaniola':ti,ab,kw OR 'Honduras'/exp OR 'honduras':ti,ab,kw OR 'honduran*':ti,ab,kw OR 'Hungary'/exp OR 'hungary':ti,ab,kw OR 'hungarian*':ti,ab,kw OR 'India'/exp OR 'india*':ti,ab,kw OR 'Indonesia'/exp OR 'indonesia*':ti,ab,kw OR 'Timor-Leste'/exp OR 'timor':ti,ab,kw OR 'Iran'/exp OR 'iran*':ti,ab,kw OR 'Iraq'/exp OR 'iraq*':ti,ab,kw OR 'Isle of Man'/exp OR 'isle of man':ti,ab,kw OR 'manx':ti,ab,kw OR 'Jamaica'/exp OR 'jamaica*':ti,ab,kw OR 'Jordan'/exp OR 'jordan*':ti,ab,kw OR 'Kazakhstan'/exp OR 'kazakh*':ti,ab,kw OR 'Kenya'/exp OR 'kenya*':ti,ab,kw OR 'kirabati*':ti,ab,kw OR 'North Korea'/exp OR 'north korea*':ti,ab,kw OR 'democratic people` s republic of korea':ti,ab,kw OR 'republic of korea':ti,ab,kw OR 'South Korea'/exp OR 'south korea':ti,ab,kw OR 'korea*':ti,ab,kw OR 'Kosovo'/exp OR 'kosovo':ti,ab,kw OR 'kosovar*':ti,ab,kw OR 'kosovan*':ti,ab,kw OR 'Kyrgyzstan'/exp OR 'kyrgyzstan*':ti,ab,kw OR 'kirghizia':ti,ab,kw OR 'kirgizstan':ti,ab,kw OR 'kyrgyz republic':ti,ab,kw OR 'kirghiz':ti,ab,kw OR 'kyrgyz':ti,ab,kw OR 'Laos'/exp OR 'laos':ti,ab,kw OR 'lao':ti,ab,kw OR 'laotian*':ti,ab,kw OR 'lao pdr':ti,ab,kw OR 'lao people` s democratic republic':ti,ab,kw OR 'Latvia'/exp OR 'latvia*':ti,ab,kw OR 'Lebanon'/exp OR 'lebanon':ti,ab,kw OR 'lebanese republic':ti,ab,kw OR 'lebanese':ti,ab,kw OR 'Lesotho'/exp OR 'lesotho*':ti,ab,kw OR 'lesothan*':ti,ab,kw OR 'mosotho*':ti,ab,kw OR 'basutoland':ti,ab,kw OR 'basotho':ti,ab,kw OR 'Liberia'/exp OR 'liberia*':ti,ab,kw OR 'Libyan Arab Jamahiriya'/exp OR 'libya*':ti,ab,kw OR 'Lithuania'/exp OR 'lithuania*':ti,ab,kw OR 'macau*':ti,ab,kw OR 'Macao'/exp OR 'macao*':ti,ab,kw OR 'macanese':ti,ab,kw OR 'Republic of North Macedonia'/exp OR 'macedonia*':ti,ab,kw OR 'Madagascar'/exp OR 'madagascar':ti,ab,kw OR 'malagasy':ti,ab,kw OR 'madagascan*':ti,ab,kw OR 'Malawi'/exp OR 'malawi*':ti,ab,kw OR 'nyasaland':ti,ab,kw OR 'Malaysia'/exp OR 'malaysia*':ti,ab,kw OR 'malay federation':ti,ab,kw OR 'malaya federation':ti,ab,kw OR 'maldives':ti,ab,kw OR 'maldivian*':ti,ab,kw OR 'indian ocean islands':ti,ab,kw OR 'Indian Ocean'/exp OR 'indian ocean':ti,ab,kw OR 'Mali'/exp OR 'mali*':ti,ab,kw OR 'Malta'/exp OR 'malta*':ti,ab,kw OR 'maltese':ti,ab,kw OR 'micronesia*':ti,ab,kw OR 'Federated States of Micronesia'/exp OR 'Kiribati'/exp OR 'kiribati*':ti,ab,kw OR 'marshall islands'/exp OR 'marshall islands*':ti,ab,kw OR 'marshallese**':ti,ab,kw OR 'Nauru'/exp OR 'nauru*':ti,ab,kw OR 'Northern Mariana Islands'/exp OR 'northern mariana islands':ti,ab,kw OR 'palau'/exp OR 'palau':ti,ab,kw OR 'Tuvalu'/exp OR 'tuvalu':ti,ab,kw OR 'Mauritania'/exp OR 'mauritania*':ti,ab,kw OR 'Mauritius'/exp OR 'mauritius*':ti,ab,kw OR 'mauritian*':ti,ab,kw OR 'Mexico'/exp OR 'mexico':ti,ab,kw OR 'mexican*':ti,ab,kw OR 'Moldova'/exp OR 'moldova*':ti,ab,kw OR 'moldovian*':ti,ab,kw OR 'Mongolia'/exp OR 'mongolia*':ti,ab,kw OR 'mongol':ti,ab,kw OR 'montenegro republic'/exp OR 'Montenegro (republic)'/exp OR 'montenegro':ti,ab,kw OR 'montenegrin*':ti,ab,kw OR 'Morocco'/exp OR 'morocco':ti,ab,kw OR 'moroccan*':ti,ab,kw OR 'ifni':ti,ab,kw OR 'Mozambique'/exp OR 'mozambique':ti,ab,kw OR 'mozambican*':ti,ab,kw OR 'portuguese east africa':ti,ab,kw OR 'Myanmar'/exp OR 'myanma*':ti,ab,kw OR 'burma':ti,ab,kw OR 'burmese':ti,ab,kw OR 'Namibia'/exp OR 'namibia*':ti,ab,kw OR 'nauruan*':ti,ab,kw OR 'Nepal'/exp OR 'nepal*':ti,ab,kw OR 'Netherlands Antilles'/exp OR 'netherlands antille*':ti,ab,kw OR 'Nicaragua'/exp OR 'nicaragua*':ti,ab,kw OR 'Niger'/exp OR 'niger*':ti,ab,kw OR 'Nigeria'/exp OR 'nigeria*':ti,ab,kw OR 'northern mariana inslander*':ti,ab,kw OR 'mariana*':ti,ab,kw OR 'Oman'/exp OR 'oman*':ti,ab,kw OR 'muscat':ti,ab,kw OR 'Pakistan'/exp OR 'pakistan*':ti,ab,kw OR 'Panama'/exp OR 'panama*':ti,ab,kw OR 'Papua New Guinea'/exp OR 'papua new guinea*':ti,ab,kw OR 'new guinea*':ti,ab,kw OR 'palauan*':ti,ab,kw OR 'palnamian*':ti,ab,kw OR 'Paraguay'/exp OR 'paraguay*':ti,ab,kw OR 'Peru'/exp OR 'peru*':ti,ab,kw OR 'Philippines'/exp OR 'philippine*':ti,ab,kw OR 'philipine*':ti,ab,kw OR 'phillipine*':ti,ab,kw OR 'phillippine*':ti,ab,kw OR 'filipin*':ti,ab,kw OR 'Poland'/exp OR 'poland*':ti,ab,kw OR 'polish people` s republic':ti,ab,kw OR 'polish':ti,ab,kw OR 'pole*':ti,ab,kw OR 'Portugal'/exp OR 'portugal*':ti,ab,kw OR 'portuguese republic':ti,ab,kw OR 'portuguese':ti,ab,kw OR 'Puerto Rico'/exp OR 'puerto rico*':ti,ab,kw OR 'puerto rican*':ti,ab,kw OR 'Romania'/exp OR 'romania*':ti,ab,kw OR 'Russian Federation'/exp OR 'russia*':ti,ab,kw OR 'USSR'/exp OR 'ussr':ti,ab,kw OR 'soviet union':ti,ab,kw OR 'union of soviet socialist republics':ti,ab,kw OR 'soviet people':ti,ab,kw OR 'soviet population':ti,ab,kw OR 'Rwanda'/exp OR 'rwanda*':ti,ab,kw OR 'rwandese':ti,ab,kw OR 'ruanda*':ti,ab,kw OR 'ruandese':ti,ab,kw OR 'Samoa'/exp OR 'samoa*':ti,ab,kw OR 'Samoan Islands'/exp OR 'pacific islands':ti,ab,kw OR 'Polynesia'/exp OR 'polynesia*':ti,ab,kw OR 'navigator island*':ti,ab,kw OR 'Sao Tome and Principe'/exp OR 'sao tome and principe':ti,ab,kw OR 'sao tomean*':ti,ab,kw OR 'santomean*':ti,ab,kw OR 'Saudi Arabia'/exp OR 'saudi arabia*':ti,ab,kw OR 'saudi*':ti,ab,kw OR 'Senegal'/exp OR 'senegal*':ti,ab,kw OR 'Serbia'/exp OR 'serbia*':ti,ab,kw OR 'Seychelles'/exp OR 'seychell*':ti,ab,kw OR 'Sierra Leone'/exp OR 'sierra leone*':ti,ab,kw OR 'Slovakia'/exp OR 'slovakia*':ti,ab,kw OR 'slovak*':ti,ab,kw OR 'slovak republic':ti,ab,kw OR 'Slovenia'/exp OR 'slovenia*':ti,ab,kw OR 'slovene*':ti,ab,kw OR 'Melanesia'/exp OR 'melanesia*':ti,ab,kw OR 'Solomon Islands'/exp OR 'solomon island*':ti,ab,kw OR 'Norfolk Island'/exp OR 'norfolk island*':ti,ab,kw OR 'Somalia'/exp OR 'somali*':ti,ab,kw OR 'South Africa'/exp OR 'south africa*':ti,ab,kw OR 'South Sudan'/exp OR 'south sudan*':ti,ab,kw OR 'Sri Lanka'/exp OR 'sri lanka*':ti,ab,kw OR 'ceylon*':ti,ab,kw OR 'Saint Kitts and Nevis'/exp OR 'saint kitts and nevis':ti,ab,kw OR 'st. kitts and nevis':ti,ab,kw OR 'kittitian*':ti,ab,kw OR 'nevisian*':ti,ab,kw OR 'Saint Lucia'/exp OR 'saint lucia*':ti,ab,kw OR 'st. lucia':ti,ab,kw OR 'Saint Vincent and the Grenadines'/exp OR 'saint vincent and the grenadines':ti,ab,kw OR 'saint vincent':ti,ab,kw OR 'st. vincent':ti,ab,kw OR 'grenadines':ti,ab,kw OR 'vincentian*':ti,ab,kw OR 'Sudan'/exp OR 'sudan*':ti,ab,kw OR 'Suriname'/exp OR 'surinam*':ti,ab,kw OR 'Suriname'/exp OR 'surinam*':ti,ab,kw OR 'dutch guiana':ti,ab,kw OR 'netherlands guiana':ti,ab,kw OR 'Syrian Arab Republic'/exp OR 'syria*':ti,ab,kw OR 'Tajikistan'/exp OR 'tajik*':ti,ab,kw OR 'tadjikistan*':ti,ab,kw OR 'tadzhikistan':ti,ab,kw OR 'tadzhik':ti,ab,kw OR 'Tanzania'/exp OR 'tanzania*':ti,ab,kw OR 'tanganyika*':ti,ab,kw OR 'Thailand'/exp OR 'thailand':ti,ab,kw OR 'siam':ti,ab,kw OR 'Timor-Leste'/exp OR 'timor leste':ti,ab,kw OR 'timor-leste':ti,ab,kw OR 'timorese*':ti,ab,kw OR 'east timor':ti,ab,kw OR 'Togo'/exp OR 'togo*':ti,ab,kw OR 'togolese republic':ti,ab,kw OR 'Tonga'/exp OR 'tonga*':ti,ab,kw OR 'Trinidad and Tobago'/exp OR 'trinidad and tobago':ti,ab,kw OR 'trinidad*':ti,ab,kw OR 'tobago*':ti,ab,kw OR 'Tunisia'/exp OR 'tunisia*':ti,ab,kw OR 'Turkey Republic'/exp OR 'Turkey (Republic)'/exp OR 'turkey':ti,ab,kw OR 'turk*':ti,ab,kw OR 'Turkmenistan'/exp OR 'turkmenistan':ti,ab,kw OR 'turkmen*':ti,ab,kw OR 'tuvaluan*':ti,ab,kw OR 'Uganda'/exp OR 'uganda*':ti,ab,kw OR 'Ukraine'/exp OR 'ukrain*':ti,ab,kw OR 'Uruguay'/exp OR 'uruguay*':ti,ab,kw OR 'Uzbekistan'/exp OR 'uzbek*':ti,ab,kw OR 'Vanuatu'/exp OR 'vanuatu*':ti,ab,kw OR 'new hebrides':ti,ab,kw OR 'Venezuela'/exp OR 'venezuela*':ti,ab,kw OR 'Viet nam'/exp OR 'vietnam*':ti,ab,kw OR 'viet nam':ti,ab,kw OR 'Middle East'/exp OR 'middle east':ti,ab,kw OR 'west bank':ti,ab,kw OR 'Gaza Strip Palestine'/exp OR 'gaze':ti,ab,kw OR 'Palestine'/exp OR 'palestine':ti,ab,kw OR 'Yemen'/exp OR 'yemen*':ti,ab,kw OR 'Yugoslavia'/exp OR 'yugoslav*':ti,ab,kw OR 'Zambia'/exp OR 'zambia*':ti,ab,kw OR 'Zimbabwe'/exp OR 'zimbabwe*':ti,ab,kw OR 'northern rhodesia*':ti,ab,kw OR 'global south':ti,ab,kw OR 'Africa south of the Sahara'/exp OR 'africa south of the sahara':ti,ab,kw OR 'sub sahara africa':ti,ab,kw OR 'subsaharan africa':ti,ab,kw OR 'Central Africa'/exp OR 'central africa':ti,ab,kw OR 'africa, central':ti,ab,kw OR 'africa, northern':ti,ab,kw OR 'north africa':ti,ab,kw OR 'northern africa':ti,ab,kw OR 'magreb':ti,ab,kw OR 'maghrib':ti,ab,kw OR 'sahara':ti,ab,kw OR 'africa, southern':ti,ab,kw OR 'africa, southern':ti,ab,kw OR 'southern africa*':ti,ab,kw OR 'africa, eastern':ti,ab,kw OR 'east africa*':ti,ab,kw OR 'eastern africa*':ti,ab,kw OR 'africa, western':ti,ab,kw OR 'west africa*':ti,ab,kw OR 'western africa*':ti,ab,kw OR 'west indies':ti,ab,kw OR 'Indian Ocean'/exp OR 'indian ocean islands':ti,ab,kw OR 'Caribbean'/exp OR 'caribbean region':ti,ab,kw OR 'caribbean':ti,ab,kw OR 'caribbean islands':ti,ab,kw OR 'Central America'/exp OR 'central america*':ti,ab,kw OR 'South and Central America'/exp OR 'south and central america':ti,ab,kw OR 'latin america*':ti,ab,kw OR 'South America'/exp OR 'south america':ti,ab,kw OR 'Central Asia'/exp OR 'central asia*':ti,ab,kw OR 'Northern Asia'/exp OR 'northern asia*':ti,ab,kw OR 'north asia*':ti,ab,kw OR 'Southeast Asia'/exp OR 'southeastern asia*':ti,ab,kw OR 'south eastern asia*':ti,ab,kw OR 'southeast asia*':ti,ab,kw OR 'south east asia*':ti,ab,kw OR 'Western Asia'/exp OR 'western asia*':ti,ab,kw OR 'west asia*':ti,ab,kw OR 'Eastern Europe'/exp OR 'eastern europe*':ti,ab,kw OR 'east europe*':ti,ab,kw OR 'developing country'/exp OR 'developing countr*':ti,ab,kw OR 'developing nation*':ti,ab,kw OR 'developing population*':ti,ab,kw OR 'developing world':ti,ab,kw OR 'less developed countr*':ti,ab,kw OR 'less developed nation*':ti,ab,kw OR 'less developed population*':ti,ab,kw OR 'less developed world':ti,ab,kw OR 'lesser developed countr*':ti,ab,kw OR 'lesser developed nation*':ti,ab,kw OR 'lesser developed population*':ti,ab,kw OR 'lesser developed world':ti,ab,kw OR 'under developed countr*':ti,ab,kw OR 'under developed nation*':ti,ab,kw OR 'under developed population*':ti,ab,kw OR 'under developed world':ti,ab,kw OR 'underdeveloped countr*':ti,ab,kw OR 'underdeveloped nation*':ti,ab,kw OR 'underdeveloped population*':ti,ab,kw OR 'underdeveloped world':ti,ab,kw OR 'middle income countr*':ti,ab,kw OR 'middle income nation*':ti,ab,kw OR 'middle income population*':ti,ab,kw OR 'low income countr*':ti,ab,kw OR 'low income nation*':ti,ab,kw OR 'low income population*':ti,ab,kw OR 'lower income countr*':ti,ab,kw OR 'lower income nation*':ti,ab,kw OR 'lower income population*':ti,ab,kw OR 'underserved countr*':ti,ab,kw OR 'underserved nation*':ti,ab,kw OR 'underserved population*':ti,ab,kw OR 'underserved world':ti,ab,kw OR 'under served countr*':ti,ab,kw OR 'under served nation*':ti,ab,kw OR 'under served population*':ti,ab,kw OR 'under served world':ti,ab,kw OR 'deprived countr*':ti,ab,kw OR 'deprived nation*':ti,ab,kw OR 'deprived population*':ti,ab,kw OR 'deprived world':ti,ab,kw OR 'poor countr*':ti,ab,kw OR 'poor nation*':ti,ab,kw OR 'poor population*':ti,ab,kw OR 'poor world':ti,ab,kw OR 'poorer countr*':ti,ab,kw OR 'poorer nation*':ti,ab,kw OR 'poorer population*':ti,ab,kw OR 'poorer world':ti,ab,kw OR 'developing econom*':ti,ab,kw OR 'less developed econom*':ti,ab,kw OR 'lesser developed econom*':ti,ab,kw OR 'under developed econom*':ti,ab,kw OR 'underdeveloped econom*':ti,ab,kw OR 'middle income econom*':ti,ab,kw OR 'low income econom*':ti,ab,kw OR 'lower income econom*':ti,ab,kw OR 'low gdp':ti,ab,kw OR 'low gnp':ti,ab,kw OR 'low gross domestic':ti,ab,kw OR 'low gross national':ti,ab,kw OR 'lower gdp':ti,ab,kw OR 'lower gnp':ti,ab,kw OR 'lower gross domestic':ti,ab,kw OR 'lower gross national':ti,ab,kw OR 'lmic':ti,ab,kw OR 'lmics':ti,ab,kw OR 'third world':ti,ab,kw OR 'lami countr*':ti,ab,kw OR 'transitional countr*':ti,ab,kw OR 'emerging economies':ti,ab,kw OR 'emerging nation*':ti,ab,kw OR 'least developed countr*':ti,ab,kw OR 'low and middle income countr*':ti,ab,kw |
| **Concept 6**: study design | 6 | 'randomized controlled trial'/exp OR 'controlled clinical trial'/exp OR random*:ti,ab OR 'randomization'/exp OR 'intermethod comparison'/exp OR placebo:ti,ab OR compare:ti OR compared:ti OR comparison:ti OR ((evaluated:ab OR evaluate:ab OR evaluating:ab OR assessed:ab OR assess:ab) AND (compare:ab OR compared:ab OR comparing:ab OR comparison:ab)) OR ((open NEXT/1 label):ti,ab) OR (((double OR single OR doubly OR singly) NEXT/1 (blind OR blinded OR blindly)):ti,ab) OR 'double blind procedure'/exp OR 'parallel group*':ti,ab OR ((crossover:ti,ab OR cross:ti,ab) AND over:ti,ab) OR (((assign* OR match OR matched OR allocation) NEXT/5 (alternate OR group* OR intervention* OR patient* OR subject* OR participant*)):ti,ab) OR assigned:ti,ab OR allocated:ti,ab OR ((controlled NEXT/7 (study OR design OR trial)):ti,ab) OR volunteer:ti,ab OR volunteers:ti,ab OR 'human experiment'/exp OR trial:ti NOT ((((random* NEXT/1 sampl* NEXT/7 ("cross section*" OR questionnaire* OR survey* OR database*)):ti,ab) NOT (‘comparative study’/exp OR ‘controlled study’/exp OR ‘randomi?ed controlled’:ti,ab OR ‘randomly assigned’:ti,ab)) OR (‘Cross-sectional study’/exp NOT (‘randomized controlled trial’/exp OR ‘controlled clinical trial’/exp OR ‘controlled study’/exp OR ‘randomi?ed controlled’:ti,ab OR ‘control group*’:ti,ab)) OR ((((case NEXT/1 control*):ti,ab) AND random*:ti,ab) NOT 'randomi?ed controlled':ti,ab) OR ('systematic review':ti NOT (trial:ti OR study:ti)) OR **(nonrandom*:ti,ab NOT random*:ti,ab)** OR ‘Random field*’:ti,ab OR (random cluster NEXT/3 sampl*):ti,ab OR ((review:ab AND **review:pt**) NOT trial:ti) OR (‘we searched’:ab AND (review:ti OR review:pt)) OR ‘update review’:ab OR (‘databases NEXT/4 searched’:ab) OR ((rat OR rats OR mouse OR mice OR swine OR porcine OR murine OR sheep OR lambs OR pigs OR piglets OR rabbit OR rabbits OR cat OR cats OR dog OR dogs OR cattle OR bovine OR monkey OR monkeys OR trout OR marmoset*):ti AND ‘animal experiment’/exp) OR (‘Animal experiment’/exp NOT (‘human experiment’/exp OR human/exp))) |
