# Supplementary material for: Effects of nutritional interventions on nutritional and immunological status and adherence to antiretroviral treatment among adults living with HIV in low- and middle-income countries: Systematic review and meta-analysis
Source: PLoS One. 2025 Jun 3;20(6):e0319843. doi: 10.1371/journal.pone.0319843 (PMC12132990; doi:10.1371/journal.pone.0319843)
Supplement: S3 Table — (DOCX) [file pone.0319843.s004.docx]

| **S3 Table.** Cochrane search strategy for the effects of nutritional interventions on nutritional status and health of people living with HIV/AIDS. *(Research question: In adults living with HIV/AIDS, in low- and middle-income countries(P), how nutritional and medical care (I) compared to medical care only (C), could improve nutritional status, adherence and response to antiretroviral therapy (ART) (O).* | | |
| --- | --- | --- |
| **NAME OF DATABASE (interface):** Cochrane Library (via www.cochranelibrary.com) | | |
| **Concept** | **Line number** | **Search strategy** |
| **Concept 1**:  Acquired immunodeficiency syndrome | acquired immunodeficiency syndrome | [mh "acquired immunodeficiency syndrome"] OR ((acquired AND immunodeficiency AND syndrome*) OR AIDS):ti,ab,kw NOT ([mh "audiovisual aids"] OR ((audiovisual AND aids) OR (visual AND aids)):ti,ab,kw) |
|  | HIV Infections | [mh ^"HIV Infections"] OR [mh "hiv"] OR (hiv OR (human AND immunodeficiency AND virus)):ti,ab,kw |
|  | Acute Retroviral Syndrome | [mh "acute retroviral syndrome"] OR (acute AND retroviral AND syndrome):ti,ab,kw |
| **Concept 2:**  Nutritional intervention | Diet therapy | [mh "diet therapy"] OR ((diet* AND therapy) OR (diet* AND intervention) OR (diet* AND treatment)):ti,ab,kw |
|  | Food basket | ([mh "food"] OR food:ti,ab,kw) AND basket*:ti,ab,kw |
|  | Food supplements | (([mh "food"] OR [mh "diet"] OR food:ti,ab,kw OR diet*:ti,ab,kw) AND supplement*:ti,ab,kw) OR [mh "dietary supplements"] |
|  | Micronutrient supplementation | ([mh "micronutrients"] OR (micronutrient* OR micronutriment*):ti,ab,kw OR [mh "trace elements"] OR (trace AND element*):ti,ab,kw) AND ((supplement* OR powder*):ti,ab,kw OR [mh "powders"]) |
|  | Multiple micronutrient | ([mh "micronutrients"] OR (micronutrient* OR micronutriment*):ti,ab,kw OR [mh "trace elements"] OR (trace AND elements):ti,ab,kw) AND multiple*:ti,ab,kw |
|  | Macronutrient supplementation | ([mh "nutrients"] OR (nutrient* OR macronutrient*):ti,ab,kw) AND supplement*:ti,ab,kw |
|  | Protein supplementation | ([mh "proteins"] OR protein*:ti,ab,kw) AND supplement*:ti,ab,kw |
|  | High protein diet | (([mh "food"] OR (diet* OR food*):ti,ab,kw) AND ((high AND protein*):ti,ab,kw OR Protein-rich:ti,ab,kw)) OR [mh ^"Diet, High-Protein"] |
|  | Amino acid supplementation | ([mh "amino acids"] AND supplement*:ti,ab,kw) OR (amino AND acid* AND supplement*):ti,ab,kw |
|  | Legumes | [mh "fabaceae"] OR (fabaceae OR legume*):ti,ab,kw |
|  | Spirulina | [mh "spirulina"] OR spirulina:ti,ab,kw |
|  | Alga supplementation | [mh "algal proteins"] OR alga*:ti,ab,kw |
|  | Animal source food | ([mh ^"animals"] OR animal*:ti,ab,kw) AND ([mh "food"] OR food*:ti,ab,kw) |
|  | Meat consumption | ([mh "meat"] OR meat:ti,ab,kw) AND (consumption* OR intake*):ti,ab,kw |
|  | Fish intake | ([mh "fishes"] OR fish*:ti,ab,kw) AND (consumption* OR intake*):ti,ab,kw |
|  | Egg intake | ([mh "ovum"] OR (ovum OR egg OR eggs):ti,ab,kw) AND (consumption* OR intake*):ti,ab,kw |
|  | Soya flour | ([mh "soybeans"] OR (soybean* OR soya):ti,ab,kw) AND ([mh "flour"] OR flour*:ti,ab,kw) |
|  | Corn Soya blend | ([mh "zea mays"] OR ((zea AND mays) OR maize* OR corn):ti,ab,kw) AND ([mh "soybeans"] OR (soybean* OR soya):ti,ab,kw) AND blend*:ti,ab,kw |
|  | Wheat soya blend | ([mh "triticum"] OR (triticum OR wheat*):ti,ab,kw) AND ([mh "soybeans"] OR (soybean* OR soya):ti,ab,kw) AND blend*:ti,ab,kw |
|  | Nutritional rehabilitation | ([mh "rehabilitation"] OR [mh "rehabilitation"] OR rehabilit*:ti,ab,kw) AND (nutrition* OR nutritive):ti,ab,kw |
|  | Lipid-based nutrient supplements | (Lipid-based OR "Lipid based"):ti,ab,kw AND ([mh "nutrients"] OR nutrient*:ti,ab,kw) AND supplement*:ti,ab,kw |
|  | Nutritional support | [mh "nutritional support"] OR (nutrition* AND support):ti,ab,kw OR [mh "nutrition therapy"] OR (nutrition* AND therapy):ti,ab,kw |
|  | Fortified Food | [mh "food, fortified"] OR (food* AND fortifi*):ti,ab,kw |
| **Concept 3**:  Nutritional status | Nutritional status | [mh "nutritional status"] OR (nutrition* AND status):ti,ab,kw |
|  | Body composition | [mh "body composition"] OR (body AND composition):ti,ab,kw |
|  | Body Weight gain/Body weight loss/underweight/wasting/undernutrition | **[**mh **"**weight gain"] OR [mh "weight loss"] OR (body AND weight AND (loss OR gain OR increase OR reduction OR loss OR decrease OR insufficiency)):ti,ab,kw OR [mh "thinness"] OR [mh "cachexia"] OR [mh "wasting syndrome"] OR (thinness OR underweight* OR cachexia OR wasting OR undernutrition*):ti,ab,kw OR (nutrition*:ti,ab,kw AND ([mh "Deficiency Diseases"] OR deficien*:ti,ab,kw)) |
|  | Lean mass/ Fat free mass | ((lean AND mass) OR (fat AND free AND mass)):ti,ab,kw |
|  | Micronutrient deficiencies | ([mh "micronutrients"] OR [mh "trace elements"] OR (micronutrient* OR micronutriment* OR (trace AND element*)):ti,ab,kw) AND ([mh "Deficiency Diseases"] OR deficien*:ti,ab,kw) |
|  | Anorexia | [mh "anorexia"] OR anorexi*:ti,ab,kw |
|  | Acute malnutrition | ([mh "malnutrition"] OR (malnutrition* OR malnourish*):ti,ab,kw) AND acute*:ti,ab,kw |
|  | Body mass index | [mh "body mass index"] OR (body AND mass AND index):ti,ab,kw |
|  | Emaciation | [mh "emaciation"] OR emaciat*:ti,ab,kw |
| **Concept 4:**  Adherence and response to ART | HIV drug side effects | ([mh "hiv"] OR hiv:ti,ab,kw) AND ([mh "drug related side effects and adverse reactions"] OR (drug AND side AND effect*):ti,ab,kw) |
|  | Antiretroviral adherence | ([mh "anti retroviral agents"] OR ("anti retroviral" OR antiretroviral* OR arv):ti,ab,kw) AND (adherance OR adhere*):ti,ab,kw |
|  | Viral load | [mh "viral load"] OR (viral AND load):ti,ab,kw |
|  | CD3 and CD4 | (CD3 OR CD4):ti,ab,kw |
| **Concept 5**:  Low and middle income countries  **#1b OR #2 OR #1** | #1b | [mh ^“Afghanistan”] OR [mh ^“Albania”] OR [mh ^“Algeria”] OR [mh ^“american samoa”] OR [mh ^“angola”] OR [mh ^“antigua and barbuda”] OR [mh ^“argentina”] OR [mh ^“Armenia”] OR [mh ^“aruba”] OR [mh ^“Azerbaijan”] OR [mh ^“Bahrain”] OR [mh ^“Bangladesh”] OR [mh ^“Barbados”] OR [mh ^“republic of Belarus”] OR [mh ^“belize”] OR [mh ^“benin”] OR [mh ^“Bhutan”] OR [mh ^“Bolivia”] OR [mh ^“bosnia and Herzegovina”] OR [mh ^“Botswana”] OR [mh ^“brazil”] OR [mh ^“Bulgaria”] OR [mh ^“burkina faso”] OR [mh ^“burundi”] OR [mh ^“cabo verde”] OR [mh ^“Cambodia”] OR [mh ^“Cameroon”] OR [mh ^“central african republic”] OR [mh ^“chad”] OR [mh ^“chile”] OR [mh ^“china”] OR [mh ^“Colombia”] OR [mh ^“comoros”] OR [mh ^“democratic republic of the congo”] OR [mh ^“congo”] OR [mh ^“costa rica”] OR [mh ^“cote d’ivoire”] OR [mh ^“Croatia”] OR [mh ^“cuba”] OR [mh ^“cyprus”] OR [mh ^“czech republic”] OR [mh ^“Djibouti”] OR [mh ^“dominica”] OR [mh ^“dominican republic”] OR [mh ^“Ecuador”] OR [mh ^“Egypt”] OR [mh ^“el Salvador”] OR [mh ^“equatorial guinea”] OR [mh ^“Eritrea”] OR [mh ^“Estonia”] OR [mh ^“Swaziland”] OR [mh ^“Ethiopia”] OR [mh ^“fiji”] OR [mh ^“gabon”] OR [mh ^“gambia”] OR [mh ^“ghana”] OR [mh ^“Gibraltar”] OR [mh ^“Greece”] OR [mh ^“Grenada”] OR[mh ^“Guam”] OR [mh ^“Guatemala”] OR [mh ^“Guinea”] OR [mh ^“Guinea-Bissau”] OR [mh ^“Guyana”] OR [mh ^“French Guyana”] OR [mh ^“British Guiana”] OR [mh ^“Haiti”] OR [mh ^“Honduras”] OR [mh ^“Hungary”] OR [mh ^“India”] OR [mh ^“Indonesia”] OR [mh ^"Timor-Leste”] OR [mh ^“Iran”] OR [mh ^“Iraq”] OR [mh ^“Isle of Man”] OR [mh ^“Jamaica”] OR [mh ^“Jordan”] OR [mh ^“Kazakhstan”] OR [mh ^“Kenya"] OR [mh ^“North Korea”] OR [mh ^“South Korea”] OR [mh ^“Kosovo”] OR [mh ^“Kyrgyzstan”] OR [mh ^“Laos”] OR [mh ^“Latvia”] OR [mh ^“Lebanon”] OR [mh ^“Lesotho”] OR [mh ^“Liberia”] OR [mh ^“Libyan Arab Jamahiriya”] OR [mh ^“Lithuania”] OR [mh ^“Macao”] OR [mh ^“Republic of North Macedonia”] OR [mh ^“Madagascar”] OR [mh ^“Malawi”] OR [mh ^“Malaysia”] OR [mh ^“Indian Ocean”] OR [mh ^“Mali”] OR [mh ^“Malta”] OR [mh ^“Federated States of Micronesia”] OR [mh ^“Kiribati”] OR [mh ^“marshall islands”] OR [mh ^“Nauru”] OR [mh ^“Northern Mariana Islands”] OR [mh ^“palau”] OR [mh ^“Tuvalu”] OR [mh ^“Mauritania”] OR [mh ^“Mauritius”] OR [mh ^“Moldova”] OR [mh ^“Mexico”] OR [mh ^“Mongolia”] OR [mh ^“montenegro republic”] OR [mh ^“Montenegro (republic)”] OR [mh ^“Morocco”] OR [mh ^“Mozambique”] OR [mh ^“Myanmar”] OR [mh ^“Namibia”] OR [mh ^“Nepal”] OR [mh ^“Netherlands Antilles”] OR [mh ^“Nicaragua”] OR [mh ^“Niger”] OR [mh ^“Nigeria”] OR [mh ^“Oman”] OR [mh ^“Pakistan”] OR [mh ^“Panama”] OR [mh ^“Papua New Guinea”] OR [mh ^“Paraguay”] OR [mh ^“Peru”] OR [mh ^“Philippines”] OR [mh ^“Poland”] OR [mh ^“Portugal”] OR [mh ^“Puerto Rico”] OR [mh ^“Romania”] OR [mh ^“Russian Federation”] OR [mh ^“USSR”] OR [mh ^“Rwanda”] OR [mh ^“Samoa”] OR [mh ^“Samoan Islands”] OR [mh ^“Polynesia”] OR [mh ^“Sao Tome and Principe”] OR [mh ^“Saudi Arabia”] OR [mh ^“Senegal”] OR [mh ^“Serbia”] OR [mh ^“Seychelles”] OR [mh ^“Sierra Leone”] OR [mh ^“Slovakia”] OR [mh ^“Slovenia”] OR [mh ^“Melanesia”] OR [mh ^“Solomon Islands”] OR [mh ^“Norfolk Island”] OR [mh ^“Somalia”] OR [mh ^“South Africa”] OR [mh ^“South Sudan”] OR [mh ^“Sri Lanka”] OR [mh ^“Saint Kitts and Nevis”] OR [mh ^“Saint Lucia”] OR [mh ^“Saint Vincent and the Grenadines”] OR [mh ^“Sudan”] OR [mh ^“Suriname”] OR [mh ^“Suriname”] OR [mh ^“Syrian Arab Republic”] OR [mh ^“Tajikistan”] OR [mh ^“Tanzania”] OR [mh ^“Thailand”] OR [mh ^“Timor-Leste”] OR [mh ^“Togo”] OR [mh ^“Tonga”] OR [mh ^“Trinidad and Tobago”] OR [mh ^“Tunisia”] OR [mh ^“Turkey Republic”] OR [mh ^“Turkey (Republic)”] OR [mh ^“Turkmenistan”] OR [mh ^“Uganda”] OR [mh ^“Ukraine”] OR [mh ^“Uruguay”] OR [mh ^“Uzbekistan”] OR [mh ^“Vanuatu”] OR [mh ^“Venezuela”] OR [mh ^“Viet nam”] OR [mh ^“Middle East”] OR [mh ^“Gaza Strip Palestine”] OR [mh ^“Palestine”] OR [mh ^“Yemen”] OR [mh ^“Yugoslavia”] OR [mh ^“Zambia”] OR [mh ^“Zimbabwe”] OR [mh ^“Africa south of the Sahara”] OR [mh ^“Central Africa”] OR [mh ^“Indian Ocean”] OR [mh ^“Caribbean”] OR [mh ^“Central America”] OR [mh ^“South and Central America”] OR [mh ^“South America”] OR [mh ^“Central Asia”] OR [mh ^“Northern Asia”] OR [mh ^“Southeast Asia”] OR [mh ^“Western Asia”] OR [mh ^“Eastern Europe”] OR [mh ^“developing country”] |
|  | #2 | (afghanistan OR albania OR algeria OR "american samoa" OR angola OR "antigua and barbuda" OR antigua OR barbuda OR argentina OR armenia OR armenian OR aruba OR azerbaijan OR bahrain OR bangladesh OR barbados OR "republic of belarus" OR belarus OR byelarus OR belorussia OR byelorussian OR belize OR "british honduras" OR benin OR dahomey OR bhutan OR bolivia OR "bosnia and herzegovina" OR bosnia OR herzegovina OR botswana OR bechuanaland OR brazil OR brasil OR bulgaria OR "burkina faso" OR "burkina fasso" OR "upper volta" OR burundi OR urundi OR "cabo verde" OR "cape verde" OR cambodia OR kampuchea OR "khmer republic" OR cameroon OR cameron OR cameroun OR "central african republic" OR "ubangi shari" OR chad OR chile OR china OR colombia OR comoros OR "comoro islands" OR "iles comores" OR mayotte OR "democratic republic of the congo" OR "democratic republic congo" OR congo OR zaire OR "costa rica" OR "cote d’ivoire" OR "cote d’ ivoire" OR "cote divoire" OR "cote d ivoire" OR "ivory coast" OR croatia OR cuba OR cyprus OR "czech republic" OR czechoslovakia OR djibouti OR "french somaliland" OR dominica OR "dominican republic" OR ecuador OR egypt OR "united arab republic" OR "el salvador" OR "equatorial guinea" OR "spanish guinea" OR eritrea OR estonia OR eswatini OR swaziland OR ethiopia OR fiji OR gabon OR "gabonese republic" OR gambia OR "georgia (republic)" OR georgia OR georgian OR ghana OR "gold coast" OR gibraltar OR greece OR grenada OR guam OR guatemala OR guinea OR "guinea bissau" OR guyana OR "british guiana" OR haiti OR hispaniola OR honduras OR hungary OR india OR indonesia OR timor OR iran OR iraq OR "isle of man" OR jamaica OR jordan OR kazakhstan OR kazakh OR kenya OR "democratic people’s republic of korea" OR "republic of korea" OR north korea OR south korea OR korea OR kosovo OR kyrgyzstan OR kirghizia OR kirgizstan OR "kyrgyz republic" OR kirghiz OR laos OR "lao pdr" OR "lao people's democratic republic" OR latvia OR lebanon OR "lebanese republic" OR lesotho OR basutoland OR liberia OR libya OR "libyan arab jamahiriya" OR lithuania OR macau OR macao OR "republic of north macedonia" OR macedonia OR madagascar OR "malagasy republic" OR malawi OR nyasaland OR malaysia OR "malay federation" OR "malaya federation" OR maldives OR "indian ocean islands" OR "indian ocean" OR mali OR malta OR micronesia OR "federated states of micronesia" OR kiribati OR "marshall islands" OR nauru OR "northern mariana islands" OR palau OR tuvalu OR mauritania OR mauritius OR mexico OR moldova OR moldovian OR mongolia OR montenegro OR morocco OR ifni OR mozambique OR "portuguese east africa" OR myanmar OR burma OR namibia OR nepal OR "netherlands antilles" OR nicaragua OR niger OR nigeria OR oman OR muscat OR pakistan OR panama OR "papua new guinea" OR paraguay OR peru OR philippines OR philipines OR phillipines OR phillippines OR poland OR "polish people's republic" OR portugal OR "portuguese republic" OR "puerto rico" OR romania OR russia OR "russian federation" OR ussr OR "soviet union" OR "union of soviet socialist republics" OR rwanda OR ruanda OR samoa OR "pacific islands" OR polynesia OR "samoan islands" OR "navigator island" OR "navigator islands" OR "sao tome and principe" OR "saudi arabia" OR senegal OR serbia OR seychelles OR "sierra leone" OR slovakia OR "slovak republic" OR slovenia OR melanesia OR "solomon island" OR "solomon islands" OR "norfolk island" OR "norfolk islands" OR somalia OR "south africa" OR "south sudan" OR "sri lanka" OR ceylon OR "saint kitts and nevis" OR "st. kitts and nevis" OR "saint lucia" OR "st. lucia" OR "saint vincent and the grenadines" OR "saint vincent" OR "st. vincent" OR grenadines OR sudan OR suriname OR surinam OR "dutch guiana" OR "netherlands guiana" OR syria OR "syrian arab republic" OR tajikistan OR tadjikistan OR tadzhikistan OR tadzhik OR tanzania OR tanganyika OR thailand OR siam OR "timor leste" OR "east timor" OR togo OR "togolese republic" OR tonga OR "trinidad and tobago" OR trinidad OR tobago OR tunisia OR turkey OR turkmenistan OR turkmen OR uganda OR ukraine OR uruguay OR uzbekistan OR uzbek OR vanuatu OR "new hebrides" OR venezuela OR vietnam OR "viet nam" OR "middle east" OR "west bank" OR gaza OR palestine OR yemen OR yugoslavia OR zambia OR zimbabwe OR "northern rhodesia" OR "global south" OR "africa south of the sahara" OR "sub saharan africa" OR "subsaharan africa" OR "africa, central" OR "central africa" OR "africa, northern$"$ OR "north africa" OR "northern africa" OR magreb OR maghrib OR sahara OR "africa, southern$"$ OR "southern africa" OR "africa, eastern$"$ OR "east africa" OR "eastern africa" OR "africa, western$"$ OR $"$west africa$"$ OR $"$western africa$"$ OR "west indies" OR "indian ocean islands" OR caribbean OR "central america" OR "latin america" OR "south and central america" OR "south america" OR "asia, central" OR "central asia" OR "asia, northern" OR "north asia" OR "northern asia" OR "asia, southeastern" OR "southeastern asia" OR "south eastern asia" OR "southeast asia" OR "south east asia" OR "asia, western" OR "western asia" OR "europe, eastern" OR "east europe" OR "eastern europe" OR "developing country" OR "developing countries" OR "developing nation" OR "developing nations" OR "developing population" OR "developing populations" OR "developing world" OR "less developed country" OR "less developed countries" OR "less developed nation" OR "less developed nations" OR "less developed population" OR "less developed populations" OR "less developed world" OR "lesser developed country" OR "lesser developed countries" OR "lesser developed nation" OR "lesser developed nations" OR "lesser developed population" OR "lesser developed populations" OR "lesser developed world" OR "under developed country" OR "under developed countries" OR "under developed nation" OR "under developed nations" OR "under developed population" OR "under developed populations" OR "under developed world" OR "underdeveloped country" OR "underdeveloped countries" OR "underdeveloped nation" OR "underdeveloped nations" OR "underdeveloped population" OR "underdeveloped populations" OR "underdeveloped world" OR "middle income country" OR "middle income countries" OR "middle income nation" OR "middle income nations" OR "middle income population" OR "middle income populations" OR "low income country" OR "low income countries" OR "low income nation" OR "low income nations" OR "low income population" OR "low income populations" OR "lower income country" OR "lower income countries" OR "lower income nation" OR "lower income nations" OR "lower income population" OR "lower income populations" OR "underserved country" OR "underserved countries" OR "underserved nation" OR "underserved nations" OR "underserved population" OR "underserved populations" OR "underserved world" OR "under served country" OR "under served countries" OR "under served nation" OR "under served nations" OR "under served population" OR "under served populations" OR "under served world" OR "deprived country" OR "deprived countries" OR "deprived nation" OR "deprived nations" OR "deprived population" OR "deprived populations" OR "deprived world" OR "poor country" OR "poor countries" OR "poor nation" OR "poor nations" OR "poor population" OR "poor populations" OR "poor world" OR "poorer country" OR "poorer countries" OR "poorer nation" OR "poorer nations" OR "poorer population" OR "poorer populations" OR "poorer world" OR "developing economy" OR "developing economies" OR "less developed economy" OR "less developed economies" OR "lesser developed economy" OR "lesser developed economies" OR "under developed economy" OR "under developed economies" OR "underdeveloped economy" OR "underdeveloped economies" OR "middle income economy" OR "middle income economies" OR "low income economy" OR "low income economies" OR "lower income economy" OR "lower income economies" OR "low gdp" OR "low gnp" OR "low gross domestic" OR "low gross national" OR "lower gdp" OR "lower gnp" OR "lower gross domestic" OR "lower gross national" OR lmic OR lmics OR "third world" OR "lami country" OR "lami countries" OR "transitional country" OR "transitional countries" OR "emerging economies" OR "emerging nation" OR "emerging nations"):ti,ab,kw |
|  | #1 | (afghan OR afghans OR afghani OR albanian OR albanians OR algerian OR algerians OR "american samoan" OR "american samoans" OR angolan OR angolans OR antiguan OR antiguans OR barbudan OR berbudans OR argentine OR argentines OR argentinian OR argentinians OR argentinean OR argentineans OR armenian OR armenians OR aruban OR arubans OR azerbaijani OR azerbaijanis OR bahraini OR bahrainis OR bangladeshi OR bangladeshis OR bangalees OR bajan OR bajans OR belarusian OR belarusians OR byelorussian OR byelorussians OR belizean OR belizeans OR beninese OR benineses OR bhutanese OR bolivian OR bolivians OR bosnian OR bosnians OR botswana OR batswana OR brazilian OR brazilians OR brasilian OR brasilians OR bulgarian OR bulgarians OR burkinabe OR burkinese OR burundian OR burundians OR "cape verdean" OR "cape verdeans" OR "cabo verdean" OR "cabo verdeans" OR cambodian OR cambodians OR khmer OR cameroonian OR cameroonians OR "central african" OR "central africans" OR chadian OR chadians OR chilean OR chileans OR chinese OR colombian OR colombians OR comorian OR comorians OR congolese OR "costa rican" OR "costa ricans" OR ivorian OR ivorians OR croatian OR croatians OR cuban OR cubans OR cypriot OR cypriots OR czech OR czechs OR djiboutian OR djiboutians OR dominican OR dominicans OR ecuadorian OR ecuadorians OR egyptian OR egyptians OR salvadoran OR salvadorans OR "equatorial guinean" OR "equatorial guineans" OR equatoguinean OR equatoguineans OR eritrean OR eritreans OR estonian OR estonians OR swazi OR swazis OR swati OR swatis OR ethiopian OR ethiopians OR fijian OR fijians OR gabonese OR gabonaise OR gambian OR gambians OR georgian OR georgians OR ghanaian OR ghanaians OR gibraltarian OR gibraltarians OR greek OR greeks OR grenadian OR grenadians OR guamanian OR guamanians OR guatemalan OR guatemalans OR guinean OR guineans OR "bissau guinean" OR "bissau guineans" OR guyanese OR haitian OR haitians OR honduran OR hondurans OR hungarian OR hungarians OR indian OR indians OR indonesian OR indonesians OR iranian OR iranians OR iraqian OR iraqians OR iraqi OR iraqis OR manx OR jamaican OR jamaicans OR jordanian OR jordanians OR kazakhstani OR kazakhstanis OR kenyan OR kenyans OR kirabati OR kirabatian OR kirabatians OR "north korean" OR "north koreans" OR korean OR koreans OR kosovar OR kosovars OR kosovan OR kosovans OR kyrgyzstani OR kyrgyzstanis OR kyrgyz OR lao OR laotian OR laotians OR latvian OR latvians OR lebanese OR lesothan OR lesothans OR lesothonian OR lesothonians OR mosotho OR basotho OR liberian OR liberians OR libyan OR libyans OR lithuanian OR lithuanians OR macanese OR macedonian OR macedonians OR malagasy OR madagascan OR madagascans OR malawian OR malawians OR malaysian OR malaysians OR maldivian OR maldivians OR malian OR malians OR maltese OR marshallese OR marshalleses OR mauritanian OR mauritanians OR mauritian OR mauritians OR mexican OR mexicans OR micronesian OR micronesians OR moldovan OR moldovans OR mongolian OR mongolians OR mongol OR montenegrin OR montenegrins OR moroccan OR moroccans OR mozambican OR mozambicans OR burmese OR myanma OR namibian OR namibians OR nauruan OR nauruans OR nepali OR nepalese OR "netherlands antillean" OR "netherlands antilleans" OR nicaraguan OR nicaraguans OR nigerien OR nigeriens OR nigerian OR nigerians OR "northern mariana islander" OR "northern mariana islanders" OR mariana OR marianas OR omani OR omanis OR pakistani OR pakistanis OR palauan OR palauans OR panamanian OR panamanians OR "papua new guinean" OR "papua new guineans" OR paraguayan OR paraguayans OR peruvian OR peruvians OR philippine OR philippines OR philipine OR philipines OR phillipine OR phillipines OR phillippine OR phillippines OR filipino OR filipinos OR filipina OR filipinas OR polish OR pole OR poles OR portuguese OR "puerto rican" OR "puerto ricans" OR romanian OR romanians OR russian OR russians OR "soviet people" OR "soviet population" OR rwandan OR rwandans OR rwandese OR ruandan OR ruandans OR ruandese OR samoan OR samoans OR "sao tomean" OR "sao tomeans" OR santomean OR santomeans OR "saudi arabian" OR "saudi arabians" OR saudi OR saudis OR senegalese OR serbian OR serbians OR montenegrin OR montenegrins OR seychellois OR seychelloise OR seychelloises OR "sierra leonean" OR "sierra leoneans" OR slovak OR slovaks OR slovene OR slovenes OR "solomon islander" OR "solomon islanders" OR somali OR somalis OR "south african" OR "south africans" OR "south sudanese" OR "sri lankan" OR "sri lankans" OR ceylonese OR kittitian OR kittitians OR nevisian OR nevisians OR "saint lucian" OR "saint lucians" OR vincentian OR vincentians OR sudanese OR surinamese OR surinameses OR syrian OR syrians OR tajik OR tajiks OR tajikistani OR tajikistanis OR tanzanian OR tanzanians OR tanganyikan OR tanganyikans OR thai OR timorese OR timoreses OR togolese OR tongan OR tongans OR trinidadian OR trinidadians OR tobagonian OR tobagonians OR tunisian OR tunisians OR turk OR turks OR turkish OR turkmen OR turkmens OR tuvaluan OR tuvaluans OR ugandan OR ugandans OR ukrainian OR ukrainians uruguayan OR uruguayans OR uzbek OR uzbeks OR vanuatu OR vanuatuan OR vanuatuans OR venezuelan OR venezuelans OR vietnamese OR yemeni OR yemenis OR yemenite OR yemenites OR yemenese OR yugoslav OR yugoslavs OR yugoslavian OR yugoslavians OR zambian OR zambians OR zimbabwean OR zimbabweans):ti,ab,kw |
| **Concept 6**: study design | 6 | "randomized controlled trial"[pt] OR "controlled clinical trial"[pt] OR "randomized"[TIAB] OR placebo[tiab] OR "drug therapy"[SH]OR "randomly"[tiab] OR "trial"[tiab] OR "groups"[tiab] NOT ("animals"[mh] NOT "humans"[mh]) |
