# Supplementary material for: Effects of nutritional interventions on nutritional and immunological status and adherence to antiretroviral treatment among adults living with HIV in low- and middle-income countries: Systematic review and meta-analysis
Source: PLoS One. 2025 Jun 3;20(6):e0319843. doi: 10.1371/journal.pone.0319843 (PMC12132990; doi:10.1371/journal.pone.0319843)
Supplement: S4 Table — (DOCX) [file pone.0319843.s005.docx]

| **S4 Table.** Scopus search strategy for the effects of nutritional interventions on nutritional status and health of people living with HIV/AIDS. *(Research question: In adults living with HIV/AIDS, in low- and middle-income countries(P), how nutritional and medical care (I) compared to medical care only (C), could improve nutritional status, adherence and response to antiretroviral therapy (ART) (O).* | | |
| --- | --- | --- |
| **NAME OF DATABASE (interface):** Scopus (via the Scopus.com) | | |
| **Concept** | **Line number** | **Search strategy** |
| **Concept 1**:  Acquired immunodeficiency syndrome | acquired immunodeficiency syndrome | INDEXTERMS ("acquired immunodeficiency syndrome" OR "AIDS") OR TITLE-ABS-KEY (("acquired" AND "immunodeficiency" AND "syndrome*") OR "AIDS" ) AND NOT (INDEXTERMS("audiovisual aids") OR TITLE-ABS-KEY (("audiovisual" AND "aids") OR ("visual" AND "aids"))) |
|  | HIV Infections | INDEXTERMS ("HIV Infections" OR "hiv") OR TITLE-ABS-KEY ("hiv" OR ("human" AND "immunodeficiency" AND "virus")) |
|  | Acute Retroviral Syndrome | INDEXTERMS ("acute retroviral syndrome") OR TITLE-ABS-KEY ("acute" AND "retroviral" AND "syndrome") |
| **Concept 2:**  Nutritional intervention | Diet therapy | INDEXTERMS ("diet therapy" OR "diet intervention" OR "diet treatment" OR "dietary therapy" OR "dietary intervention" OR "dietary treatment") OR TITLE-ABS-KEY (("diet*" AND "therapy") OR ("diet*" AND "treatment") OR ("diet*" AND "intervention")) |
|  | Food basket | TITLE-ABS-KEY ("food" AND "basket*") |
|  | Food supplement | INDEXTERMS ("dietary supplements" OR "supplementation") OR TITLE-ABS-KEY (("food" OR "diet*") AND "supplement*") |
|  | Micronutrient supplementation | (INDEXTERMS ("micronutrients" OR "trace elements") OR TITLE-ABS-KEY ("micronutrient*" OR "micronutriment*" OR ("trace" AND "element*"))) AND (INDEXTERMS ("supplementation") OR TITLE-ABS-KEY ("supplement*" OR "multiple*")) |
|  | Multiple micronutrient powder | INDEXTERMS (("micronutrients" OR "trace elements") AND "powders") OR TITLE-ABS-KEY (("micronutrient*" OR "micronutriment*" OR ("trace" AND "elements")) AND "powder*") |
|  | Macronutrient supplementation | (INDEXTERMS ("macronutrients") OR TITLE-ABS-KEY ("macronutrient*")) AND (INDEXTERMS ("supplementation") OR TITLE-ABS-KEY ("supplement*")) |
|  | Protein supplementation | (INDEXTERMS ("proteins") OR TITLE-ABS-KEY ("protein*")) AND (INDEXTERMS ("supplementation") OR TITLE-ABS-KEY ("supplement*")) |
|  | High protein diet | INDEXTERMS ("Diet, High-Protein") OR TITLE-ABS-KEY (("diet" AND "high" AND "protein") OR ("protein-rich" AND "food*") OR ("protein rich" AND "food*")) |
|  | Amino acid supplementation | (INDEXTERMS ("amino acids") OR TITLE-ABS-KEY ("amino" AND "acid*")) AND (INDEXTERMS ("supplementation") OR TITLE-ABS-KEY ("supplement*")) |
|  | Legumes | INDEXTERMS ("fabaceae" OR "legume") OR TITLE-ABS-KEY ("fabaceae" OR "legume*") |
|  | Spirulina | INDEXTERMS ("spirulina") OR TITLE-ABS-KEY ("spirulina") |
|  | Alga supplementation | (INDEXTERMS ("algal proteins") OR TITLE-ABS-KEY ("alga*")) AND (INDEXTERMS ("supplementation") OR TITLE-ABS-KEY ("supplement*")) |
|  | Animal source food | INDEXTERMS ("animals" AND "food") OR TITLE-ABS-KEY ("animal" AND "source*" AND "food*") |
|  | Meat consumption | (INDEXTERMS ("meat") OR TITLE-ABS-KEY ("meat")) AND TITLE-ABS-KEY ("consumption*" OR "intake*") |
|  | Fish intake | (INDEXTERMS ("fishes") OR TITLE-ABS-KEY ("fish*")) AND TITLE-ABS-KEY ("consumption*" OR "intake*") |
|  | Egg intake | (INDEXTERMS ("ovum") OR TITLE-ABS-KEY ("ovum" OR "egg" OR "eggs")) AND TITLE-ABS-KEY ("consumption*" OR "intake*") |
|  | Soya flour | INDEXTERMS ("soybeans" AND "flour") OR TITLE-ABS-KEY ( ("soybeans" OR "soya") AND "flour*") |
|  | Corn Soya blend | (INDEXTERMS ("zea mays" AND "soybeans") OR TITLE-ABS-KEY (("maize*" OR ("zea" AND "mays") OR "corn") AND ("soybeans" OR "soya"))) AND (TITLE-ABS-KEY ("blend*")) OR TITLE-ABS-KEY ("CSB*") |
|  | Wheat soya blend | (INDEXTERMS ("triticum" AND "soybeans") OR TITLE-ABS-KEY (("triticum" OR "wheat*") AND "soya")) AND (TITLE-ABS-KEY ("blend*")) OR TITLE-ABS-KEY ("WSB*") |
|  | Nutritional rehabilitation | (INDEXTERMS ("rehabilitation") OR TITLE-ABS-KEY ("rehabilit*")) AND TITLE-ABS-KEY("nutrition*" OR "nutritive") |
|  | Lipid-based nutrient supplements | (INDEXTERMS ("nutrients") OR TITLE-ABS-KEY ("nutrient*")) AND TITLE-ABS-KEY ("lipid-based” OR "lipid based”) |
|  | Nutrititional support | INDEXTERMS ("nutritional support" OR "nutrition therapy") OR TITLE-ABS-KEY (("nutrition*" AND "support") OR ("nutrition*" AND "therapy")) |
|  |  |  |
|  | Fortified Food | INDEXTERMS ("food, fortified") OR TITLE-ABS-KEY ("food*" AND "fortifi*") |
| **Concept 3**:  Nutritional status | Nutritional status | INDEXTERMS ("nutritional status") OR TITLE-ABS-KEY ("nutrition*"AND "status") |
|  | Body composition | INDEXTERMS ("body composition") OR TITLE-ABS-KEY ("body" AND "composition") |
|  | Body Weight gain/Body weight loss/underweight/wasting/undernutrition | INDEXTERMS **("**body weight gain" OR "body weight increase" OR "body weight loss" OR "thinness" OR "cachexia" OR "wasting syndrome" OR "body weight decrease" OR "weight insufficiency" OR "deficient nutrition") OR TITLE-ABS-KEY (("body" AND "weight" AND "gain") OR ("body" AND "weight" AND "loss") OR "thinness" OR "underweight*" OR "cachexia" OR "wasting" OR "undernutrition*") |
|  | Lean mass/ Fat free mass | TITLE-ABS-KEY (("lean" AND "mass") OR ("fat" AND "free" AND "mass")) |
|  | Micronutrient deficiencies | INDEXTERMS (("micronutrients" OR "trace elements") AND "deficiency") OR TITLE-ABS-KEY (("micronutrient*" OR "micronutriment*" OR ("trace" AND "element*")) AND "deficien*") |
|  | Anorexia | INDEXTERMS ("anorexia") OR TITLE-ABS-KEY ("anorexi*") |
|  | Acute malnutrition | (INDEXTERMS ("malnutrition") OR TITLE-ABS-KEY ("malnutrition*" OR "malnourish*")) AND TITLE-ABS-KEY ("acute*") |
|  | Body mass index | INDEXTERMS ("body mass index") OR TITLE-ABS-KEY ("body" AND "mass" AND "index") |
|  | Emaciation | INDEXTERMS ("emaciation") OR TITLE-ABS-KEY ("emaciat*") |
| **Concept 4:**  Adherence and response to ART | HIV drug side effects | INDEXTERMS ("hiv" AND "drug related side effects and adverse reactions") OR TITLE-ABS-KEY ("hiv" AND "drug" AND "side" AND "effect*") |
|  | Antiretroviral adherence | (INDEXTERMS ("anti retroviral agents") OR TITLE-ABS-KEY ("anti retroviral" OR "antiretroviral*" OR "arv")) AND TITLE-ABS-KEY ("adher*") |
|  | Viral load | INDEXTERMS ("viral load") OR TITLE-ABS-KEY ("viral" AND "load") |
|  | CD3 and CD4 | TITLE-ABS-KEY ("CD3" OR "CD4") |
| **Concept 5**:  Low and middle income countries  **#1b OR #2** | #1b | INDEXTERMS (“Afghanistan” OR “Albania” OR “Algeria” OR “American Samoa” OR “Angola” OR “Antigua and Barbuda” OR “Argentina” OR “Armenia” OR “Aruba” OR “Azerbaijan” OR “Bahrain” OR “Bangladesh” OR “Barbados” OR “Belarus” OR “Belize” OR “Benin” OR “Bhutan” OR “Botswana” OR “Bolivia” OR “Bosnia and Herzegovina” OR “Brazil” OR “Bulgaria” OR “Burkina Faso” OR “Burundi” OR “Cape Verde” OR “Cambodia” OR “Cameroon” OR “Central African Republic” OR “Chad” OR “Chile” OR “Colombia” OR “China” OR “Comoros” OR “Mayotte” OR “Democratic Republic Congo” OR “congo” OR “Costa Rica” OR “cote d` Ivoire” OR “Croatia” OR “Cuba” OR “Cyprus” OR “Czech Republic” OR “Czechoslovakia” OR “Djibouti” OR “Somaliland” OR “Dominican Republic” OR “Dominica” OR “Ecuador” OR “Egypt” OR “El Salvador” OR “Equatorial Guinea” OR “Eritrea” OR “Estonia” OR “Eswatini” OR “Ethiopia” OR “Fiji” OR “Gabon” OR “Gambia” OR “Georgia Republic” OR “Georgia” OR “Ghana” OR “Gibraltar” OR “Greece” OR “Grenada” OR “Guam” OR “Guatemala” OR “Guinea” OR “Guinea-Bissau” OR “Guyana” OR “French Guyana” OR “British Guiana” OR “Haiti” OR “Honduras” OR “Hungary” OR “India” OR “Indonesia” OR "Timor-Leste” OR “Iran” OR “Iraq” OR “Isle of Man” OR “Jamaica” OR “Jordan” OR “Kazakhstan” OR “Kenya" OR “North Korea” OR “South Korea” OR “Kosovo” OR “Kyrgyzstan” OR “Laos” OR “Latvia” OR “Lebanon” OR “Lesotho” OR “Liberia” OR “Libyan Arab Jamahiriya” OR “Lithuania” OR “Macao” OR “Republic of North Macedonia” OR “Madagascar” OR “Malawi” OR “Malaysia” OR “Indian Ocean” OR “Mali” OR “Malta” OR “Federated States of Micronesia” OR “Kiribati” OR “marshall islands” OR “Nauru” OR “Northern Mariana Islands” OR “palau” OR “Tuvalu” OR “Mauritania” OR “Mauritius” OR “Moldova” OR “Mexico” OR “Mongolia” OR “montenegro republic” OR “Montenegro (republic)” OR “Morocco” OR “Mozambique” OR “Myanmar” OR “Namibia” OR “Nepal” OR “Netherlands Antilles” OR “Nicaragua” OR “Niger” OR “Nigeria” OR “Oman” OR “Pakistan” OR “Panama” OR “Papua New Guinea” OR “Paraguay” OR “Peru” OR “Philippines” OR “Poland” OR “Portugal” OR “Puerto Rico” OR “Romania” OR “Russian Federation” OR “USSR” OR “Rwanda” OR “Samoa” OR “Samoan Islands” OR “Polynesia” OR “Sao Tome and Principe” OR “Saudi Arabia” OR “Senegal” OR “Serbia” OR “Seychelles” OR “Sierra Leone” OR “Slovakia” OR “Slovenia” OR “Melanesia” OR “Solomon Islands” OR “Norfolk Island” OR “Somalia” OR “South Africa” OR “South Sudan” OR “Sri Lanka” OR “Saint Kitts and Nevis” OR “Saint Lucia” OR “Saint Vincent and the Grenadines” OR “Sudan” OR “Suriname” OR “Suriname" OR “Syrian Arab Republic” OR “Tajikistan” OR “Tanzania” OR “Thailand” OR “Timor-Leste” OR “Togo” OR “Tonga” OR “Trinidad and Tobago” OR “Tunisia” OR “Turkey Republic” OR “Turkey (Republic)” OR “Turkmenistan” OR “Uganda” OR “Ukraine” OR “Uruguay” OR “Uzbekistan” OR “Vanuatu” OR “Venezuela” OR “Viet nam” OR “Middle East” OR “Gaza Strip Palestine” OR “Palestine” OR “Yemen” OR “Yugoslavia” OR “Zambia” OR “Zimbabwe”OR “Africa south of the Sahara” OR “Central Africa” OR “Indian Ocean” OR “Caribbean” OR “Central America” OR “South and Central America” OR “South America” OR “Central Asia” OR “Northern Asia” OR “Southeast Asia” OR “Western Asia” OR “Eastern Europe” OR “developing country”) |
|  | #2 | TITLE-ABS-KEY (“afghan*” OR “albania*” OR “algeria*” OR “american samoa*” OR “angola*” OR “antigua*” OR “barbuda*” OR “argentin*” OR “armenia*” OR “aruba*” OR “azerbaijan*” OR “bahrain*” OR “bangladesh*” OR “bangalees” OR “barbados*” OR “bajan*” OR “belarus*” OR “republic of Belarus” OR “byelarus*” OR “belorussia*” OR “byelorussian*” OR “belize*” OR “british honduras*” OR “benin*” OR “dahomey*” OR “bhutan*” OR “bolivia*” OR “Bosnia and Herzegovina” OR “bosnia*” OR “herzegovina*” OR “botswana*” OR “batswana*” OR “bechuanaland*” OR “brazil*” OR “brasil*” OR “bulgaria*” OR “burkina fasso*” OR “burkinabe*” OR “burkinese*” OR “upper volta*” OR “burundi*” OR “urundi*” OR “cabo verde*” OR “cape verde*” OR “cambodia*” OR “Kampuchea” OR “khmer republic” OR “khmer” OR “cameroon*” OR “Cameron” OR “cameroun” OR “central african republic” OR “central african*” OR “ubangi shari” OR “chad*” OR “chile” OR “china” OR “Chinese” OR “colombia*” OR “comoros” OR “comoro islands” OR “iles comores” OR “comorian*” OR “mayotte” OR “democratic republic of the congo” OR “congo*” OR “zaire” OR “costa rica*” OR “cote d` Ivoire” OR “cote d` Ivoire” OR “cote divoire” OR “cote d ivoire” OR “ivory coast” OR “ivorian*” OR “crotia*” OR “cuba*” OR “cyprus” OR “cypriot*” OR “czech*” OR “Czechoslovakia” OR “djibouti*” OR “french Somaliland” OR “dominica*” OR “ecuador*” OR “egypt*” OR “united arab republic” OR “el Salvador” OR “salvadoran*” OR “equatorial guinea*” OR “equatoguinean*” OR “spanish guinea” OR “eritrea*” OR “estonia*” OR “eswatini” OR “Swaziland” OR “swazi*” OR “swati*” OR “ethiopia*” OR “fiji*” OR “gabon*” OR “gabonese republic” OR “gambia*” OR “georgia*” OR “ghana*” OR “gold coast” OR “gibraltar*” OR “Greece” OR “greek*” OR “grenada” OR “grenadian*” OR “guam*” OR “guatemala*” OR “guinea*” OR “Guinea-Bissau” OR “guinea Bissau” OR “Guyana” OR “Guyanese” OR “haiti*” OR “Hispaniola” OR “Honduras” OR “honduran*” OR “hungary” OR “hungarian*” OR “india*” OR “indonesia*” OR “timor” OR “iran*” OR “iraq*” OR “isle of man” OR “manx” OR “jamaica*” OR “jordan*” OR “kazakh*” OR “kenya*” OR “kirabati*” OR “north korea*” OR “democratic people` s republic of korea” OR “republic of korea” OR “south korea” OR “korea*” OR “Kosovo” OR “kosovar*” OR “kosovan*” OR “kyrgyzstan*” OR “Kirghizia” OR “Kirgizstan” OR “kyrgyz republic” OR “kirghiz” OR “Kyrgyz” OR “laos” OR “lao” OR “laotian*” OR “lao pdr” OR “lao people` s democratic republic” OR “latvia*” OR “Lebanon” OR “lebanese republic” OR “Lebanese” OR “lesotho*” OR “lesothan*” OR “mosotho*” OR “Basutoland” OR “Basotho” OR “liberia*” OR “libya*” OR “lithuania*” OR “macau*” OR “macao*” OR “Macanese” OR “macedonia*” OR “Madagascar” OR “Malagasy” OR “madagascan*” OR “malawi*” OR “Nyasaland” OR “malaysia*” OR “malay federation” OR “malaya federation” OR “Maldives” OR “maldivian*” OR “indian ocean islands” OR “indian ocean” OR “mali*” OR “malta*” OR “maltese” OR “micronesia*” OR “kiribati*” OR “marshall islands*” OR “marshallese**” OR “nauru*” OR “northern mariana islands” OR “palau” OR “Tuvalu” OR “mauritania*” OR “mauritius*” OR “mauritian*” OR “Mexico” OR “mexican*” OR “moldova*” OR “moldovian*” OR “mongolia*” OR “mongol” OR “Montenegro” OR “montenegrin*” OR “morocco” OR “moroccan*” OR “ifni” OR “Mozambique” OR “mozambican*” OR “portuguese east Africa” OR “myanma*” OR “Burma” OR “Burmese” OR “namibia*” OR “nauruan*” OR “nepal*” OR “netherlands antille*” OR “nicaragua*” OR “niger*” OR “nigeria*” OR “northern mariana inslander*” OR “mariana*” OR “oman*” OR “muscat” OR “pakistan*” OR “panama*” OR “papua new guinea*” OR “new guinea*” OR “palauan*” OR “palnamian*” OR “paraguay*” OR “peru*” OR “philippine*” OR “philipine*” OR “phillipine*” OR “phillippine*” OR “filipin*” OR “poland*” OR “polish people` s republic” OR “polish” OR “pole*” OR “portugal*” OR “portuguese republic” OR “Portuguese” OR “puerto rico*” OR “puerto rican*” OR “romania*” OR “russia*” OR “ussr” OR “soviet union” OR “union of soviet socialist republics” OR “soviet people” OR “soviet population” OR “rwanda*” OR “Rwandese” OR “ruanda*” OR “ruandese” OR “samoa*” OR “pacific islands” OR “polynesia*” OR “navigator island*” OR “sao tome and principe” OR “sao tomean*” OR “santomean*” OR “saudi arabia*” OR “saudi*” OR “senegal*” OR “serbia*” OR “seychell*” OR “sierra leone*” OR “slovakia*” OR “lovak*” OR “slovak republic” OR “slovenia*” OR “slovene*” OR “melanesia*” OR “solomon island*” OR “norfolk island*” OR “somali*” OR “south africa*” OR “south sudan*” OR “sri lanka*” OR “ceylon*” OR “saint kitts and nevis” OR “st. kitts and nevis” OR “kittitian*” OR “nevisian*” OR “saint lucia*” OR “st. lucia” OR “saint vincent and the grenadines” OR “saint Vincent” OR “st. Vincent” OR “grenadines” OR “vincentian*” OR “sudan*” OR “surinam*” OR “surinam*” OR “dutch Guiana” OR “netherlands Guiana” OR “syria*” OR “tajik*” OR “tadjikistan*” OR “Tadzhikistan” OR “Tadzhik” OR “tanzania*” OR “tanganyika*” OR “Thailand” OR “siam” OR “timor leste” OR “timor-leste” OR “timorese*” OR “east timor” OR “togo*” OR “togolese republic” OR “tonga*” OR “trinidad and Tobago” OR “trinidad*” OR “tobago*” OR “tunisia*” OR “turkey” OR “turk*” OR “Turkmenistan” OR “turkmen*” OR “tuvaluan*” OR “uganda*” OR “ukrain*” OR “uruguay*” OR “uzbek*” OR “vanuatu*” OR “new Hebrides” OR “venezuela*” OR “vietnam*” OR “viet nam” OR “middle east” OR “west bank” OR “gaze” OR “Palestine” OR “yemen*” OR “yugoslav*” OR “zambia*” OR “zimbabwe*” OR “northern rhodesia*” OR “global south” OR “africa south of the sahara” OR “sub sahara Africa” OR “subsaharan Africa” OR “central Africa” OR “africa, central” OR “africa, northern” OR “north Africa” OR “northern Africa” OR “magreb” OR “maghrib” OR “sahara” OR “africa, southern” OR “africa, southern” OR “southern africa*” OR “africa, eastern” OR “east africa*” OR “eastern africa*” OR “africa, western” OR “west africa*” OR “western africa*” OR “west indies” OR “indian ocean islands” OR “caribbean region” OR “'caribbean” OR “caribbean islands” OR “central america*” OR “south and central America” OR “latin america*” OR “south America” OR “central asia*” OR “northern asia*” OR “north asia*” OR “southeastern asia*” OR “south eastern asia*” OR “southeast asia*” OR “south east asia*” OR “western asia*” OR “west asia*” OR “eastern europe*” OR “east europe*” OR “developing countr*” OR “developing nation*” OR “developing population*” OR “developing world” OR “less developed countr*” OR “less developed nation*” OR “less developed population*” OR “less developed world” OR “lesser developed countr*” OR “lesser developed nation*” OR “lesser developed population*” OR “lesser developed world” OR “under developed countr*” OR “under developed nation*” OR “under developed population*” OR “under developed world” OR “underdeveloped countr*” OR “underdeveloped nation*” OR “underdeveloped population*” OR “underdeveloped world” OR “middle income countr*” OR “middle income nation*” OR “middle income population*” OR “low income countr*” OR “low income nation*” OR “low income population*” OR “lower income countr*” OR “lower income nation*” OR “lower income population*” OR “underserved countr*” OR “underserved nation*” OR “underserved population*” OR “underserved world” OR “under served countr*” OR “under served nation*” OR “under served population*” OR “under served world” OR “deprived countr*” OR “deprived nation*” OR “deprived population*” OR “deprived world” OR “poor countr*” OR “poor nation*” OR “poor population*” OR “poor world” OR “poorer countr*” OR “poorer nation*” OR “poorer population*” OR “poorer world” OR “developing econom*” OR “less developed econom*” OR “lesser developed econom*” OR “under developed econom*” OR “underdeveloped econom*” OR “middle income econom*” OR “low income econom*” OR “lower income econom*” OR “low gdp” OR “low gnp” OR “low gross domestic” OR “low gross national” OR “lower gdp” OR “lower gnp” OR “lower gross domestic” OR “lower gross national” OR “lmic” OR “lmics” OR “third world” OR “lami countr*” OR “transitional countr*” OR “emerging economies” OR “emerging nation*” OR “least developed countr*” OR “low and middle income countr*”) |
| **Concept 6**: study design and population age | 6 | ( INDEXTERMS ( "clinical trial*" OR "randomized controlled trial*" OR "controlled clinical trial*" OR "random allocation" OR "Double-Blind Method" OR "Single-Blind Method" OR "Cross-Over Studies" OR "Placebo*" OR "multicenter study" OR "double blind procedure" OR "single blind procedure" OR "crossover procedure" OR "controlled study" OR "randomization" ) ) OR ( TITLE-ABS-KEY ( "clinical trial*" OR "randomized controlled trial*" OR "controlled clinical trial*" OR “control group stud*” OR "random allocation" OR "randomly allocated" OR "allocated randomly" OR "Cross-Over Studies" OR “crossover trials” OR "Placebo*" OR "cross-over trial*" OR "single blind" OR "double blind" OR "factorial design" OR "factorial trial*" OR ( repeated AND cross-sectional ) ) ) OR ( TITLE-ABS ( (clinical AND trial*) OR trial* OR rct* OR random* OR blind* OR double-masked OR single-masked OR “control group*” OR “experimental stud*” OR “quasi experimental” OR “quasi experimental stud*”) ) |
