# Supplementary material for: Effects of nutritional interventions on nutritional and immunological status and adherence to antiretroviral treatment among adults living with HIV in low- and middle-income countries: Systematic review and meta-analysis
Source: PLoS One. 2025 Jun 3;20(6):e0319843. doi: 10.1371/journal.pone.0319843 (PMC12132990; doi:10.1371/journal.pone.0319843)
Supplement: S5 Table — (DOCX) [file pone.0319843.s006.docx]

| **S5 Table.** Web of Science search strategy for the effects of nutritional interventions on nutritional status and health of people living with HIV/AIDS. *(Research question: In adults living with HIV/AIDS, in low- and middle-income countries(P), how nutritional and medical care (I) compared to medical care only (C), could improve nutritional status, adherence and response to antiretroviral therapy (ART) (O).* | | |
| --- | --- | --- |
| **NAME OF DATABASE (interface):** Web of Science (via the apps.webofknowledge.com) | | |
| **Concept** | **Line number** | **Search strategy** |
| Concept 1:  Acquired immunodeficiency syndrome | acquired immunodeficiency syndrome | Ts=("acquired" AND "immunodeficiency" AND "syndrome*") OR (TS="AIDS" NOT (Ts=("audiovisual" AND "aids") OR Ts=("visual" AND "aids"))) |
|  | HIV Infections | Ts= "hiv" OR Ts= ("human" AND "immunodeficiency" AND "virus") |
|  | Acute Retroviral Syndrome | Ts=("acute" AND "retroviral" AND "syndrome") |
| Concept 2:  Nutritional intervention | Diet therapy | Ts=("diet*" AND "therapy") OR Ts=("diet*" AND "treatment*") OR Ts=("diet*" AND "intervention") |
|  | Food basket | Ts=("food*" AND "basket*") |
|  | Food supplement | TS=("diet*" AND "supplement*") OR TS=("food*" AND "supplement*") |
|  | Micronutrient supplementation | Ts=("micronutrient*" AND "supplement*") OR Ts=("micronutriment*" AND "supplement*") OR Ts=("trace" AND "element*" AND "supplement*") |
|  | Multiple micronutrient powder | Ts=("multiple*" AND "micronutrient*") OR Ts=("multiple*" AND "micronutriment*") OR Ts=("multiple*" AND "trace" AND "element*") OR Ts=("micronutrient*" AND "powder*")  OR Ts=("micronutriment*" AND "powder*") OR Ts=("trace" AND "element*" AND "powder*") |
|  | Macronutrient supplementation | Ts= ("macronutrient*" AND "supplement*") OR Ts=("macro-nutrient*" AND "supplement*") OR Ts=("nutrient*" AND "supplement*") |
|  | Protein supplementation | Ts=("protein*" AND "supplement*") |
|  | High protein diet | Ts=("diet*" AND "high" AND "protein") OR Ts=("food*" AND "rich" AND "protein") |
|  | Amino acid supplementation | Ts=("amino" AND "acid*" AND "supplement*") |
|  | Legumes | Ts="fabaceae" OR Ts="legume*" |
|  | Spirulina | Ts="spirulina" OR Ts="spiruline" |
|  | Alga supplementation | Ts= ("alga*" AND "supplement*") |
|  | Animal source food | Ts=("animal" AND "source*" AND "food*") |
|  | Meat consumption | Ts= ("meat" AND "consumption*") OR Ts=("meat" AND "intake*") |
|  | Fish intake | Ts= ("fish*" AND "consumption*") OR Ts= ("fish*" AND "intake*") |
|  | Egg intake | Ts= ("egg" AND "consumption*") OR Ts=("egg" AND "intake*") OR Ts= ("eggs" AND "consumption*") OR Ts=("eggs" AND "intake*") OR Ts= ("ovum" AND "consumption*") OR Ts=("ovum" AND "intake*") |
|  | Soya flour | Ts= ("soybean*" AND "flour*") OR Ts=("soya" AND "flour*") |
|  | Corn Soya blend | Ts=("maize*" AND "soya" AND "blend*") OR Ts=("zea mays" AND "soya" AND "blend*") OR Ts= ("corn" AND "soya" AND "blend*") OR Ts= ("maize*" AND "soybean*" AND "blend*") OR Ts=("zea mays" AND "soybean*" AND "blend*") OR Ts= ("corn" AND "soybean*" AND "blend*") OR Ts=”CSB” |
|  | Wheat soya blend | TS="WSB" OR TS = ("wheat" AND "soya" AND "blend*") OR TS = ("wheat" AND "soybean*" AND "blend*") |
|  | Nutritional rehabilitation | Ts= ("nutrition*" AND "rehabilit*") OR Ts=("nutritive" AND "rehabilit*") |
|  | Lipid-based nutrient supplements | Ts=("Lipid*" AND "nutrient*" AND "supplement*") |
|  | Nutrititional support | Ts=("nutrition*" AND "support") OR Ts=("nutrition*" AND "therapy") |
|  | Diet supplementation | Ts= ("diet*" AND "supplement*") |
|  | Fortified Food | Ts=("food*" AND "fortifi*") |
| Concept 3:  Nutritional status | Nutritional status | Ts=("nutrition*" AND "status") |
|  | Body composition | Ts=("body" AND "composition") |
|  | Body Weight gain/Body weight loss/underweight/wasting/undernutrition | Ts=("body" AND "weight" AND "gain") OR Ts=("body" AND "weight" AND "loss") OR Ts=("body" AND "weight" AND "decrease") OR Ts=("body" AND "weight" AND "reduction") OR Ts=("body" AND "weight" AND "increase") OR Ts=("body" AND "weight" AND "insufficiency") OR Ts= ("body" AND "thinness") OR Ts="underweight*" OR Ts= ("wasting" AND "syndrom*") OR Ts= "undernutrition*" |
|  | Lean mass/ Fat free mass | Ts=("lean" AND "mass") OR Ts=("fat" AND "free" AND "mass") |
|  | Micronutrient deficiencies | Ts= ("micronutrient*" AND "deficien*") OR Ts= ("micronutriment*" AND "deficien*") OR Ts=("trace" AND "element*" AND "deficien*") |
|  | Anorexia | Ts= "anorexi*" |
|  | Acute malnutrition | Ts=("acute*" AND "malnutrition*") OR Ts=("acute*" AND "malnourish*") |
|  | Body mass index | Ts=("body" AND "mass" AND "index") |
|  | Emaciation | Ts= "emaciat*" |
|  | Cachexia | Ts= "cachexia*" |
| Concept 4:  Adherence and response to ART | HIV drug side effects | Ts= ("anti retroviral*" AND "side" AND "effect*") OR Ts= ("antiretroviral*" AND "side" AND "effect*") OR Ts= ("arv" AND "side" AND "effect*") OR Ts= ("hiv" AND "drug" AND "side" AND "effect*") |
|  | Antiretroviral adherence | Ts= ("anti retroviral*" AND "adhere*") OR Ts= ("antiretroviral*" AND "adhere*") OR Ts= ("anti retroviral*" AND "adherance*") OR Ts=("antiretroviral*" AND "adherance*") OR Ts= ("arv" AND "adhere*") OR Ts= ("arv" AND "adherance*") OR Ts= ("hiv" AND "drug" AND "adhere*") OR Ts= ("hiv" AND "drug" AND "adherance*") |
|  | Viral load | Ts= ("viral" AND "load") |
|  | CD3 and CD4 | Ts="CD3" OR Ts="CD4" |
| Concept 5:  Low and middle income countries |  | Ts="afghan*” OR Ts="albania*” OR Ts="algeria*” OR Ts="american samoa*” OR Ts="angola*” OR Ts="antigua*” OR Ts="barbuda*” OR Ts="argentin*” OR Ts="armenia*” OR Ts="aruba*” OR Ts="azerbaijan*” OR Ts="bahrain*” OR Ts="bangladesh*” OR Ts="bangalees” OR Ts="barbados*” OR Ts="bajan*” OR Ts="belarus*” OR Ts="republic of Belarus” OR Ts="byelarus*” OR Ts="belorussia*” OR Ts="byelorussian*” OR Ts="belize*” OR Ts="british honduras*” OR Ts="benin*” OR Ts="dahomey*” OR Ts="bhutan*” OR Ts="bolivia*” OR Ts="Bosnia and Herzegovina”OR Ts="bosnia*” OR Ts="herzegovina*” OR Ts="botswana*” OR Ts="batswana*” OR Ts="bechuanaland*” OR Ts="brazil*” OR Ts="brasil*” OR Ts="bulgaria*” OR Ts="burkina fasso*”OR Ts="burkinabe*” OR Ts="burkinese*” OR Ts="upper volta*” OR Ts="burundi*” OR Ts="urundi*” OR Ts="cabo verde*” OR Ts="cape verde*” OR Ts="cambodia*” OR Ts="Kampuchea” OR Ts="khmer republic” OR Ts="khmer” OR Ts="cameroon*” OR Ts="Cameron” OR Ts="cameroun” OR Ts="central african republic” OR Ts="central african*” OR Ts="ubangi shari” OR Ts="chad*” OR Ts="chile” OR Ts="china” OR Ts="Chinese”OR Ts="colombia*” OR Ts="comoros” OR Ts="comoro islands” OR Ts="iles comores” OR Ts="comorian*” OR Ts="mayotte”OR Ts="democratic republic of the congo”OR Ts="congo*”OR Ts="zaire” OR Ts="costa rica*” OR Ts="cote d` Ivoire” OR Ts="cote d` Ivoire” OR Ts="cote divoire”OR Ts="cote d ivoire” OR Ts="ivory coast” OR Ts="ivorian*” OR Ts="crotia*” OR Ts="cuba*” OR Ts="cyprus” OR Ts="cypriot*” OR Ts="czech*” OR Ts="Czechoslovakia” OR Ts="djibouti*” OR Ts="french Somaliland” OR Ts="dominica*” OR Ts="ecuador*” OR Ts="egypt*” OR Ts="united arab republic” OR Ts="el Salvador” OR Ts="salvadoran*” OR Ts="equatorial guinea*” OR Ts="equatoguinean*”OR Ts="spanish guinea” OR Ts="eritrea*” OR Ts="estonia*”OR Ts="eswatini” OR Ts="Swaziland” OR Ts="swazi*” OR Ts="swati*” OR Ts="ethiopia*” OR Ts="fiji*” OR Ts="gabon*” OR Ts="gabonese republic” OR Ts="gambia*” OR Ts="georgia*” OR Ts="ghana*” OR Ts="gold coast” OR Ts="gibraltar*” OR Ts="Greece” OR Ts="greek*” OR Ts="grenada” OR Ts="grenadian*” OR Ts="guam*” OR Ts="guatemala*” OR Ts="guinea*” OR Ts="Guinea-Bissau” OR Ts="guinea Bissau” OR Ts="Guyana” OR Ts="Guyanese” OR Ts="haiti*” OR Ts="Hispaniola” OR Ts="Honduras” OR Ts="honduran*” OR Ts="hungary” OR Ts="hungarian*” OR Ts="india*” OR Ts="indonesia*” OR Ts="timor” OR Ts="iran*” OR Ts="iraq*” OR Ts="isle of man” OR Ts="manx” OR Ts="jamaica*” OR Ts="jordan*” OR Ts="kazakh*” OR Ts="kenya*” OR Ts="kirabati*” OR Ts="north korea*” OR Ts="democratic people` s republic of korea” OR Ts="republic of korea” OR Ts="south korea” OR Ts="korea*” OR Ts="kosovo”OR Ts="kosovar*” OR Ts="kosovan*” OR Ts="kyrgyzstan*” OR Ts="kirghizia” OR Ts="kirgizstan” OR Ts="kyrgyz republic” OR Ts="kirghiz” OR Ts="kyrgyz”OR Ts="laos” OR Ts="lao” OR Ts="laotian*” OR Ts="lao pdr” OR Ts="lao people` s democratic republic” OR Ts="latvia*” OR Ts="lebanon” OR Ts="lebanese republic” OR Ts="lebanese” OR Ts="lesotho*” OR Ts="lesothan*” OR Ts="mosotho*” OR Ts="basutoland”OR Ts="basotho” OR Ts="liberia*” OR Ts="libya*” OR Ts="lithuania*” OR Ts="macau*” OR Ts="macao*” OR Ts="macanese” OR Ts="macedonia*” OR Ts="madagascar” OR Ts="malagasy” OR Ts="madagascan*”OR Ts="malawi*” OR Ts="nyasaland” OR Ts="malaysia*” OR Ts="malay federation” OR Ts="malaya federation” OR Ts="maldives” OR Ts="maldivian” OR Ts="indian ocean islands” OR Ts="indian ocean” OR Ts="mali” OR Ts="malta”OR Ts="maltese” OR Ts="micronesia” OR Ts="kiribati” OR Ts="marshall islands” OR Ts="marshallese**” OR Ts="nauru*” OR Ts="northern mariana islands” OR Ts="palau” OR Ts="tuvalu” OR Ts="mauritania*” OR Ts="mauritius*” OR Ts="mauritian*”OR Ts="mexico” OR Ts="mexican*” OR Ts="moldova*” OR Ts="moldovian*” OR Ts="mongolia*” OR Ts="mongol” OR Ts="montenegro” OR Ts="montenegrin*” OR Ts="morocco” OR Ts="moroccan*” OR Ts="ifni” OR Ts="mozambique” OR Ts="mozambican*” OR Ts="portuguese east africa” OR Ts="myanma*” OR Ts="burma” OR Ts="burmese” OR Ts="namibia*” OR Ts="nauruan*” OR Ts="nepal*” OR Ts="netherlands antille*” OR Ts="nicaragua*” OR Ts="niger*” OR Ts="nigeria*” OR Ts="northern mariana inslander*” OR Ts="mariana*” OR Ts="oman*” OR Ts="muscat” OR Ts="pakistan*” OR Ts="panama*” OR Ts="papua new guinea*” OR Ts="new guinea*” OR Ts="palauan*” OR Ts="palnamian*” OR Ts="paraguay*” OR Ts="peru*” OR Ts="philippine*” OR Ts="philipine*” OR Ts="phillipine*” OR Ts="phillippine*” OR Ts="filipin*” OR Ts="poland*” OR Ts="polish people` s republic” OR Ts="polish” OR Ts="pole*” OR Ts="portugal*” OR Ts="portuguese republic” OR Ts="portuguese” OR Ts="puerto rico*” OR Ts="puerto rican*” OR Ts="romania*” OR Ts="russia*” OR Ts="ussr” OR Ts="soviet union” OR Ts="union of soviet socialist republics” OR Ts="soviet people” OR Ts="soviet population” OR Ts="rwanda*” OR Ts="Rwandese” OR Ts="ruanda*” OR Ts="ruandese” OR Ts="samoa*” OR Ts="pacific islands” OR Ts="polynesia*” OR Ts="navigator island*” OR Ts="sao tome and principe” OR Ts="sao tomean*” OR Ts="santomean*” OR Ts="'saudi arabia*” OR Ts="saudi*” OR Ts="senegal*” OR Ts="serbia*” OR Ts="seychell*” OR Ts="sierra leone*” OR Ts="slovakia*” OR Ts="slovak*” OR Ts="slovak republic” OR Ts="slovenia*” OR Ts="slovene*” OR Ts="melanesia*” OR Ts="solomon island*” OR Ts="norfolk island*” OR Ts="somali*” OR Ts="south africa*” OR Ts="south sudan*” OR Ts="sri lanka*” OR Ts="ceylon*” OR Ts="saint kitts and nevis” OR Ts="st. kitts and nevis” OR Ts="kittitian*” OR Ts="nevisian*” OR Ts="saint lucia*” OR Ts="st. lucia” OR Ts="saint vincent and the grenadines” OR Ts="saint Vincent” OR Ts="st. Vincent” OR Ts="grenadines” OR Ts="vincentian*” OR Ts="sudan*” OR Ts="surinam*” OR Ts="surinam*” OR Ts="dutch Guiana” OR Ts="netherlands Guiana” OR Ts="syria*” OR Ts="tajik*” OR Ts="tadjikistan*” OR Ts="Tadzhikistan” OR Ts="Tadzhik” OR Ts="tanzania*” OR Ts="tanganyika*” OR Ts="Thailand” OR Ts="siam” OR Ts="timor leste” OR Ts="timor-leste” OR Ts="timorese*” OR Ts="east timor” OR Ts="togo*” OR Ts="togolese republic” OR Ts="tonga*” OR Ts="trinidad andTobago” OR Ts="trinidad*” OR Ts="tobago*” OR Ts="tunisia*” OR Ts="turkey” OR Ts="turk*” OR Ts="Turkmenistan” OR Ts="turkmen*” OR Ts="tuvaluan*” OR Ts="uganda*” OR Ts="ukrain*” OR Ts="uruguay*” OR Ts="uzbek*” OR Ts="vanuatu*” OR Ts="new Hebrides” OR Ts="venezuela*” OR Ts="vietnam*” OR Ts="viet nam” OR Ts="middle east” OR Ts="west bank” OR Ts="gaze” OR Ts="Palestine” OR Ts="yemen*” OR Ts="yugoslav*” OR Ts="zambia*” OR Ts="zimbabwe*” OR Ts="northern rhodesia*” OR Ts="global south” OR Ts="africa south of the sahara” OR Ts="sub sahara Africa” OR Ts="subsaharan Africa” OR Ts="central Africa” OR Ts="africa, central” OR Ts="africa, northern” OR Ts="north Africa” OR Ts="northern Africa” OR Ts="magreb” OR Ts="maghrib” OR Ts="sahara” OR Ts="africa, southern” OR Ts="africa, southern” OR Ts="southern africa*” OR Ts="africa, eastern” OR Ts="east africa*” OR Ts="eastern africa*” OR Ts="africa, western” OR Ts="west africa*” OR Ts="western africa*” OR Ts="west indies” OR Ts="indian ocean islands” OR Ts="caribbean region” OR Ts="Caribbean” OR Ts="caribbean islands” OR Ts="central america*” OR Ts="south and central America” OR Ts="latin america*” OR Ts="south America” OR Ts="central asia*” OR Ts="northern asia*” OR Ts="north asia*” OR Ts="south eastern asia*” OR Ts="south eastern asia*” OR Ts="southeast asia*” OR Ts="south east asia*” OR Ts="western asia*” OR Ts="west asia*” OR Ts="eastern europe*” OR Ts="east europe*” OR Ts="developing countr*” OR Ts="developing nation*” OR Ts="developing population*” OR Ts="developing world” OR Ts="less developed countr*” OR Ts="less developed nation*” OR Ts="less developed population*” OR Ts="less developed world” OR Ts="lesser developed countr*” OR Ts="lesser developed nation*” OR Ts="lesser developed population*” OR Ts="lesser developed world” OR Ts="under developed countr*” OR Ts="under developed nation*” OR Ts="under developed population*” OR Ts="under developed world” OR Ts="underdeveloped countr*” OR Ts="underdeveloped nation*” OR Ts="underdeveloped population*” OR Ts="underdeveloped world” OR Ts="middle income countr*” OR Ts="middle income nation*” OR Ts="middle income population*” OR Ts="low income countr*” OR Ts="low income nation*” OR Ts="low income population*” OR Ts="lower income countr*” OR Ts="lower income nation*” OR Ts="lower income population*” OR Ts="underserved countr*” OR Ts="underserved nation*” OR Ts="underserved population*” OR Ts="underserved world” OR Ts="under served countr*” OR Ts="under served nation*” OR Ts="under served population*” OR Ts="under served world” OR Ts="deprived countr*” OR Ts="deprived nation*” OR Ts="deprived population*” OR Ts="deprived world” OR Ts="poor countr*” OR Ts="poor nation*” OR Ts="poor population*” OR Ts="poor world” OR Ts="poorer countr*” OR Ts="poorer nation*” OR Ts="poorer population*” OR Ts="poorer world” OR Ts="developing econom*” OR Ts="less developed econom*” OR Ts="lesser developed econom*” OR Ts="under developed econom*” OR Ts="underdeveloped econom*” OR Ts="middle income econom*” OR Ts="low income econom*” OR Ts="lower income econom*” OR Ts="low gdp” OR Ts="low gnp” OR Ts="low gross domestic” OR Ts="low gross national” OR Ts="lower gdp” OR Ts="lower gnp” OR Ts="lower gross domestic” OR Ts="lower gross national” OR Ts="lmic” OR Ts="lmics” OR Ts="third world” OR Ts="lami countr*” OR Ts="transitional countr*” OR Ts="emerging economies” OR Ts="emerging nation*” OR Ts="least developed countr*” OR Ts="low and middle income countr*" |
| Concept 6: study design | 6 | TS="clinical trial*" OR ts="randomized controlled trial*" OR ts="controlled clinical trial*" OR ts="control group stud*" OR ts="controlled study" OR ts="control group*" OR ts="random allocation" OR ts="Double-Blind" OR ts="Single-Blind" OR ts=double-masked OR ts=single-masked OR ts="crossover Stud*" OR ts="crossover trial*" OR ts="crossover procedure" OR ts="Placebo*" OR ts="multicenter study" OR ts="factorial design" OR ts="factorial trial*" OR ts= (repeated AND cross-sectional ) OR ts=trial* OR ts=rct* OR ts=random* OR ts="experimental stud*" OR ts="quasi experimental" OR ts="quasiexperimental stud*" |
