# Supplementary material for: Effects of nutritional interventions on nutritional and immunological status and adherence to antiretroviral treatment among adults living with HIV in low- and middle-income countries: Systematic review and meta-analysis
Source: PLoS One. 2025 Jun 3;20(6):e0319843. doi: 10.1371/journal.pone.0319843 (PMC12132990; doi:10.1371/journal.pone.0319843)
Supplement: S6 Table — (DOCX) [file pone.0319843.s007.docx]

## **S6 Table.** Characteristics of the studies excluded from the systematic review and meta-analyses.

| **Ref.** | **Author, title, year, journal title.** | **Reason for exclusion** |
| --- | --- | --- |
| [1] | Adu-Afarwuah et al. Randomized comparison of 3 types of micronutrient supplements for home fortification of complementary foods in Ghana: effects on growth and motor development. Am J Clin Nutr. 2007;86(2):412-20. | Ineligible study population, children |
| [2] | Actn. 2019. What is the effect of increasing dietary resistant starch on gut health and immunity in HIV-positive adults in India and is a feeding trial feasible? <https://www.cochranelibrary.com/central/doi/10.1002/central/CN-01970198/full> | Wrong design. Unpublished data |
| [3] | Ahoua et al. Nutrition outcomes of HIV-infected malnourished adults treated with ready-to-use therapeutic food in sub-Saharan Africa: a longitudinal study. J Int AIDS Soc. 2011;14:2. | Wrong study design. Longitudinal study. |
| [4] | Almeida et al. Impact of a nutritional counseling program on prevention of HAART-related metabolic and morphologic abnormalities. AIDS Care. 2011;23(6):755‐63. | Wrong intervention. The trial focused on Nutritional counselling. |
| [5] | Allard et al. Effects of vitamin E and C supplementation on oxidative stress and viral load in HIV-infected subjects. AIDS. 1998;12(13):1653-9. | Ineligible study populations. Wrong location |
| [6] | Alo et al. Effects of nutrition counseling and monitoring on the weight and hemoglobin of patients receiving antiretroviral therapy in Eebonyi State, Southeast Nigeria. Research and Palliative Care. 2014;6:91-7. | The trial focused on nutritional counselling |
| [7] | Amador-Licona et al. Omega 3 Fatty Acids supplementation and  Oxidative Stress in HIV-Seropositive Patients.  A Clinical Trial. PLoS One. 2016;11(3):e0151637. | Wrong intervention .The trial focused on the reduction of oxidative stress |
| [8] | Ammassari et al. Self-reported symptoms and medication side effects influence adherence to highly active antiretroviral therapy in persons with HIV infection. J Acquir Immune Defic Syndr. 2001;28(5):445-9. | Ineligible study populations. Wrong location |
| [9] | Anabwani et al. Nutrition and HIV/AIDS in sub-Saharan Africa: an overview. Nutrition. 2005;21(1):96-9. | Wrong study design. Wrong publication type. |
| [10] | Andersen et al. Effects on mortality of a nutritional intervention  for malnourished HIV-infected adults referred for antiretroviral therapy: a randomized controlled trial. BMC Med. 2015;13(1). | Wrong comparator. The comparator received supplementation. |
| [11] | Anukam et al. Yogurt Containing Probiotic Lactobacillus rhamnosus GR-1 and L. reuteri RC-14 Helps Resolve Moderate Diarrhea and Increases CD4 Count in HIV/AIDS Patients. J Clin Gastroenterol. 2008;42(3):239-43. | Ineligible study populations. Patients were not on ARV treatment |
| [12] | Arsenault et al. Vitamin Supplementation Increases Risk of Subclinical Mastitis in HIV-Infected Women. J Nutr. 2010;140(10):1788-92. | Ineligible study populations. Lactating women |
| [13] | Asdamongkol et al. Low Plasma Zinc Levels and Immunological Responses to Zinc Supplementation in HIV-Infected Patients with Immunological Discordance after Antiretroviral Therapy. Jpn J Infect Dis. 2013;66(6):469-74. | Ineligible study populations. Patients with coinfection (Tuberculosis) |
| [14] | Ashenafi et al. Daily Nutritional Supplementation with Vitamin D3 and Phenylbutyrate to Treatment-Naïve HIV Patients Tested in a Randomized Placebo-Controlled Trial. Nutrients. 2019;11(1). | Ineligible study populations. Patients were not on ARV treatment. |
| [15] | Audain et al. Food supplementation among HIV-infected adults in Sub-Saharan Africa: Impact on treatment adherence and weight gain. Proc Nutr Soc. 2015;74(4):517-25. | Wrong study design. It was a systematic review |
| [16] | Ayuba et al. Clinical efficacy of a West African sorghum bicolor-based traditional herbal preparation Jobelyn shows increased hemoglobin and CD4+ T-lymphocyte counts in HIV-positive patients. J Altern Complement Med. 2014;20(1):53‐6. | Wrong intervention using traditional drugs. |
| [17] | Azabji-Kenfack et al. Potential of Spirulina Platensis as a Nutritional Supplement in Malnourished HIV-Infected Adults in Sub-Saharan Africa: A Randomised, Single-Blind Study. Nutr Metab Insights. 2011;4:29-37. | The trial has no control group |
| [18] | Badiane et al. Increasing energy and zinc intakes to level recommended by who improve fat-free mass but not zinc status in HIV/AIDS people. Ann Nutr Metab. 2013;63:204. | Ineligible study populations. Hospitalized population. |
| [19] | Baeten et al. Vitamin A Supplementation and Human Immunodeficiency Virus Type 1 Shedding in Women: Results of a Randomized Clinical Trial. J Infect Dis. 2002;185(8):1187-91. | Ineligible study populations. Patients were not on ARV treatment |
| [20] | Baingana et al. Effect of food supplementation on body composition of people with HIV/AIDS in Uganda. Ann Nutr Metab. 2009;55:260-. | Ineligible study populations. Patients were not on ARV treatment. |
| [21] | Bakeine et al. The effect of early nutritional supplementation with Nutrifil or Corn Soya Blend on the nutritional and immune status of adults with HIV infection in Uganda. Proceedings of the nutritional society. 1997;56(3):282A. | Ineligible study populations. Patients were not on ARV treatment |
| [22] | Black et al. Iron and zinc supplementation promote motor development and exploratory behavior among Bangladeshi infants. AJCN. 2004;80(4):903-10. | Ineligible study populations, children |
| [23] | Bang et al. The effect of cholecalciferol and calcitriol on biochemical bone markers in HIV type 1-infected males: results of a clinical trial. AIDS Res Hum Retroviruses. 2013;29(4):658-64. | Ineligible study populations. Wrong location |
| [24] | Bang et al. Correlation of increases in 1,25-dihydroxyvitamin D during vitamin D therapy with activation of CD4+ T lymphocytes in HIV-1-infected males. HIV Clin Trials. 2012;13(3):162-70. | Ineligible study populations. Wrong location |
| [25] | Bationo et al. Impact of spirulina on the evolution of the anthropometric, biochemical and hematological parameters of HIV-infected adults in Ouagadougou, Burkina Faso. Ann Nutr Metab. 2013;63:444. | Wrong design. Study protocol unpublished |
| [26] | Batterham et al. A comparison of megestrol acetate, nandrolone decanoate and dietary counselling for HIV associated weight loss. Int J Androl. 2001;24(4):232-40. | Wrong intervention. Drug intervention |
| [27] | Baum et al. Randomized, controlled clinical trial of zinc supplementation to prevent immunological failure in HIV-infected adults. Clin Infect Dis. 2010;50(12):1653-60. | Ineligible study populations. Wrong location |
| [28] | Baum et al. Effect of micronutrient supplementation on disease progression in asymptomatic, antiretroviral-naive, HIV-infected adults in Botswana: A randomized clinical trial. JAMA. 2013;310(20):2154-63. | Ineligible study populations. Patients were not on ARV treatment. |
| [29] | Beckett et al. Acceptability and use of ready-to-use supplementary food compared to corn–soy blend as a targeted ration in an HIV program in rural Haiti: a qualitative study. AIDS Res Ther. 2016;13:11. | Wrong study. Qualitative study to evaluate the acceptability and use of peanut-based RUSF com-pared to corn–soy blend (CSB) among adults living with HIV |
| [30] | Berneis et al. Nutritional supplements combined with dietary counselling diminish whole body protein catabolism in HIV-infected patients. Eur J Clin Invest. 2000;30(1):87-94. | Ineligible study populations. Wrong location |
| [31] | Bhima et al. Tackling protein–energy under-nutrition among  resource-limited people living with HIV/AIDS in Malawi using soybean-enriched maize-based stiff porridge (nsima): A pilot study. Nutr Diet. 2019;76(3):257-62. | Wrong design. Cohort study |
| [32] | Boontanondha et al. Vitamin D and Calcium Supplement Attenuate Bone Loss among HIV Infected Patients Receiving Tenofovir Disoproxil Fumarate/Emtricitabine/ Efavirenz: An Open-Label, Randomized Controlled Trial. Current HIV research. 2020;18(1):52-62. | Wrong intervention, drug |
| [33] | Burbano et al. Impact of a selenium chemoprevention clinical trial on hospital admissions of HIV-infected participants. HIV Clin Trials. 2002;3(6):483-91. | Ineligible study populations. Wrong location |
| [34] | Bushen et al. Diarrhea and Reduced Levels of Antiretroviral Drugs: Improvement with Glutamine or Alanyl-Glutamine in a Randomized Controlled Trial in Northeast Brazil. Clin Infect Dis. 2004;38(12):1764-70. | Ineligible study populations. The adults in HIV-positive with diarrhea. |
| [35] | Cárcamo et al. Randomized Controlled Trial of Zinc Supplementation for Persistent Diarrhea in Adults With HIV-1 Infection. J Acquir Immune Defic Syndr. 2006;43(2):197‐201. | Coinfection: diarrhea |
| [36] | Chotivichien et al. Effect of nutritional counseling on low-density lipoprotein cholesterol among Thai HIV-infected adults receiving antiretroviral therapy. AIDS Care. 2016;28(2):257-65. | Wrong intervention. The study focused on behavioral change |
| [37] | Clark et al. Nutritional treatment for acquired immunodeficiency virus-associated wasting using beta-hydroxy beta-methyl butyrate, glutamine, and arginine: a randomized, double-blind, placebo-controlled study. JPEN. 2000;24(3):133-9. | Ineligible study populations. Wrong location |
| [38] | Coates et al. Persistence of recovery from malnutrition and HIV progression among HIV+ adults graduating from the Ethiopia food by prescription program. Ann Nutr Metab. 2013;63:527. | Wrong design. Program evaluation studies |
| [39] | Coelho et al. Vitamin D3 supplementation in HIV infection: effectiveness and associations with antiretroviral therapy. Nutr J. 2015;14:81. | Wrong study design |
| [40] | Coghill et al. Omega-3 decreases IL-6 levels in HIV and human herpesvirus-8 coinfected patients in Uganda. AIDS. 2018;32(4):505-12. | Ineligible study populations. Patients with coinfection ( human herpesvirus-8) |
| [41] | Coodley et al. Beta-carotene in HIV infection: an extended evaluation. AIDS. 1996;10(9):967-73. | Ineligible study populations. Wrong location |
| [42] | Coodley et al. Beta-carotene in HIV infection. J Acquir Immune Defic Syndr. 1993;6(3):272-6. | Ineligible study populations. Wrong location |
| [43] | de Luis et al. Isolated dietary counselling program versus supplement and dietary counselling in patients with human immunodeficiency virus infection. Med Clin (Barc). 2003;120(15):565-7. | Ineligible study populations. Wrong location |
| [44] | De Pee et al. Role of nutrition in HIV infection: review of evidence for more effective programming in resource-limited settings. Food Nutr Bull. 2010;31(4):S313-44. | Wrong study design. Wrong publication type. |
| [45] | Derose et al. A pre-post pilot study of peer nutritional counseling and food insecurity and nutritional outcomes among antiretroviral therapy patients in Honduras. BMC nutrition. 2015;1. | Wrong design. The study focused on behavioral change |
| [46] | Diouf et al. Daily consumption of ready-to-use peanut-based therapeutic food increased fat free mass, improved anemic status but has no impact on the zinc status of people living with HIV/AIDS: a randomized controlled trial. BMC Public Health. 2016;16:1. | Wrong intervention. The adults were hospitalized. |
| [47] | Dougherty et al. Safety and Efficacy of High-Dose Daily Vitamin D3 Supplementation in Children and Young Adults Infected With Human Immunodeficiency Virus. Journal of the Pediatric Infectious Diseases Society. 2014;3(4):294-303. | Ineligible study populations: children |
| [48] | Ekstr et al. A Behavioral Adherence Intervention Improves Rates of Viral Suppression Among Adherence‑Challenged People Living with HIV in South India. AIDS Behav. 2020;24(7):2195-205. | Wrong intervention. The study focused on behavioral change |
| [49] | Ernst et al. High nutrition biscuits as a supplement to increase animal protein in diets of HIV-infected Kenyan women and their children. Ann Nutr Metab. 2013;63:144. | Wrong study design. Study protocol unpublished. |
| [50] | Etminani-Esfahani et al. Effects of vitamin D supplementation on the bone specific biomarkers in HIV infected individuals under treatment with efavirenz. BMC Res Notes. 2012;5:204. | Wrong outcome. The trial focused on bone-specific biomarkers |
| [51] | Faber et al. Effect of a fortified maize-meal porridge on anemia, micronutrient status, and motor development of infants. Am J Clin Nutr. 2005;82(5):1032-9. | Ineligible study populations: children |
| [52] | Fawzi et al. Multivitamin supplementation improves hematologic status in HIV-infected women and their children in Tanzania. Am J Clin Nutr. 2007;85(5):1335-43. | Ineligible study populations. Pregnant women |
| [53] | Fawzi et al. A Randomized Trial of Multivitamin Supplements and HIV Disease Progression and Mortality. N Engl J Med. 2004;351(1):23-32. | Ineligible study populations. Pregnant women |
| [54] | Ferreira et al. Effectiveness of a bioactive food compound in anthropometric measures of individuals with HIV/AIDS: A nonrandomized trial. PLoS One. 2018;13(2). | The trial focused on the reduction of oxidative stress |
| [55] | Flax et al. Plasma Micronutrient Concentrations Are Altered by Antiretroviral Therapy and Lipid- Based Nutrient Supplements in Lactating HIV- Infected Malawian Women. J Nutr. 2015;145(8):1950-7. | Ineligible study populations. Lactating women |
| [56] | Freiberg et al. Effect of Zinc Supplementation vs Placebo on Mortality Risk and HIV Disease Progression Among HIV-Positive Adults With Heavy Alcohol Use A Randomized Clinical Trial. JAMA Network Open. 2020. | The adults in HIV-positive with heavy alcohol. |
| [57] | Ghosh et al. Factors associated with recovery among Ethiopian malnourished HIV patients (pre art and art) that received food by prescription. Ann Nutr Metab. 2013;63:816. | Wrong study design. It was a program evaluation studies |
| [58] | Giacomet et al. Cholecalciferol supplementation in HIV-infected youth with vitamin D insufficiency: effects on vitamin D status and T-cell phenotype: a randomized controlled trial. HIV Clin Trials. 2013;14(2):51-60. | Ineligible study populations. Wrong location |
| [59] | Gnatienko et al. Design of a Randomized Controlled Trial of Zinc Supplementation to Improve Markers of Mortality and HIV Disease Progression in HIV-positive Drinkers in St. Petersburg, Russia. HIV Clin Trials. 2018;19(3):101-11. | Ineligible study populations. The adults in HIV-positive with heavy alcohol. |
| [60] | Goncalves et al. Iron intake is positively associated with viral load in antiretroviral naive Brazilian men living with HIV. Mem Inst Oswaldo Cruz. 2019;114. | Ineligible study populations. Patients were not on ARV treatment |
| [61] | González-Hernández et al. Synbiotic therapy decreases microbial translocation and inflammation and improves immunological status in HIV-infected patients: a double-blind randomized controlled pilot trial. Nutr J. 2012;11:90. | Ineligible study populations. Patients were not on ARV treatment |
| [62] | Green et al. A randomised controlled trial of oral zinc on the immune response to tuberculosis in HIV-infected patients. The international journal of tuberculosis and lung disease. 2005;9(12):1378-84. | Ineligible study populations. Patients with coinfection (Tuberculosis) |
| [63] | Grigoletti et al. Short-term folinic acid supplementation improves vascular reactivity in HIV-infected individuals: A randomized trial. Nutrition. 2013;29(6):886‐91. | Wrong intervention. Traditional drugs |
| [64] | Grobler et al. Nutritional supplements for people being treated for active tuberculosis. The Cochrane database of systematic reviews. 2016;2016(6):Cd006086. | Ineligible study populations. Patients with coinfection (Tuberculosis) |
| [65] | Haider et al. Anemia, iron deficiency, and iron supplementation in relation to mortality among HIV-infected patients receiving highly active antiretroviral therapy in Tanzania. Am J Trop Med Hyg. 2019;100(6):1512-20. | Wrong study design because it is the cohort study. |
| [66] | Hardon et al. Hunger, waiting time and transport costs: time to confront challenges to ART adherence in Africa. AIDS Care. 2007;19(5):658-65. | Wrong study design. Qualitative study |
| [67] | Higgins et al. Quantifying heterogeneity in a meta-analysis. Stat Med. 2002;21(11):1539-58. | Wrong study design. Wrong publication type. |
| [68] | Humphrey et al. Short-term effects of large-dose vitamin A supplementation on viral load and immune response in HIV-infected women. J Acquir Immune Defic Syndr Hum Retrovirol. 1999;20(1):44-51. | Ineligible study populations. Wrong location |
| [69] | Hurwitz et al. Suppression of human immunodeficiency virus type 1 viral load with selenium supplementation: a randomized controlled trial. Arch Intern Med. 2007;167(2):148-54. | Ineligible study populations. Wrong location |
| [70] | Irvine et al. Probiotic yogurt consumption may improve gastrointestinal symptoms, productivity, and nutritional intake of people living with human immunodeficiency virus in Mwanza, Tanzania. Nutr Res. 2011;31(12):875-81. | Wrong design. An observational retrospective study |
| [71] | Irvine et al. Probiotic yogurt consumption is associated with an increase of CD4 count among people living with HIV/AIDS. J Clin Gastroenterol. 2010;44(9):e201-e5. | Wrong design. An observational retrospective study |
| [72] | Isabirye et al. Dietary Micronutrients and Gender, Body Mass Index and Viral Suppression Among HIV-Infected Patients in Kampala, Uganda. IJMA. 2020;9(3):337-49. | Wrong study design, survey study |
| [73] | Isanaka et al. Standard-dose vs high-dose multivitamin supplements for HIV—reply. JAMA. 2013;309(6):546. | Wrong study design. Wrong publication. |
| [74] | Isanaka et al. Effect of high-dose vs standard-dose multivitamin supplementation at the initiation of HAART on HIV disease progression and mortality in Tanzania: A randomized controlled trial. JAMA. 2012;308(15):1535-44. | The trial has no control group |
| [75] | Isrctn. 2005. A nutritional supplement for human immunodeficiency virus (HIV) antibody positive patients at Mengo Hospital, Kampala, Uganda. <http://wwwwhoint/trialsearch/Trial2aspx?TrialID=ISRCTN42274642> | Wrong design. Unpublished data |
| [76] | Isrctn. 2007. Impact of Spirulina platensis supplementation on general health status of HIV infected patients in Burkina Faso. <http://wwwwhoint/trialsearch/Trial2aspx?TrialID=ISRCTN83770226>. | Wrong design. Unpublished data |
| [77] | Ivers et al. A Randomized Trial of Ready-to-Use Supplementary Food Versus Corn-Soy Blend Plus as Food Rations for HIV-Infected Adults on Antiretroviral Therapy in Rural Haiti. Clin Infect Dis. 2014;58(8):1176-84. | Wrong comparator .The trial has no control group |
| [78] | James et al. Minimal impact of an iron-fortified lipid-based nutrient supplement on Hb and iron status: a randomised controlled trial in malnourished HIV-positive African adults starting antiretroviral therapy. Br J Nutr. 2015;114(3):387-97. | Ineligible study populations. Patients were not on ARV treatment |
| [79] | Ji et al. A planting and eating soybean project for people living with HIV/AIDS in rural Anhui - A pilot study in China. AIDS Care. 2010;22(1):126-32. | Wrong outcome. Study on a soya plantation |
| [80] | Jiamto et al. A randomized placebo-controlled trial of the impact of multiple micronutrient supplementation on HIV-1 genital shedding among Thai subjects | Ineligible study populations. Patients were not on ARV treatment |
| [81] | Jiamton et al. A randomized trial of the impact of multiple micronutrient supplementation on mortality among HIV-infected individuals living in Bangkok. AIDS. 2003;17(17):2461-9. | Ineligible study populations. Patients were not on ARV treatment |
| [82] | Kabagambe et al. Plasma n-6 fatty acid levels are associated with CD4 cell counts, hospitalization, and mortality in HIV-infected patients. JAIDS. 2016;73(5):598-605. | Wrong design |
| [83] | Kaducu et al. Effect of bovine colostrum-based food supplement in the treatment of HIV-associated diarrhea in Northern Uganda: a randomized controlled trial. Indian J Gastroenterol. 2011;30(6):270-6. | Ineligible study populations. The adults in HIV-positive with diarrhea. |
| [84] | Kaiser et al. Micronutrient supplementation increases CD4 count in HIV-infected individuals on highly active antiretroviral therapy: a prospective, double-blinded, placebo-controlled trial. J Acquir Immune Defic Syndr. 2006;42(5):523-8. | Ineligible study populations. Wrong location |
| [85] | Kamwesiga et al. Effect of selenium supplementation on CD4R T-cell recovery, viral suppression and morbidity of HIV infected patients in Rwanda: A randomized controlled trial. AIDS. 2015;29(9):1045-52. | Ineligible study populations. Patients were not on ARV treatment. |
| [86] | Kamwesiga et al. Effect of selenium supplementation on CD4 depletion in Rwandan HIV patients: A randomized trial. Top Antivir Med. 2014;22:267-8. | Ineligible study populations. Patients were not on ARV treatment. |
| [87] | Karsegard et al. L-ornithine alpha-ketoglutarate in HIV infection: effects on muscle, gastrointestinal, and immune functions. Nutrition. 2004;20(6):515-20. | Ineligible study populations. Wrong location |
| [88] | Keithley et al. Comparison of standard and immune-enhancing oral formulas in asymptomatic HIV-infected persons: a multicenter randomized controlled clinical trial. JPEN. 2002;26(1):6-14. | Ineligible study populations. Wrong location |
| [89] | Kelly et al. Micronutrient supplementation has limited effects on intestinal infectious disease and mortality in a Zambian population of mixed HIV status: a cluster randomized trial. Am J Clin Nutr. 2008;88(4):1010-7. | Wrong outcome. The trial focused on Gastric hypochlorhydria and intestinal barrier dysfunction in HIV infection is not dependent on nutrition |
| [90] | Kelly et al. Micronutrient supplementation in the AIDS diarrhoea-wasting syndrome in Zambia: a randomized controlled trial. AIDS. 1999;13(4):495-500. | Wrong outcome. The trial focused on gastric and intestinal barrier impairment in tropical enteropathy and HIV |
| [91] | Kelly et al. Gastric hypochlorhydria and intestinal barrier dysfunction in HIV infection is not dependent on nutrition: A randomised controlled trial of supplementation. Gut. 2009;58:A82-A3. | Ineligible study populations. The adults in HIV-positive with diarrhea. |
| [92] | Kelly et al. Gastric and intestinal barrier impairment in tropical enteropathy and HIV: Limited impact of micronutrient supplementation during a randomised controlled trial. BMC Gastroenterol. 2010;10. | Ineligible study populations. The adults in HIV-positive with diarrhea. |
| [93] | Kotler. Malnutrition in HIV infection and AIDS. AIDS. 1989;3 Suppl 1:S175-80. | Wrong study design. Wrong publication type. |
| [94] | Kotler et al. Studies of body composition and fat distribution in HIV-infected and control subjects. J Acquir Immune Defic Syndr Hum Retrovirol. 1999;20(3):228-37. | Ineligible study populations. Wrong location |
| [95] | Lebouché et al. Impact of extended-release niacin on immune activation in HIV-infected immunological non-responders on effective antiretroviral therapy. | Ineligible study populations. Wrong location |
| [96] | Macallan et al. Sir David Cuthbertson Prize Medal Lecture. Metabolic abnormalities and wasting in human immunodeficiency virus infection. The Proceedings of the Nutrition Society. 1998;57(3):373-80. | Wrong study design. Wrong publication type. |
| [97] | Macallan et al. Whole-body protein turnover from leucine kinetics and the response to nutrition in human immunodeficiency virus infection. AJCN. 1995;61(4):818-26. | Wrong study design. Cohort study |
| [98] | Macallan et al. Prospective analysis of patterns of weight change in stage IV human immunodeficiency virus infection. 1993;58(3):417-24. | Wrong study design. Cohort study |
| [99] | Macallan et al. Energy expenditure and wasting in human immunodeficiency virus infection. The New England journal of medicine. 1995;333(2):83-8. | Wrong study design. Cohort study |
| [100] | Mallewa et al. A randomized trial of ready-to-use supplementary food at art initiation in Africa. Top Antivir Med. 2017;25(1):45s-6s. | Wrong comparator. The comparator received supplementation. |
| [101] | Mallewa et al. Effect of ready-to-use supplementary food on mortality in severely immunocompromised HIV-infected individuals in Africa initiating antiretroviral therapy (REALITY): an open-label, parallel-group, randomised controlled trial. The Lancet HIV. 2018;5(5):e231-e40. | Ineligible study populations. The trial involved children |
| [102] | Maluccio et al. Improving Health-Related Quality of Life among People Living with HIV: Results from an Impact Evaluation of a Food Assistance Program in Uganda. PLoS One. 2015;10(8):e0135879. | Ineligible study populations. Patients were not on ARV treatment. |
| [103] | Manary et al. Supplementary feeding in the care of the wasted HIV infected patient. Malawi Med J. 2010;22(2):46-9. | Wrong comparator. The trial has no control group. |
| [104] | Mansouri et al. Comparative study of levamisole-selenium supplementation effect on CD4 increase in HlV/ AIDS patients. Caspian Journal of Internal Medicine. 2011;2(2):218-21. | Ineligible study populations. Patients were not on ARV treatment. |
| [105] | Marazzi et al. Excessive early mortality in the first year of treatment in HIV type 1-infected patients initiating antiretroviral therapy in resource-limited settings. AIDS Res Hum Retroviruses. 2008;24(4):555-60. | Wrong study design. Retrospective cohort |
| [106] | Marcel et al. The Effect of Spirulina platensis versus Soybean on Insulin Resistance in HIV-Infected Patients: A Randomized Pilot Study. Nutrients. 2011;3(7):712-24. | Wrong comparator. The trial has no control group. |
| [107] | Martí-Carvajal et al. Pharmacological interventions for treating dyslipidemia in patients with HIV infection. Cochrane Database Syst Rev. 2018;2018(2). | Wrong intervention. Drug. |
| [108] | Mburu et al. The influence of inflammation on plasma zinc concentration in apparently healthy, HIV+ Kenyan adults and zinc responses after a multi micronutrient supplement. Eur J Clin Nutr. 2010;64(5):510‐7. | Ineligible study populations. Patients were not on ARV treatment. |
| [109] | Mburu et al. The influence and benefits of controlling for inflammation on plasma ferritin and hemoglobin responses following a multimicronutrient supplement in apparently healthy, HIV+ Kenyan adults. J Nutr. 2008;138(3):613-9. | Ineligible study populations. Patients were not on ARV treatment |
| [110] | McClell et al. Micronutrient supplementation increases genital tract shedding of HIV-1 in women: Results of a randomized trial. JAIDS. 2004;37(5):1657-63. | Ineligible study populations. Patients were not on ARV treatment |
| [111] | McCoy et al. A randomized study of short-term conditional cash and food assistance to improve adherence to antiretroviral therapy among food insecure adults with HIV infection in Tanzania. J Int AIDS Soc. 2016;19:88. | Wrong outcome. Study focused on food insecurity |
| [112] | McGrath et al. Effect of maternal multivitamin supplementation on the mental and psychomotor development of children who are born to HIV-1-infected mothers in Tanzania. Pediatrics. 2006;117(2):e216-25. | Ineligible study populations. Wrong location |
| [113] | Mensah et al. An evaluation of a community-based food supplementation for people living with HIV in Ghana: implications for community-based interventions in Ghana. BMC Res Notes. 2015;8:519. | Wrong study design. It was a survey study |
| [114] | Moore et al. Selenium supplementation in HIV-infected individuals: A systematic review of randomized controlled trials. J Nutr Biochem. 2000;11(6):341-7. | Wrong design. Program evaluation |
| [115] | Motswagole et al. The Efficacy of Micronutrient-Fortified Sorghum Meal in Improving the Immune Status of HIV-Positive Adults. Ann Nutr Metab. 2013;62(4):323-30. | Wrong comparator. The comparator received supplementation. |
| [116] | Munkombwe et al. Lipid-based nutrient supplements containing vitamins and minerals attenuate renal electrolyte loss in HIV/AIDS patients starting antiretroviral therapy: A randomized controlled trial in Zambia. Clinical Nutrition ESPEN. 2016;13:e8-e14. | Wrong outcome. The study focused on renal electrolyte loss nutritional status. |
| [117] | Namulema et al. When the Nutritional Supplements Stop: Evidence from a Double-blinded, HIV Clinical Trial at Mengo Hospital, Kampala, Uganda. Journal of orthomolecular medicine. 2008;23(3):130‐2. | Ineligible study populations. Patients were not on ARV treatment. |
| [118] | Namulemia et al. Nutritional Supplements Can Delay the Progression of AIDS in HIV-Infected Patients: Results from a Double-Blinded, Clinical Trial at Mengo Hospital, Kampala, Uganda. Journal of Orthomolecular Medicine. 2007;22(3):129-36. | Ineligible study populations. Patients were not on ARV treatment. |
| [119] | Nct, 2009. Study of Impacts of Food Supplementation on Malnourished HIV-Infected Adults in Kenya. <https://clinicaltrialsgov/show/NCT00838292> | It is a study protocol, unpublished data. |
| [120] | Nct, 2018. The Role of Probiotics in HIV Patients With Immunological Non-Responder. <https://clinicaltrialsgov/show/NCT03568812> | Wrong study design. It is a study protocol unpublished. |
| [121] | Nct, 2010. "Arthrospira Platensis" as Nutrition Supplementation for Female Adult Patients Infected by HIV in Yaoundé Cameroon. <https://clinicaltrialsgov/show/NCT01084382> | Wrong study design. It is a study protocol unpublished. |
| [122] | Ndekha et al. Supplementary feeding with either ready-to-use fortified spread or corn-soy blend in wasted adults starting antiretroviral therapy in Malawi: randomised, investigator blinded, controlled trial. BMJ (Online). 2009;338(7706):1309-11. | Wrong comparator. The trial has no control group. |
| [123] | Ndekha et al. Nutritional status of Malawian adults on antiretroviral therapy 1 year after supplementary feeding in the first 3 months of therapy. Trop Med Int Health. 2009;14(9):1059-63. | Wrong comparator. The trial has no control group |
| [124] | Ngo-Matip et al. Effects of Spirulina platensis supplementation on lipid profile in HIV–infected antiretroviral naïve patients in Yaoundé - Cameroon: a randomized trial study. Lipids Health Dis. 2014;13:191. | Ineligible study populations. Patients were not on ARV treatment |
| [125] | Ngo-Matip et al. Impact of daily supplementation of Spirulina Platensis on the immune system of naïve HIV-1 patients in Cameroon: a 12-months single blind, randomized, multicenter trial. Nutr J. 2015;14(1). | Ineligible study populations. Patients were not on ARV treatment |
| [126] | Odunukwe et al. Selenium as adjunct to HAART in the management of HIV/hepatitis b virus coinfection: a randomized open label study. African Journal of Clinical and Experimental Microbiology. 2016;17(3):197-204. | Ineligible study populations. Patients with coinfection (HIV/hepatitis b virus coinfection) |
| [127] | Ogbuagu et al. CD4 pattern in HIV positive patients on HAART exposed to moringa oleifera leaf powder in south east Nigeria. Int J Infect Dis. 2016;45:267. | Abstract in a conference proceeding. No full text published. |
| [128] | Oguntibeju et al. Supplementation effect on Body Weight and BMI of HIV-positive/AIDS patients. International Journal of Pharmacology. 2007;3(1):120-2. | Wrong design. Cohort study |
| [129] | Oluwafemi et al. A locally produced nutritional supplement in community-based HIV and AIDS patients. | Ineligible study populations. Patients were not on ARV treatment |
| [130] | Oketch et al. Too little, too late: Comparison of nutritional status and quality of life of nutrition care and support recipient and non-recipients among HIV-positive adults in KwaZulu-Natal, South Africa. Int J Palliat Nurs. 2007;13(4):154-62. | Wrong design. Program evaluation |
| [131] | Oliveira et al. Effects of a Low Dose of Fish Oil on Inflammatory Markers of Brazilian HIV-Infected Adults on Antiretroviral Therapy: A Randomized, Parallel, Placebo-Controlled Trial. Nutrients. 2015;7(8):6520-8. | Wrong intervention .The trial focused on the reduction of oxidative stress |
| [132] | Oliveira et al. Effects of fish oil on lipid profile and other metabolic outcomes in HIV-infected patients on antiretroviral therapy: a randomized placebo-controlled trial. Int J STD AIDS. 2014;25(2):96-104. | Wrong intervention .The trial focused on the reduction of oxidative stress |
| [133] | Olofin et al. Supplementation With Multivitamins and Vitamin A and Incidence of Malaria Among HIV-Infected Tanzanian Women. J Acquir Immune Defic Syndr. 2014;67:S173-S8. | Ineligible study populations. Pregnant women |
| [134] | Overton et al. Vitamin D and Calcium Attenuate Bone Loss With Antiretroviral Therapy Initiation: A Randomized Trial. Ann Intern Med. 2015;162(12):815-24. | Ineligible study populations. Wrong location |
| [135] | Palar et al. Impact of food support on food security and body weight among HIV antiretroviral therapy recipients in Honduras: a pilot intervention trial. AIDS Care. 2015;27(4):409-15. | Wrong outcome. The trial focused on oxidative stress |
| [136] | Peters et al. The Effect of a 12-Week Course of Omega-3 Polyunsaturated Fatty Acids on Lipid Parameters in Hyper triglyceridemic Adult HIV-infected Patients Undergoing HAART: A Randomized, Placebo-Controlled Pilot Trial. Clin Ther. 2012;34(1):67‐76. | Wrong population; inadequate setting (trials in HICs) |
| [137] | Petrilli et al. Effect of Chocolate and Yerba Mate Phenolic Compounds on Inflammatory and Oxidative Biomarkers in HIV/AIDS Individuals. Nutrients. 2016;8(5). | Wrong intervention .The trial focused on the reduction of oxidative stress |
| [138] | Praygod et al. Persistent inflammation on ART is associated with poor nutritional recovery in Zambia. Top Antivir Med. 2015;23:342. | Wrong comparator. The comparator received supplementation. |
| [139] | Praygod et al. Effects on body composition and handgrip strength of a nutritional intervention for malnourished HIV-infected adults referred for antiretroviral therapy: a randomised controlled trial. Journal of nutritional science. 2019;8:e19. | Wrong comparator. The comparator received supplementation. |
| [140] | Rabeneck et al. A randomized controlled trial evaluating nutrition counseling with or without oral supplementation in malnourished HIV-infected patients. J Am Diet Assoc. 1998;98(4):434-8. | Ineligible study populations. Wrong location |
| [141] | Rawat et al. The Impact of a Food Assistance Program on Nutritional Status, Disease Progression, and Food Security Among People Living With HIV in Uganda. JAIDS. 2014;66(1):e15-22. | Ineligible study populations. Patients were not on ARV treatment |
| [142] | Rawat et al. The impact of food assistance on weight gain and disease progression among HIV-infected individuals accessing AIDS care and treatment services in Uganda. BMC Public Health. 2010;10:316. | Wrong study design. Program evaluation. |
| [143] | Rehman et al. Effects on Anthropometry and Appetite of Vitamins and Minerals Given in Lipid Nutritional Supplements for Malnourished HIV-Infected Adults Referred for Antiretroviral Therapy: Results From the NUSTART Randomized Controlled Trial. J Acquir Immune Defic Syndr. 2015;68(4):405-12. | Wrong comparator. The comparator received supplementation. |
| [144] | Rothman et al. The Impact of a Community-Based Intervention Including  a Monthly Food Ration on Food Insecurity Among HIV-Positive Adults During the First Year of Antiretroviral Therapy. | Wrong study design because it is the cohort study. |
| [145] | Sadler et al. Effectiveness of a large-scale food by prescription program in Ethiopia on recovery from malnutrition and HIV progression among HIV+ adults. Ann Nutr Metab. 2013;63:1117. | Wrong design. Program evaluation |
| [146] | Sattler et al. Evaluation of high-protein supplementation in weight-stable HIV-positive subjects with a history of weight loss: a randomized, double-blind, multicenter trial. Am J Clin Nutr. 2008;88(5):1313-21. | Ineligible study populations. Wrong location. |
| [147] | Schaible et al. Malnutrition and infection: complex mechanisms and global impacts. PLoS Med. 2007;4(5):e115. | Wrong study design. Wrong publication type. |
| [148] | Schwenk et al. Oral supplements as adjunctive treatment to nutritional counseling in malnourished HIV-infected patients: randomized controlled trial. Clin Nutr. 1999;18(6):371-4. | Ineligible study populations. Wrong location |
| [149] | Scrimshaw et al. Synergism of nutrition, infection, and immunity: an overview. The American Journal of Clinical Nutrition. 1997;66(2):464S-77S. | Wrong study design. Wrong publication type. |
| [150] | Semba et al. Micronutrient supplements and mortality of HIV-infected adults with pulmonary TB: a controlled clinical trial. The International Journal of Tuberculosis and Lung Disease. 2007;11(8):854-9. | Ineligible study populations. Patients with coinfection (Tuberculosis) |
| [151] | Semba et al. Vitamin A supplementation and human immunodeficiency virus load in injection drug users. The Journal of Infectious Diseases. 1998;177(3):611-6. | Ineligible study populations. Wrong location |
| [152] | Semba et al. Effect of micronutrients and iron supplementation on hemoglobin, iron status, and plasma hepatitis C and HIV RNA levels in female injection drug users: a controlled clinical trial. J Acquir Immune Defic Syndr. 2007;45(3):298-303. | Ineligible study populations. Wrong location |
| [153] | Shabert et al. Glutamine-antioxidant supplementation increases body cell mass in AIDS patients with weight loss: a randomized, double-blind controlled trial. Nutrition. 1999;15(11-12):860-4. | Ineligible study populations. Wrong location |
| [154] | Sidibé et al. The effects of nutritional supplementation on body mass index and CD4 count among adult people living with HIV AIDS on antiretroviral treatment in Conakry, Guinea. Journal of Public Health in Africa. 2018;9(1):36-41. | Wrong study design. Survey study |
| [155] | Song et al. Micronutrient supplementation combined with HAART on nutritional status and immune function of AIDS patients. Acta Microscopica. 2020;29(5):2526-32. | Ineligible study populations. 28 Patients were not on ARV treatment |
| [156] | Stallings et al. High-dose vitamin D3 supplementation in children and young adults with HIV: a randomized, placebo-controlled trial. The Pediatric infectious disease journal. 2015;34(2):e32-40. | Ineligible study populations. Wrong location |
| [157] | Steenhoff et al. Vitamin D₃ Supplementation in Batswana Children and Adults with HIV: A Pilot Double Blind Randomized Controlled Trial. PLoS One. 2015;10(2):e0117123. | Ineligible study populations. Children |
| [158] | Sudfel et al. Efficacy of vitamin D(3) supplementation in reducing incidence of pulmonary tuberculosis and mortality among HIV-infected Tanzanian adults initiating antiretroviral therapy: study protocol for a randomized controlled trial. Trials. 2017;18(1):66. | Ineligible study populations. Patients with coinfection (Tuberculosis) |
| [159] | Süttmann et al. Weight gain and increased concentrations of receptor proteins for tumor necrosis factor after patients with symptomatic HIV infection received fortified nutrition support. J Am Diet Assoc. 1996;96(6):565-9. | Ineligible study populations. Patients were not on ARV treatment |
| [160] | Swaminathan et al. Nutritional Supplementation in HIV-Infected Individuals in South India: A Prospective Interventional Study. Clin Infect Dis. 2010;51(1):51-7. | Ineligible study populations. Patients were not on ARV treatment |
| [161] | Tang et al. Nutrition assessment, counseling, and support interventions to improve health-related outcomes in people living with HIV/AIDS: a systematic review of the literature. J Acquir Immune Defic Syndr. 2015;68 Suppl 3(0 3):S340-9. | Wrong study design. Wrong publication type. |
| [162] | Tirivayi et al. Clinic-Based Food Assistance is Associated with Increased Medication Adherence among HIV-Infected Adults on Long-Term Antiretroviral Therapy in Zambia. J AIDS Clin Res. 2012;3(7):171. | Wrong study design. It was a retrospective cohort study |
| [163] | Urio et al. Improving nutritional status and body composition of people living with HIV/AIDS through regular consumption of locally formulated food supplements in Dar-es-salaam, Tanzania. Ann Nutr Metab. 2009;55:142-. | Wrong comparator. The trial has no control group |
| [164] | Villamor et al. Effect of multivitamin and vitamin A supplements on weight gain during pregnancy among HIV-1-infected women. Am J Clin Nutr. 2002;76(5):1082-90. | Ineligible study populations. Pregnant women. |
| [165] | Wheeler et al. Weight loss as a predictor of survival and disease progression in HIV infection. Terry Beirn Community Programs for Clinical Research on AIDS. J Acquir Immune Defic Syndr Hum Retrovirol. 1998;18(1):80-5. | Wrong study design. Wrong publication type. |
| [166] | Winter et al. Arthrospira platensis as nutrition supplement for adults infected by the human immunodeficiency virus. Ann Nutr Metab. 2013;63:1180. | No full text published. |
| [167] | Winter et al. The Effect of Arthrospira platensis Capsules on CD4 T-Cells and Antioxidative Capacity in a Randomized Pilot Study of Adult Women Infected with Human Immunodeficiency Virus Not under HAART in Yaoundé, Cameroon. Nutrients. 2014;6(7):2973-86. | Ineligible study populations. Patients were not on ARV treatment. |
| [168] | Yamani et al. Use of spirulina a supplement for nutritional management of HIV-infected patients: Study in Bangui, Central African Republic. Medecine tropicale : Revue du Corps de Santé Colonial. 2009;69(1):66-70. | Ineligible study populations. Patients were not on ARV treatment |
| [169] | Young. Effects of micronutrient supplementation on morbidity and mortality among HIV-infected individuals – a summary of the evidence. S Afr Med J. 2006;96(10):1062-4. | Wrong study design. Wrong publication type. |
| [170] | Zhao et al. Effect of micronutrients on the immune status of human immunodeficiency virus-positive individuals. Zhongguo Yi Xue Ke Xue Yuan Xue Bao. 2010;32(3):340-2. | Ineligible study populations. Wrong location |
| [171] | Zulu et al. Assessing the impact of a food supplement on the nutritional status and body composition of HIV-infected Zambian women on ARVs. BMC Public Health. 2011;11(1):714. | Wrong design: Longitudinal |

**References**

1. Adu-Afarwuah S, Lartey A, Brown KH, Zlotkin S, Briend A, Dewey KG. Randomized comparison of 3 types of micronutrient supplements for home fortification of complementary foods in Ghana: effects on growth and motor development. Am J Clin Nutr. 2007;86(2):412-20.

2. Actrn. What is the effect of increasing dietary resistant starch on gut health and immunity in HIV-positive adults in India and is a feeding trial feasible? 2019 [updated 2019]. Available from: <https://www.cochranelibrary.com/central/doi/10.1002/central/CN-01970198/full>.

3. Ahoua L, Umutoni C, Huerga H, Minetti A, Szumilin E, Balkan S, et al. Nutrition outcomes of HIV-infected malnourished adults treated with ready-to-use therapeutic food in sub-Saharan Africa: a longitudinal study. J Int AIDS Soc. 2011;14:2.

4. Almeida LB, Segurado AC, Duran AC, Jaime PC. Impact of a nutritional counseling program on prevention of HAART-related metabolic and morphologic abnormalities. AIDS Care. 2011;23(6):755‐63.

5. Allard JP, Aghdassi E, Chau J, Tam C, Kovacs CM, Salit IE, Walmsley SL. Effects of vitamin E and C supplementation on oxidative stress and viral load in HIV-infected subjects. AIDS. 1998;12(13):1653-9.

6. Alo C, Ogbonnaya LU, Azuogu BN. Effects of nutrition counseling and monitoring on the weight and hemoglobin of patients receiving antiretroviral therapy in Eebonyi State, Southeast Nigeria. HIV/AIDS - Research and Palliative Care. 2014;6:91-7.

7. Amador-Licona N, Diaz-Murillo TA, Gabriel-Ortiz G, Pacheco-Moises FP, Pereyra-Nobara TA, Guizar-Mendoza JM, et al. Omega 3 Fatty Acids Supplementation and Oxidative Stress in HIV-Seropositive Patients. A Clinical Trial. PLoS One. 2016;11(3):e0151637.

8. Ammassari A, Murri R, Pezzotti P, Trotta MP, Ravasio L, De Longis P, et al. Self-reported symptoms and medication side effects influence adherence to highly active antiretroviral therapy in persons with HIV infection. J Acquir Immune Defic Syndr. 2001;28(5):445-9.

9. Anabwani G, Navario P. Nutrition and HIV/AIDS in sub-Saharan Africa: an overview. Nutrition. 2005;21(1):96-9.

10. Andersen AB, Thurnham D, Tomkins A, Mussa K, Masilingi C, Fue E, et al. Effects on mortality of a nutritional intervention for malnourished HIV-infected adults referred for antiretroviral therapy: A randomised controlled trial. BMC Med. 2015;13(1).

11. Anukam KC, Osazuwa EO, Osadolor HB, Bruce AW, Reid G. Yogurt containing probiotic Lactobacillus rhamnosus GR-1 and L. reuteri RC-14 helps resolve moderate diarrhea and increases CD4 count in HIV/AIDS patients. J Clin Gastroenterol. 2008;42(3):239-43.

12. Arsenault JE, Aboud S, Manji KP, Fawzi WW, Villamor E. Vitamin supplementation increases risk of subclinical mastitis in HIV-infected women. J Nutr. 2010;140(10):1788-92.

13. Asdamongkol N, Phanachet P, Sungkanuparph S. Low plasma zinc levels and immunological responses to zinc supplementation in HIV-infected patients with immunological discordance after antiretroviral therapy. Jpn J Infect Dis. 2013;66(6):469-74.

14. Ashenafi S, Amogne W, Kassa E, Gebreselassie N, Bekele A, Aseffa G, et al. Daily nutritional supplementation with vitamin d3 and phenylbutyrate to treatment-naïve hiv patients tested in a randomized placebo-controlled trial. Nutrients. 2019;11(1).

15. Audain KA, Zotor FB, Amuna P, Ellahi B. Food supplementation among HIV-infected adults in Sub-Saharan Africa: Impact on treatment adherence and weight gain. Proc Nutr Soc. 2015;74(4):517-25.

16. Ayuba GI, Jensen GS, Benson KF, Okubena AM, Okubena O. Clinical efficacy of a West African sorghum bicolor-based traditional herbal preparation Jobelyn shows increased hemoglobin and CD4+ T-lymphocyte counts in HIV-positive patients. J Altern Complement Med. 2014;20(1):53‐6.

17. Azabji-Kenfack M, Dikosso SE, Loni EG, Onana EA, Sobngwi E, Gbaguidi E, et al. Potential of Spirulina Platensis as a Nutritional Supplement in Malnourished HIV-Infected Adults in Sub-Saharan Africa: A Randomised, Single-Blind Study. Nutr Metab Insights. 2011;4:29-37.

18. Badiane A, Diouf A, Manga NM, Sow PS, Idohou- Dossou N, Wade S. Increasing energy and zinc intakes to level recommended by who improve fat-free mass but not zinc status in HIV/aids people. Ann Nutr Metab. 2013;63:204.

19. Baeten JM, McClell, RS, Overbaugh J, Richardson BA, Emery S, et al. Vitamin A supplementation and human immunodeficiency virus type 1 shedding in women: Results of a randomized clinical trial. J Infect Dis. 2002;185(8):1187-91.

20. Baingana RK, Juuko JF, Mokori A, Wangwe U, Babirye F, Bak, et al. Effect of food supplementation on body composition of people with HIV/AIDS in Uganda. Ann Nutr Metab. 2009;55:260-.

21. Bakeine J, Mathias PM, Mugyeni PN. The effect of early nutritional supplementation with Nutrifil or Corn Soya Blend on the nutritional and immune status of adults with HIV infection in Uganda. Proceedings of the nutritional society. 1997;56(3):282A.

22. Black MM, Baqui AH, Zaman K, Ake Persson L, El Arifeen S, Le K, et al. Iron and zinc supplementation promote motor development and exploratory behavior among Bangladeshi infants. AJCN. 2004;80(4):903-10.

23. Bang UC, Kolte L, Hitz M, Schierbeck LL, Nielsen SD, Benfield T, Jensen JE. The effect of cholecalciferol and calcitriol on biochemical bone markers in HIV type 1-infected males: results of a clinical trial. AIDS Res Hum Retroviruses. 2013;29(4):658-64.

24. Bang U, Kolte L, Hitz M, Dam Nielsen S, Schierbeck LL, Andersen O, et al. Correlation of increases in 1,25-dihydroxyvitamin D during vitamin D therapy with activation of CD4+ T lymphocytes in HIV-1-infected males. HIV Clin Trials. 2012;13(3):162-70.

25. Bationo F, Ouédraogo G, Kou, a S, Sondo B. Impact of spirulina on the evolution of the anthropometric, biochemical and haematological parameters of HIV-infected adults in Ouagadougou, Burkina Faso. Ann Nutr Metab. 2013;63:444.

26. Batterham MJ, Garsia R. A comparison of megestrol acetate, nandrolone decanoate and dietary counselling for HIV associated weight loss. Int J Androl. 2001;24(4):232-40.

27. Baum MK, Lai S, Sales S, Page JB, Campa A. Randomized, controlled clinical trial of zinc supplementation to prevent immunological failure in HIV-infected adults. Clin Infect Dis. 2010;50(12):1653-60.

28. Baum MK, Campa A, Lai S, Sales Martinez S, Tsalaile L, Burns P, et al. Effect of micronutrient supplementation on disease progression in asymptomatic, antiretroviral-naive, HIV-infected adults in Botswana: A randomized clinical trial. JAMA. 2013;310(20):2154-63.

29. Beckett AG, Humphries D, Jerome JG, Teng JE, Ulysse P, Ivers LC. Acceptability and use of ready-to-use supplementary food compared to corn-soy blend as a targeted ration in an HIV program in rural Haiti: a qualitative study. AIDS Res Ther. 2016;13:11.

30. Berneis K, Battegay M, Bassetti S, Nuesch R, Leisibach A, Bilz S, Keller U. Nutritional supplements combined with dietary counselling diminish whole body protein catabolism in HIV-infected patients. Eur J Clin Invest. 2000;30(1):87-94.

31. Bhima K, Mtimuni B, Matumba L. Tackling protein-energy under-nutrition among resource-limited people living with HIV/AIDS in Malawi using soybean-enriched maize-based stiff porridge (nsima): A pilot study. Nutr Diet. 2019;76(3):257-62.

32. Boontanondha P, Nimitphong H, Musikarat S, Ragkho A, Kiertiburanakul S. Vitamin D and Calcium Supplement Attenuate Bone Loss among HIVInfected Patients Receiving Tenofovir Disoproxil Fumarate/Emtricitabine/ Efavirenz: An Open-Label, Randomized Controlled Trial. Current HIV research. 2020;18(1):52-62.

33. Burbano X, Miguez-Burbano MJ, McCollister K, Zhang G, Rodriguez A, Ruiz P, et al. Impact of a selenium chemoprevention clinical trial on hospital admissions of HIV-infected participants. HIV Clin Trials. 2002;3(6):483-91.

34. Bushen OY, Davenport JA, Lima AB, Piscitelli SC, Uzgiris AJ, Silva TM, et al. Diarrhea and reduced levels of antiretroviral drugs: improvement with glutamine or alanyl-glutamine in a randomized controlled trial in northeast Brazil. Clin Infect Dis. 2004;38(12):1764-70.

35. Cárcamo C, Hooton T, Weiss NS, Gilman R, Wener MH, Chavez V, et al. Randomized controlled trial of zinc supplementation for persistent diarrhea in adults with HIV-1 infection. J Acquir Immune Defic Syndr. 2006;43(2):197‐201.

36. Chotivichien S, Arab L, Prasithsirikul W, Manosuthi W, Sinawat S, Detels R. Effect of nutritional counseling on low-density lipoprotein cholesterol among Thai HIV-infected adults receiving antiretroviral therapy. AIDS Care. 2016;28(2):257-65.

37. Clark RH, Feleke G, Din M, Yasmin T, Singh G, Khan FA, Rathmacher JA. Nutritional treatment for acquired immunodeficiency virus-associated wasting using beta-hydroxy beta-methylbutyrate, glutamine, and arginine: a randomized, double-blind, placebo-controlled study. JPEN. 2000;24(3):133-9.

38. Coates J, Sadler K, Bontrager E, Ghosh S, Suri D, Kidane Y. Persistence of recovery from malnutrition and HIV progression among HIV+ adults graduating from the ethiopian food by prescription program. Ann Nutr Metab. 2013;63:527.

39. Coelho L, Cardoso SW, Luz PM, Hoffman RM, Mendonça L, Veloso VG, et al. Vitamin D3 supplementation in HIV infection: effectiveness and associations with antiretroviral therapy. Nutr J. 2015;14:81.

40. Coghill AE, Schenk JM, Mahkoul Z, Orem J, Phipps W, Casper C. Omega-3 decreases IL-6 levels in HIV and human herpesvirus-8 coinfected patients in Uganda. AIDS. 2018;32(4):505-12.

41. Coodley GO, Coodley MK, Lusk R, Green TR, Bakke AC, Wilson D, et al. Beta-carotene in HIV infection: an extended evaluation. AIDS. 1996;10(9):967-73.

42. Coodley GO, Nelson HD, Loveless MO, Folk C. Beta-carotene in HIV infection. J Acquir Immune Defic Syndr. 1993;6(3):272-6.

43. de Luis D, Aller R, Bachiller P, González-Sagrado M, de Luis J, Izaola O, et al. [Isolated dietary counselling program versus supplement and dietary counselling in patients with human immunodeficiency virus infection]. Med Clin (Barc). 2003;120(15):565-7.

44. de Pee S, Semba RD. Role of nutrition in HIV infection: review of evidence for more effective programming in resource-limited settings. Food Nutr Bull. 2010;31(4):S313-44.

45. Derose KP, Felician M, Han B, Palar K, Ramírez B, Farías H, Martínez H. A pre-post pilot study of peer nutritional counseling and food insecurity and nutritional outcomes among antiretroviral therapy patients in Honduras. BMC nutrition. 2015;1.

46. Diouf A, Badiane A, Manga NM, Idohou-Dossou N, Sow PS, Wade S. Daily consumption of ready-to-use peanut-based therapeutic food increased fat free mass, improved anemic status but has no impact on the zinc status of people living with HIV/AIDS: a randomized controlled trial. BMC Public Health. 2016;16:1.

47. Dougherty KA, Schall JI, Zemel BS, Tuluc F, Hou X, Rutstein RM, Stallings VA. Safety and Efficacy of High-Dose Daily Vitamin D3 Supplementation in Children and Young Adults Infected With Human Immunodeficiency Virus. Journal of the Pediatric Infectious Diseases Society. 2014;3(4):294-303.

48. Ekstr ML, Heylen E, Pereira M, D'Souza J, Nair S, Mazur A, et al. A Behavioral Adherence Intervention Improves Rates of Viral Suppression Among Adherence-Challenged People Living with HIV in South India. AIDS Behav. 2020;24(7):2195-205.

49. Ernst J, Ettyang G, Neumann C. High nutrition biscuits as a supplement to increase animal protein in diets of HIV-infected kenyan women and their children. Ann Nutr Metab. 2013;63:144.

50. Etminani-Esfahani M, Khalili H, Jafari S, Abdollahi A, Dashti-Khavidaki S. Effects of vitamin D supplementation on the bone specific biomarkers in HIV infected individuals under treatment with efavirenz. BMC Res Notes. 2012;5:204.

51. Faber M, Kvalsvig JD, Lombard CJ, Benade AJ. Effect of a fortified maize-meal porridge on anemia, micronutrient status, and motor development of infants. Am J Clin Nutr. 2005;82(5):1032-9.

52. Fawzi WW, Msamanga GI, Kupka R, Spiegelman D, Villamor E, Mugusi F, et al. Multivitamin supplementation improves hematologic status in HIV-infected women and their children in Tanzania. Am J Clin Nutr. 2007;85(5):1335-43.

53. Fawzi WW, Msamanga GI, Spiegelman D, Wei R, Kapiga S, Villamor E, et al. A randomized trial of multivitamin supplements and HIV disease progression and mortality. N Engl J Med. 2004;351(1):23-32.

54. Ferreira RDS, Guimarães RDCA, Pontes ERJC, Mendonça LABM, Freitas KDC, Hiane PA. Effectiveness of a bioactive food compound in anthropometric measures of individuals with HIV/AIDS: A nonrandomized trial. PLoS One. 2018;13(2).

55. Flax VL, Adair LS, Allen LH, Shahab-Ferdows S, Hampel D, Chasela CS, et al. Plasma micronutrient concentrations are altered by antiretroviral therapy and lipid-based nutrient supplements in lactating HIV-infected Malawian women. J Nutr. 2015;145(8):1950-7.

56. Freiberg MS, Cheng DM, Gnatienko N, Blokhina E, Coleman SM, Doyle MF, et al. Effect of Zinc Supplementation vs Placebo on Mortality Risk and HIV Disease Progression among HIV-Positive Adults with Heavy Alcohol Use: A Randomized Clinical Trial. JAMA Network Open. 2020.

57. Ghosh S, Suri D, Bontrager E, Coates J, Rogers B, Kidane Y, Sadler K. Factors associated with recovery among ethiopian malnourished HIV patients (pre art and art) that received food by prescription. Ann Nutr Metab. 2013;63:816.

58. Giacomet V, Vigano A, Manfredini V, Cerini C, Bedogni G, Mora S, et al. Cholecalciferol supplementation in HIV-infected youth with vitamin D insufficiency: effects on vitamin D status and T-cell phenotype: a randomized controlled trial. HIV Clin Trials. 2013;14(2):51-60.

59. Gnatienko N, Freiberg MS, Blokhina E, Yaroslavtseva T, Bridden C, Cheng DM, et al. Design of a randomized controlled trial of zinc supplementation to improve markers of mortality and HIV disease progression in HIV-positive drinkers in St. Petersburg, Russia. HIV Clin Trials. 2018;19(3):101-11.

60. Goncalves J, Silva MCA, Roma EH, Grinsztejn B, de Lemos AD, Moreira NG, et al. Iron intake is positively associated with viral load in antiretroviral naive Brazilian men living with HIV. Mem Inst Oswaldo Cruz. 2019;114.

61. González-Hernández LA, Jave-Suarez LF, Fafutis-Morris M, Montes-Salcedo KE, Valle-Gutierrez LG, Campos-Loza AE, et al. Synbiotic therapy decreases microbial translocation and inflammation and improves immunological status in HIV-infected patients: a double-blind randomized controlled pilot trial. Nutr J. 2012;11:90.

62. Green JA, Lewin SR, Wightman F, Lee M, Ravindran TS, Paton NI. A randomised controlled trial of oral zinc on the immune response to tuberculosis in HIV-infected patients. The international journal of tuberculosis and lung disease. 2005;9(12):1378-84.

63. Grigoletti SS, Guindani G, Moraes RS, Ribeiro JP, Sprinz E. Short-term folinic acid supplementation improves vascular reactivity in HIV-infected individuals: a randomized trial. Nutrition. 2013;29(6):886‐91.

64. Grobler L, Nagpal S, Sudarsanam TD, Sinclair D. Nutritional supplements for people being treated for active tuberculosis. The Cochrane database of systematic reviews. 2016;2016(6):Cd006086.

65. Haider BA, Spiegelman D, Hertzmark E, o D, Duggan C, Makubi A, et al. Anemia, iron deficiency, and iron supplementation in relation to mortality among HIV-infected patients receiving highly active antiretroviral therapy in Tanzania. Am J Trop Med Hyg. 2019;100(6):1512-20.

66. Hardon AP, Akurut D, Comoro C, Ekezie C, Irunde HF, Gerrits T, et al. Hunger, waiting time and transport costs: time to confront challenges to ART adherence in Africa. AIDS Care. 2007;19(5):658-65.

67. Higgins JP, Thompson SG. Quantifying heterogeneity in a meta-analysis. Stat Med. 2002;21(11):1539-58.

68. Humphrey JH, Quinn T, Fine D, Lederman H, Yamini-Roodsari S, Wu LS, et al. Short-term effects of large-dose vitamin A supplementation on viral load and immune response in HIV-infected women. J Acquir Immune Defic Syndr Hum Retrovirol. 1999;20(1):44-51.

69. Hurwitz BE, Klaus JR, Llabre MM, Gonzalez A, Lawrence PJ, Maher KJ, et al. Suppression of human immunodeficiency virus type 1 viral load with selenium supplementation: a randomized controlled trial. Arch Intern Med. 2007;167(2):148-54.

70. Irvine SL, Hummelen R, Hekmat S. Probiotic yogurt consumption may improve gastrointestinal symptoms, productivity, and nutritional intake of people living with human immunodeficiency virus in Mwanza, Tanzania. Nutr Res. 2011;31(12):875-81.

71. Irvine SL, Hummelen R, Hekmat S, W. N. Looman C, Habbema JDF, Reid G. Probiotic yogurt consumption is associated with an increase of CD4 count among people living with HIV/AIDS. J Clin Gastroenterol. 2010;44(9):e201-e5.

72. Isabirye N, Ezeamama AE, Kyeyune-Bakyayita R, Bagenda D, Fawzi WW, Guwatudde D. Dietary Micronutrients and Gender, Body Mass Index and Viral Suppression Among HIV-Infected Patients in Kampala, Uganda. IJMA. 2020;9(3):337-49.

73. Isanaka S, Mugusi F, Fawzi WW. Standard-dose vs high-dose multivitamin supplements for HIV--reply. JAMA. 2013;309(6):546.

74. Isanaka S, Mugusi F, Hawkins C, Spiegelman D, Okuma J, Aboud S, et al. Effect of high-dose vs standard-dose multivitamin supplementation at the initiation of HAART on HIV disease progression and mortality in Tanzania: A randomized controlled trial. JAMA. 2012;308(15):1535-44.

75. Isrctn. A nutritional supplement for human immunodeficiency virus (HIV) antibody positive patients at Mengo Hospital, Kampala, Uganda. <http://wwwwhoint/trialsearch/Trial2aspx?TrialID=ISRCTN42274642>. 2005.

76. Isrctn. Impact of Spirulina platensis supplementation on general health status of HIV infected patients in Burkina Faso. <http://wwwwhoint/trialsearch/Trial2aspx?TrialID=ISRCTN83770226>. 2007.

77. Ivers LC, Teng JE, Gregory Jerome J, Bonds M, Freedberg KA, Franke MF. A randomized trial of ready-to-use supplementary food versus corn-soy blend plus as food rations for HIV-infected adults on antiretroviral therapy in rural haiti. Clin Infect Dis. 2014;58(8):1176-84.

78. James P, Friis H, Woodd S, Rehman AM, PrayGod G, Kelly P, et al. Minimal impact of an iron-fortified lipid-based nutrient supplement on Hb and iron status: A randomised controlled trial in malnourished HIV-positive African adults starting antiretroviral therapy. Br J Nutr. 2015;114(3):387-97.

79. Ji G, Qi R, Wang H, Feng C, Leng J. A planting and eating soybean project for people living with HIV/AIDS in rural Anhui - A pilot study in China. AIDS Care. 2010;22(1):126-32.

80. Jiamto S, Chaisilwattana P, Pepin J, Suttent R, Mahakkanukrauh B, Filteau S, et al. A randomized placebo-controlled trial of the impact of multiple micronutrient supplementation on HIV-1 genital shedding among Thai subjects [1]. J Acquir Immune Defic Syndr. 2004;37:1216-8.

81. Jiamton S, Pepin J, Suttent R, Filteau S, Mahakkanukrauh B, Hanshaoworakul W, et al. A randomized trial of the impact of multiple micronutrient supplementation on mortality among HIV-infected individuals living in Bangkok. AIDS. 2003;17(17):2461-9.

82. Kabagambe EK, Ezeamama AE, Guwatudde D, Campos H, Fawzi WW. Plasma n-6 fatty acid levels are associated with CD4 cell counts, hospitalization, and mortality in HIV-infected patients. JAIDS. 2016;73(5):598-605.

83. Kaducu FO, Okia SA, Upenytho G, Elfstr, L, Florén CH. Effect of bovine colostrum-based food supplement in the treatment of HIV-associated diarrhea in Northern Uganda: A randomized controlled trial. Indian J Gastroenterol. 2011;30(6):270-6.

84. Kaiser JD, Campa AM, Ondercin JP, Leoung GS, Pless RF, Baum MK. Micronutrient supplementation increases CD4 count in HIV-infected individuals on highly active antiretroviral therapy: a prospective, double-blinded, placebo-controlled trial. J Acquir Immune Defic Syndr. 2006;42(5):523-8.

85. Kamwesiga J, Mutabazi V, Kayumba J, Tayari JCK, Uwimbabazi JC, Batanage G, et al. Effect of selenium supplementation on CD4R T-cell recovery, viral suppression and morbidity of HIV-infected patients in Rwanda: A randomized controlled trial. AIDS. 2015;29(9):1045-52.

86. Kamwesiga J, Mutabazi V, Tayari JC, Fay H, Seely D, Silva C, et al. Effect of selenium supplementation on CD4 depletion in rwandan HIV patients: A randomized trial. Top Antivir Med. 2014;22:267-8.

87. Karsegard VL, Raguso CA, Genton L, Hirschel B, Pichard C. L-ornithine alpha-ketoglutarate in HIV infection: effects on muscle, gastrointestinal, and immune functions. Nutrition. 2004;20(6):515-20.

88. Keithley JK, Swanson B, Zeller JM, Sha BE, Cohen M, Hershow R, Novak R. Comparison of standard and immune-enhancing oral formulas in asymptomatic HIV-infected persons: a multicenter randomized controlled clinical trial. JPEN. 2002;26(1):6-14.

89. Kelly P, Katubulushi M, Todd J, a R, Yambayamba V, Fwoloshi M, et al. Micronutrient supplementation has limited effects on intestinal infectious disease and mortality in a Zambian population of mixed HIV status: a cluster randomized trial. Am J Clin Nutr. 2008;88(4):1010-7.

90. Kelly P, Musonda R, Kafwembe E, Kaetano L, Keane E, Farthing M. Micronutrient supplementation in the AIDS diarrhoea-wasting syndrome in Zambia: a randomized controlled trial. AIDS. 1999;13(4):495-500.

91. Kelly P, Shawa T, erson I. Gastric hypochlorhydria and intestinal barrier dysfunction in HIV infection is not dependent on nutrition: A randomised controlled trial of supplementation. Gut. 2009;58:A82-A3.

92. Kelly P, Shawa T, Mwanamakondo S, Soko R, Smith G, Barclay GR, erson IR. Gastric and intestinal barrier impairment in tropical enteropathy and HIV: Limited impact of micronutrient supplementation during a randomised controlled trial. BMC Gastroenterol. 2010;10.

93. Kotler DP. Malnutrition in HIV infection and AIDS. AIDS. 1989;3 Suppl 1:S175-80.

94. Kotler DP, Rosenbaum K, Wang J, Pierson RN. Studies of body composition and fat distribution in HIV-infected and control subjects. J Acquir Immune Defic Syndr Hum Retrovirol. 1999;20(3):228-37.

95. Lebouché B, Yero A, Shi T, Farnos O, Singer J, Kema I, et al. Impact of extended-release niacin on immune activation in HIV-infected immunological non-responders on effective antiretroviral therapy. HIV research & clinical practice. 2020;21(6):182-90.

96. Macallan DC. Sir David Cuthbertson Prize Medal Lecture. Metabolic abnormalities and wasting in human immunodeficiency virus infection. The Proceedings of the Nutrition Society. 1998;57(3):373-80.

97. Macallan DC, McNurlan MA, Milne E, Calder AG, Garlick PJ, Griffin GE. Whole-body protein turnover from leucine kinetics and the response to nutrition in human immunodeficiency virus infection. The American journal of clinical nutrition. 1995;61(4):818-26.

98. Macallan DC, Noble C, Baldwin C, Foskett M, McManus T, Griffin GE. Prospective analysis of patterns of weight change in stage IV human immunodeficiency virus infection. The American journal of clinical nutrition. 1993;58(3):417-24.

99. Macallan DC, Noble C, Baldwin C, Jebb SA, Prentice AM, Coward WA, et al. Energy expenditure and wasting in human immunodeficiency virus infection. The New England journal of medicine. 1995;333(2):83-8.

100. Mallewa JE, Szubert AJ, Berkley J, Nkomani S, Siika A, Mugyenyi P, et al. A randomized trial of ready-to-use supplementary food at art initiation in Africa. Top Antivir Med. 2017;25(1):45s-6s.

101. Mallewa J, Szubert AJ, Mugyenyi P, Chidziva E, Thomason MJ, Chepkorir P, et al. Effect of ready-to-use supplementary food on mortality in severely immunocompromised HIV-infected individuals in Africa initiating antiretroviral therapy (REALITY): an open-label, parallel-group, randomised controlled trial. The Lancet HIV. 2018;5(5):e231-e40.

102. Maluccio JA, Palermo T, Kadiyala S, Rawat R. Improving Health-Related Quality of Life among People Living with HIV: Results from an Impact Evaluation of a Food Assistance Program in Uganda. PLoS One. 2015;10(8):e0135879.

103. Manary M, Ndekha M, van Oosterhout JJ. Supplementary feeding in the care of the wasted HIV infected patient. Malawi Med J. 2010;22(2):46-9.

104. Mansouri F, Janbakhsh A, Vaziri S, Sayad B, Afsharian M, Hosseinpor F, Mahdavian B. Comparative study of levamisole-selenium supplementation effect on CD4 increase in HIV/AIDS patients. Caspian Journal of Internal Medicine. 2011;2(2):218-21.

105. Marazzi MC, Liotta G, Germano P, Guidotti G, Altan AD, Ceffa S, et al. Excessive early mortality in the first year of treatment in HIV type 1-infected patients initiating antiretroviral therapy in resource-limited settings. AIDS Res Hum Retroviruses. 2008;24(4):555-60.

106. Marcel AK, Ekali LG, Eugene S, Arnold OE, Sandrine ED, von der Weid D, et al. The effect of Spirulina platensis versus soybean on insulin resistance in HIV-infected patients: a randomized pilot study. Nutrients. 2011;3(7):712-24.

107. Martí‐Carvajal AJ, Cruciani M. Pharmacological interventions for treating dyslipidemia in patients with HIV infection. Cochrane Database Syst Rev. 2018;2018(2).

108. Mburu AS, Thurnham DI, Mwaniki DL, Muniu EM, Alumasa FM. The influence of inflammation on plasma zinc concentration in apparently healthy, HIV+ Kenyan adults and zinc responses after a multi-micronutrient supplement. Eur J Clin Nutr. 2010;64(5):510‐7.

109. Mburu ASW, Thurnham DI, Mwaniki DL, Muniu EM, Alumasa F, De Wagt A. The influence and benefits of controlling for inflammation on plasma ferritin and hemoglobin responses following a multi-micronutrient supplement in apparently healthy, HIV+ Kenyan adults. J Nutr. 2008;138(3):613-9.

110. McClell, RS, Baeten JM, Overbaugh J, Richardson BA, aliya K, et al. Micronutrient supplementation increases genital tract shedding of HIV-1 in women: Results of a randomized trial. JAIDS. 2004;37(5):1657-63.

111. McCoy SI, Njau P, Fahey C, Czaicki N, Kapologwe N, Kadiyala S, et al. A randomized study of short-term conditional cash and food assistance to improve adherence to antiretroviral therapy among food insecure adults with HIV infection in Tanzania. J Int AIDS Soc. 2016;19:88.

112. McGrath N, Bellinger D, Robins J, Msamanga GI, Tronick E, Fawzi WW. Effect of maternal multivitamin supplementation on the mental and psychomotor development of children who are born to HIV-1-infected mothers in Tanzania. Pediatrics. 2006;117(2):e216-25.

113. Mensah KA, Okyere P, Doku PN. An evaluation of a community-based food supplementation for people living with HIV in Ghana: implications for community-based interventions in Ghana. BMC Res Notes. 2015;8:519.

114. Moore MA, Wandera RC, Xia YM, Du SH, Butler JA, Whanger PD. Selenium supplementation of Chinese women with habitually low selenium intake increases plasma selenium, plasma glutathione peroxidase activity, and milk selenium, but not milk glutathione peroxidase activity. J Nutr Biochem. 2000;11(6):341-7.

115. Motswagole BS, Mongwaketse TC, Mokotedi M, Kobue-Lekalake RI, Bulawayo BT, Thomas TS, et al. The efficacy of micronutrient-fortified sorghum meal in improving the immune status of HIV-positive adults. Ann Nutr Metab. 2013;62(4):323-30.

116. Munkombwe D, Muungo TL, Michelo C, Kelly P, Chirwa S, Filteau S. Lipid-based nutrient supplements containing vitamins and minerals attenuate renal electrolyte loss in HIV/AIDS patients starting antiretroviral therapy: A randomized controlled trial in Zambia. Clinical Nutrition ESPEN. 2016;13:e8-e14.

117. Namulema E, Sparling J, Foster HD. When the nutritional supplements stop: evidence from a double-blinded, HIV clinical trial at Mengo Hospital, Kampala, Uganda. Journal of orthomolecular medicine. 2008;23(3):130‐2.

118. Namulemia E, Sparling J, Foster HD. Nutritional supplements can delay the progression of AIDS in HIV-infected patients: Results from a double-blinded, clinical trial at Mengo Hospital, Kampala, Uganda. Journal of Orthomolecular Medicine. 2007;22(3):129-36.

119. Nct. Study of Impacts of Food Supplementation on Malnourished HIV-Infected Adults in Kenya. <https://clinicaltrialsgov/show/NCT00838292>. 2009.

120. Nct. The Role of Probiotics in HIV Patients With Immunological Non-Responder. <https://clinicaltrialsgov/show/NCT03568812>. 2018.

121. Nct. " Arthrospira Platensis" as Nutrition Supplementation for Female Adult Patients Infected by HIV in Yaoundé Cameroon. <https://clinicaltrialsgov/show/NCT01084382>. 2010.

122. Ndekha MJ, Van Oosterhout JJG, Zijlstra EE, Manary M, Saloojee H, Manary MJ. Supplementary feeding with either ready-to-use fortified spread or corn-soy blend in wasted adults starting antiretroviral therapy in Malawi: Randomised, investigator blinded, controlled trial. BMJ (Online). 2009;338(7706):1309-11.

123. Ndekha M, Van Oosterhout JJG, Saloojee H, Pettifor J, Manary M. Nutritional status of Malawian adults on antiretroviral therapy 1 year after supplementary feeding in the first 3 months of therapy. Trop Med Int Health. 2009;14(9):1059-63.

124. Ngo-Matip ME, Pieme CA, Azabji-Kenfack M, Biapa PC, Germaine N, Heike E, et al. Effects of Spirulina platensis supplementation on lipid profile in HIV-infected antiretroviral naive patients in Yaounde-Cameroon: a randomized trial study. Lipids Health Dis. 2014;13:191.

125. Ngo-Matip ME, Pieme CA, Azabji-Kenfack M, Moukette BM, Korosky E, Stefanini P, et al. Impact of daily supplementation of Spirulina platensis on the immune system of naïve HIV-1 patients in Cameroon: A 12-months single blind, randomized, multicenter trial. Nutr J. 2015;14(1).

126. Odunukwe N, Okwuzu J, Okwuraiwe A, Gbajabiamila T, Musa Z, Ezeobi P, et al. Selenium as adjunct to HAART in the management of HIV/Hepatitis B virus coinfection: a randomized open label study. African Journal of Clinical and Experimental Microbiology. 2016;17(3):197-204.

127. Ogbuagu EN, Ufearo S, Ogbuagu CN, Okonkwo R. CD4 pattern in HIV positive patients on HAART exposed to moringa oleifera leaf powder in south east Nigeria. Int J Infect Dis. 2016;45:267.

128. Oguntibeju OO, Van den Heever WMJ, Van Schalkwyk FE. Supplementation effect on Body Weight and BMI of HIV-positive/AIDS patients. International Journal of Pharmacology. 2007;3(1):120-2.

129. Oguntibeju O, van den Heever WM, Van Schalkwyk FE. A locally produced nutritional supplement in community-based HIV and AIDS patients. Int J Palliat Nurs. 2007;13(4):154-62.

130. Oketch JA, Paterson M, Maunder EW, Rollins NC. Too little, too late: Comparison of nutritional status and quality of life of nutrition care and support recipient and non-recipients among HIV-positive adults in KwaZulu-Natal, South Africa. Health Policy. 2011;99(3):267-76.

131. Oliveira JM, Rondo PH, Lima LR, Fortuna ES, Yudkin JS. Effects of a Low Dose of Fish Oil on Inflammatory Markers of Brazilian HIV-Infected Adults on Antiretroviral Therapy: A Randomized, Parallel, Placebo-Controlled Trial. Nutrients. 2015;7(8):6520-8.

132. Oliveira JM, Rondo PH, Yudkin JS, Souza JM, Pereira TN, Catalani AW, et al. Effects of fish oil on lipid profile and other metabolic outcomes in HIV-infected patients on antiretroviral therapy: a randomized placebo-controlled trial. Int J STD AIDS. 2014;25(2):96-104.

133. Olofin IO, Spiegelman D, Aboud S, Duggan C, Danaei G, Fawzi WW. Supplementation with multivitamins and vitamin A and incidence of malaria among HIV-infected tanzanian women. J Acquir Immune Defic Syndr. 2014;67:S173-S8.

134. Overton ET, Chan ES, Brown TT, Tebas P, McComsey GA, Melbourne KM, et al. Vitamin D and Calcium Attenuate Bone Loss With Antiretroviral Therapy Initiation: A Randomized Trial. Ann Intern Med. 2015;162(12):815-24.

135. Palar K, Derose KP, Linnemayr S, Smith A, Farías H, Wagner G, Martinez H. Impact of food support on food security and body weight among HIV antiretroviral therapy recipients in Honduras: A pilot intervention trial. AIDS Care. 2015;27(4):409-15.

136. Peters BS, Wierzbicki AS, Moyle G, Nair D, Brockmeyer N. The effect of a 12-week course of omega-3 polyunsaturated fatty acids on lipid parameters in hypertriglyceridemic adult HIV-infected patients undergoing HAART: a randomized, placebo-controlled pilot trial. Clin Ther. 2012;34(1):67‐76.

137. Petrilli AA, Souza SJ, Teixeira AM, Pontilho PM, Souza JM, Luzia LA, Rondo PH. Effect of Chocolate and Yerba Mate Phenolic Compounds on Inflammatory and Oxidative Biomarkers in HIV/AIDS Individuals. Nutrients. 2016;8(5).

138. Praygod G, Blevins M, Woodd S, Rehman A, Kidola J, Friis H, et al. Persistent inflammation on ART is associated with poor nutritional recovery in Zambia. Top Antivir Med. 2015;23:342.

139. PrayGod G, Rehman AM, Wells JCK, Chisenga M, Siame J, Jeremiah K, et al. Effects on body composition and handgrip strength of a nutritional intervention for malnourished HIV-infected adults referred for antiretroviral therapy: a randomised controlled trial. Journal of nutritional science. 2019;8:e19.

140. Rabeneck L, Palmer A, Knowles JB, Seidehamel RJ, Harris CL, Merkel KL, et al. A randomized controlled trial evaluating nutrition counseling with or without oral supplementation in malnourished HIV-infected patients. J Am Diet Assoc. 1998;98(4):434-8.

141. Rawat R, Faust E, Maluccio JA, Kadiyala S. The impact of a food assistance program on nutritional status, disease progression, and food security among people living with HIV in Uganda. JAIDS. 2014;66(1):e15-22.

142. Rawat R, Kadiyala S, McNamara PE. The impact of food assistance on weight gain and disease progression among HIV-infected individuals accessing AIDS care and treatment services in Uganda. BMC Public Health. 2010;10:316.

143. Rehman AM, Woodd S, PrayGod G, Chisenga M, Siame J, Koethe JR, et al. Effects on anthropometry and appetite of vitamins and minerals given in lipid nutritional supplements for malnourished HIV-infected adults referred for antiretroviral therapy: Results from the NUSTART randomized controlled trial. J Acquir Immune Defic Syndr. 2015;68(4):405-12.

144. Rothman J, Kayigamba F, Hills V, Gupta N, Machara F, Niyigena P, Franke MF. The Impact of a Community-Based Intervention Including a Monthly Food Ration on Food Insecurity Among HIV-Positive Adults During the First Year of Antiretroviral Therapy. AIDS Behav. 2018;22(1):154-63.

145. Sadler K, Bontrager E, Coates J, Ghosh S, Suri D, Lorge Rogers B, Kidane Y. Effectiveness of a large-scale food by prescription program in Ethiopia on recovery from malnutrition and HIV progression among HIV+ adults. Ann Nutr Metab. 2013;63:1117.

146. Sattler FR, Rajicic N, Mulligan K, Yarasheski KE, Koletar SL, Zolopa A, et al. Evaluation of high-protein supplementation in weight-stable HIV-positive subjects with a history of weight loss: a randomized, double-blind, multicenter trial. Am J Clin Nutr. 2008;88(5):1313-21.

147. Schaible UE, Kaufmann SH. Malnutrition and infection: complex mechanisms and global impacts. PLoS Med. 2007;4(5):e115.

148. Schwenk A, Steuck H, Kremer G. Oral supplements as adjunctive treatment to nutritional counseling in malnourished HIV-infected patients: randomized controlled trial. Clin Nutr. 1999;18(6):371-4.

149. Scrimshaw NS, SanGiovanni JP. Synergism of nutrition, infection, and immunity: an overview. The American Journal of Clinical Nutrition. 1997;66(2):464S-77S.

150. Semba RD, Kumwenda J, Zijlstra E, Ricks MO, van Lettow M, Whalen C, et al. Micronutrient supplements and mortality of HIV-infected adults with pulmonary TB: a controlled clinical trial. The International Journal of Tuberculosis and Lung Disease. 2007;11(8):854-9.

151. Semba RD, Lyles CM, Margolick JB, Caiaffa WT, Farzadegan H, Cohn S, Vlahov D. Vitamin A supplementation and human immunodeficiency virus load in injection drug users. The Journal of Infectious Diseases. 1998;177(3):611-6.

152. Semba RD, Ricketts EP, Mehta S, Netski D, Thomas D, Kirk G, et al. Effect of micronutrients and iron supplementation on hemoglobin, iron status, and plasma hepatitis C and HIV RNA levels in female injection drug users: a controlled clinical trial. J Acquir Immune Defic Syndr. 2007;45(3):298-303.

153. Shabert JK, Winslow C, Lacey JM, Wilmore DW. Glutamine-antioxidant supplementation increases body cell mass in AIDS patients with weight loss: a randomized, double-blind controlled trial. Nutrition. 1999;15(11-12):860-4.

154. Sidibé S, Delamou A, Kaba ML, Magassouba AS, Samake AT, Dongo YSA, et al. The effects of nutritional supplementation on body mass index and CD4 count among adult people living with HIV AIDS on antiretroviral treatment in Conakry, Guinea. Journal of Public Health in Africa. 2018;9(1):36-41.

155. Song Y, Qiu J, Hou W, Wang D, Yang Y. Micronutrient supplementation combined with HAART on nutritional status and immune function of AIDS patients. Acta Microscopica. 2020;29(5):2526-32.

156. Stallings VA, Schall JI, Hediger ML, Zemel BS, Tuluc F, Dougherty KA, et al. High-dose vitamin D3 supplementation in children and young adults with HIV: a randomized, placebo-controlled trial. The Pediatric infectious disease journal. 2015;34(2):e32-40.

157. Steenhoff AP, Schall JI, Samuel J, Seme B, Marape M, Ratshaa B, et al. Vitamin D₃supplementation in Batswana children and adults with HIV: a pilot double blind randomized controlled trial. PLoS One. 2015;10(2):e0117123.

158. Sudfeld CR, Mugusi F, Aboud S, Nagu TJ, Wang M, Fawzi WW. Efficacy of vitamin D(3) supplementation in reducing incidence of pulmonary tuberculosis and mortality among HIV-infected Tanzanian adults initiating antiretroviral therapy: study protocol for a randomized controlled trial. Trials. 2017;18(1):66.

159. Süttmann U, Ockenga J, Schneider H, Selberg O, Schlesinger A, Gallati H, et al. Weight gain and increased concentrations of receptor proteins for tumor necrosis factor after patients with symptomatic HIV infection received fortified nutrition support. J Am Diet Assoc. 1996;96(6):565-9.

160. Swaminathan S, Padmapriyadarsini C, Yoojin L, Sukumar B, Iliayas S, Karthipriya J, et al. Nutritional supplementation in HIV-infected individuals in south India: A prospective interventional study. Clin Infect Dis. 2010;51(1):51-7.

161. Tang AM, Quick T, Chung M, Wanke CA. Nutrition assessment, counseling, and support interventions to improve health-related outcomes in people living with HIV/AIDS: a systematic review of the literature. J Acquir Immune Defic Syndr. 2015;68 Suppl 3(0 3):S340-9.

162. Tirivayi N, Koethe JR, Groot W. Clinic-Based Food Assistance is Associated with Increased Medication Adherence among HIV-Infected Adults on Long-Term Antiretroviral Therapy in Zambia. J AIDS Clin Res. 2012;3(7):171.

163. Urio EM, Tatala SR, Mgoba CM, Mlingi N, Ndossi GD. Improving nutritional status and body composition of people living with HIV/AIDS through regular consumption of locally formulated food supplements in Dar-Es-Salaam, Tanzania. Ann Nutr Metab. 2009;55:142-.

164. Villamor E, Msamanga G, Spiegelman D, Antelman G, Peterson KE, Hunter DJ, Fawzi WW. Effect of multivitamin and vitamin A supplements on weight gain during pregnancy among HIV-1-infected women. Am J Clin Nutr. 2002;76(5):1082-90.

165. Wheeler DA, Gibert CL, Launer CA, Muurahainen N, Elion RA, Abrams DI, Bartsch GE. Weight loss as a predictor of survival and disease progression in HIV infection. Terry Beirn Community Programs for Clinical Research on AIDS. J Acquir Immune Defic Syndr Hum Retrovirol. 1998;18(1):80-5.

166. Winter F, Emakam F, Kfutwah J, Azabji M, Krawinkel M. Arthrospira platensis as nutrition supplement for adults infected by the human immunodeficiency virus. Ann Nutr Metab. 2013;63:1180.

167. Winter FS, Emakam F, Kfutwah A, Hermann J, Azabji-Kenfack M, Krawinkel MB. The Effect of Arthrospira platensis Capsules on CD4 T-Cells and antioxidative capacity in a randomized pilot study of adult women infected with human immunodeficiency virus not under HAART in Yaoundé, Cameroon. Nutrients. 2014;6(7):2973-86.

168. Yamani E, Kaba-Mebri J, Mouala C, Gresenguet G, Rey JL. [Use of spirulina supplement for nutritional management of HIV-infected patients: study in Bangui, Central African Republic]. Medecine tropicale : Revue du Corps de Santé Colonial. 2009;69(1):66-70.

169. Young T. Effects of micronutrient supplementation on morbidity and mortality among HIV-infected individuals - A summary of the evidence. S Afr Med J. 2006;96(10):1062-4.

170. Zhao F, Feng XL, Xu W, Ma YM, Wang Z, Li WJ. [Effect of micronutrients on the immune status of human immunodeficiency virus-positive individuals]. Zhongguo Yi Xue Ke Xue Yuan Xue Bao. 2010;32(3):340-2.

171. Zulu RM, Byrne NM, Munthali GK, Chipeta J, Handema R, Musonda M, Hills AP. Assessing the impact of a food supplement on the nutritional status and body composition of HIV-infected Zambian women on ARVs. BMC Public Health. 2011;11(1):714.
