# Supplementary figures and images for: Effects of nutritional interventions on nutritional and immunological status and adherence to antiretroviral treatment among adults living with HIV in low- and middle-income countries: Systematic review and meta-analysis
Source: PLoS One. 2025 Jun 3;20(6):e0319843. doi: 10.1371/journal.pone.0319843 (PMC12132990; doi:10.1371/journal.pone.0319843)

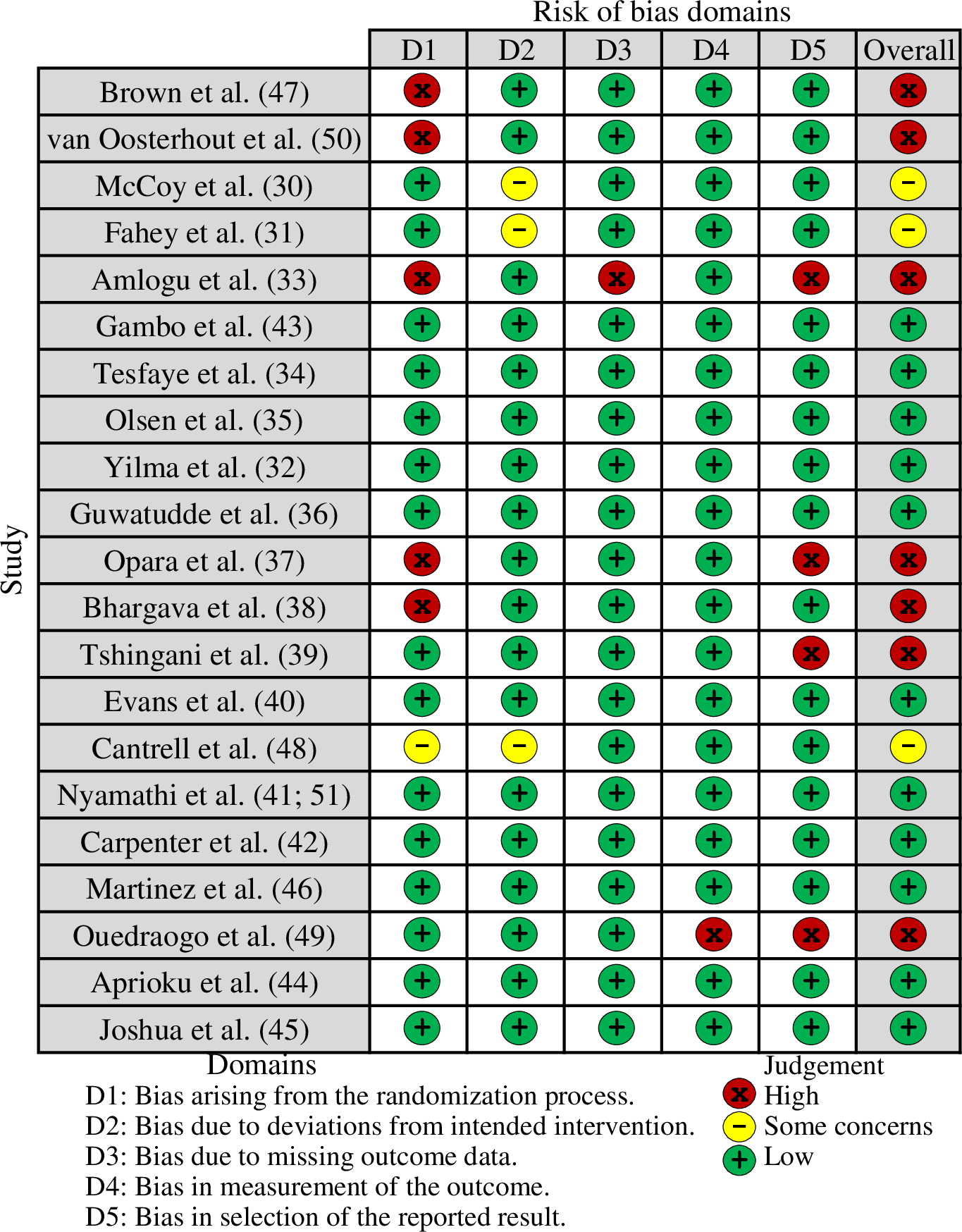

Supplement: S1 Fig — (TIF) [file pone.0319843.s008.tif]

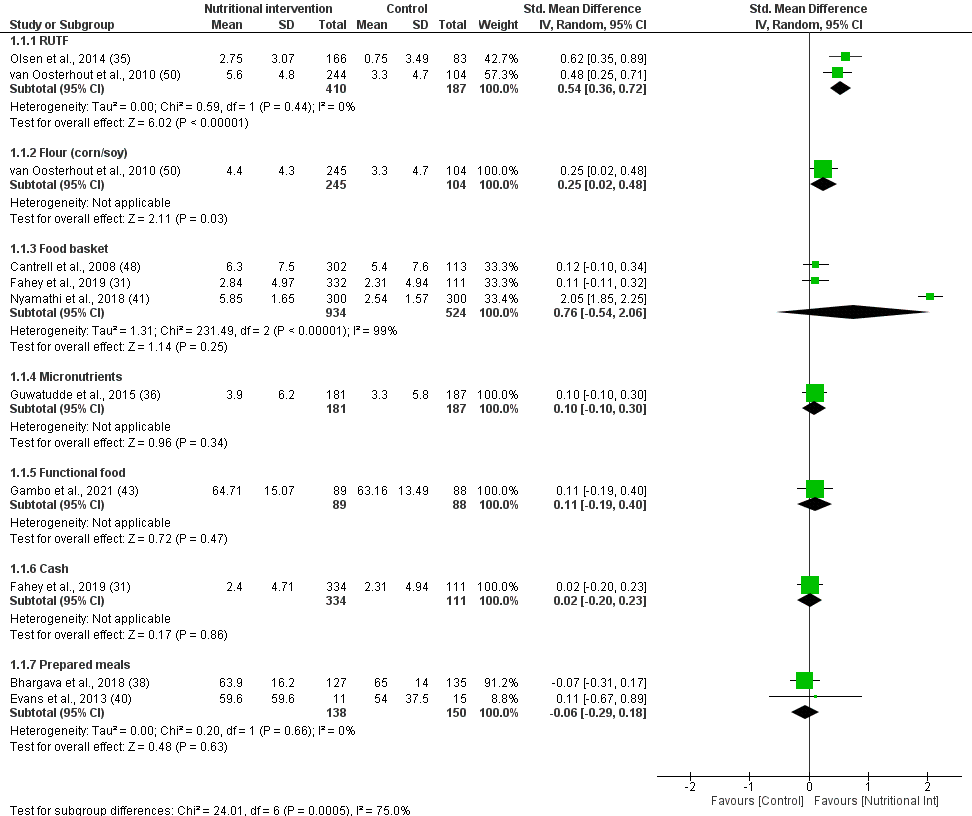

Supplement: S2 Fig — (TIF) [file pone.0319843.s009.tif]

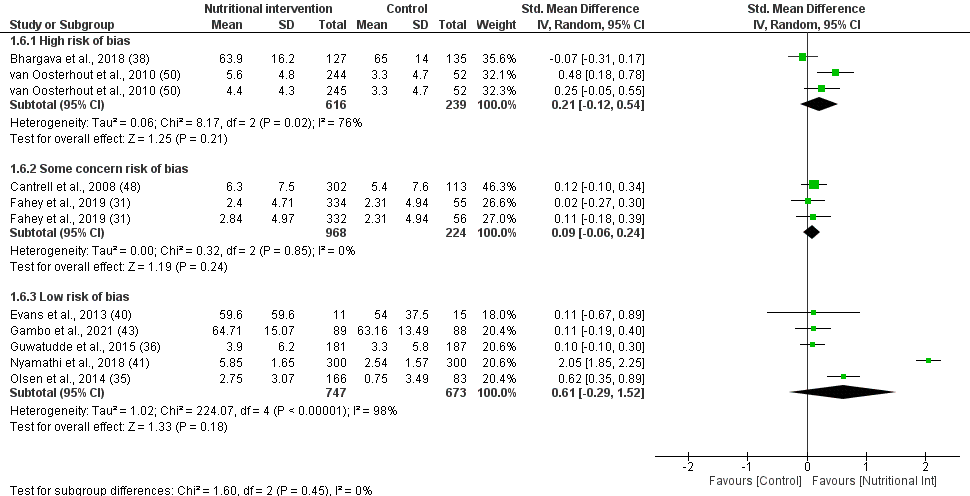

Supplement: S3 Fig — (TIF) [file pone.0319843.s010.tif]

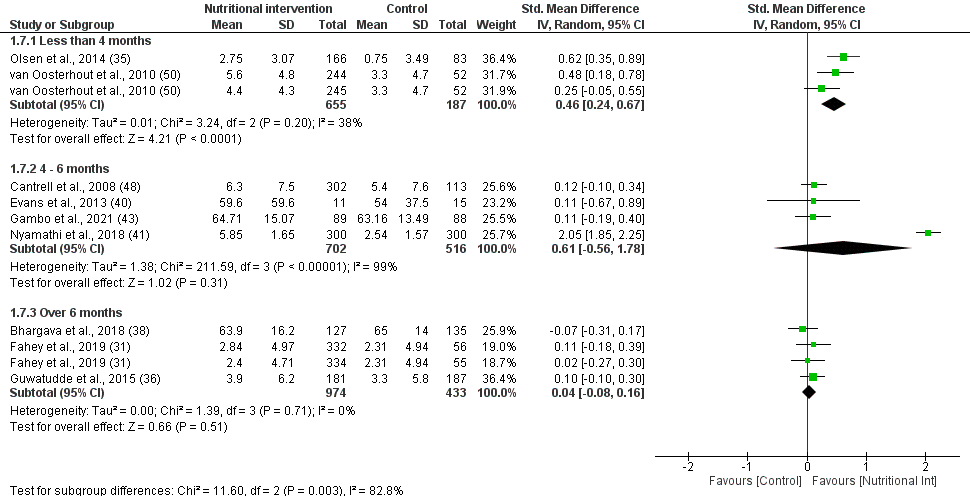

Supplement: S4 Fig — (TIF) [file pone.0319843.s011.tif]

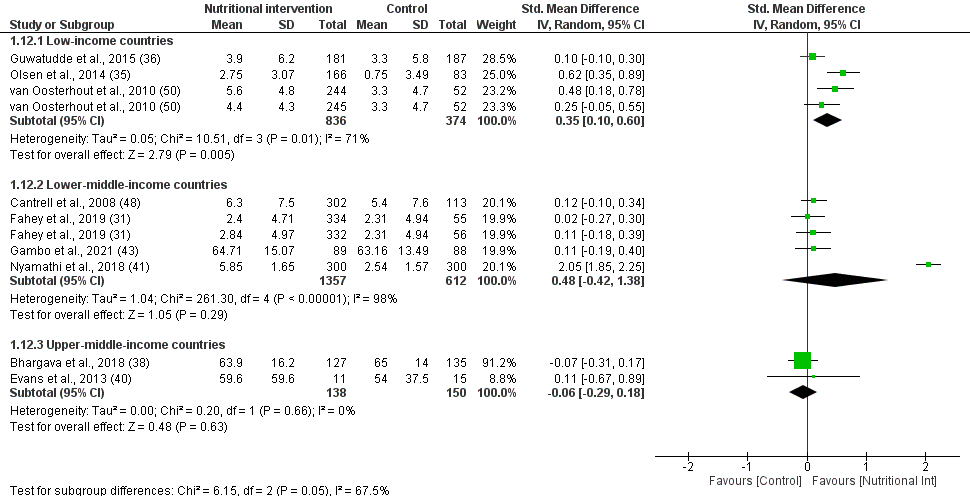

Supplement: S5 Fig — (TIF) [file pone.0319843.s012.tif]

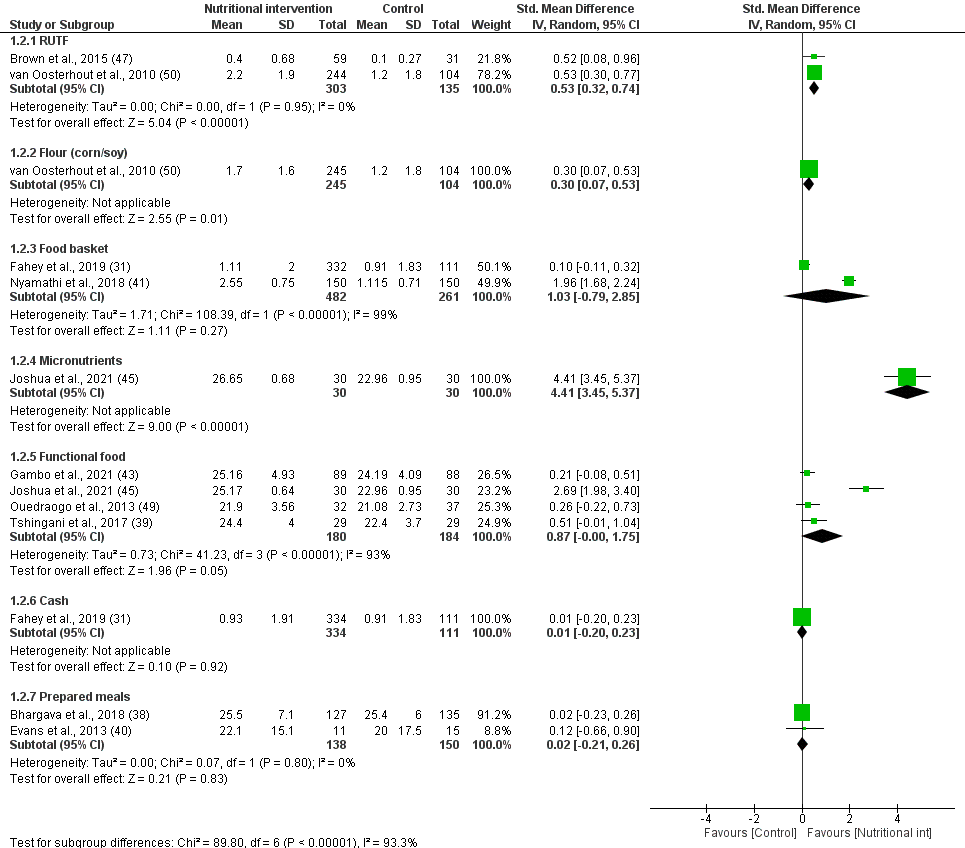

Supplement: S6 Fig — (TIF) [file pone.0319843.s013.tif]

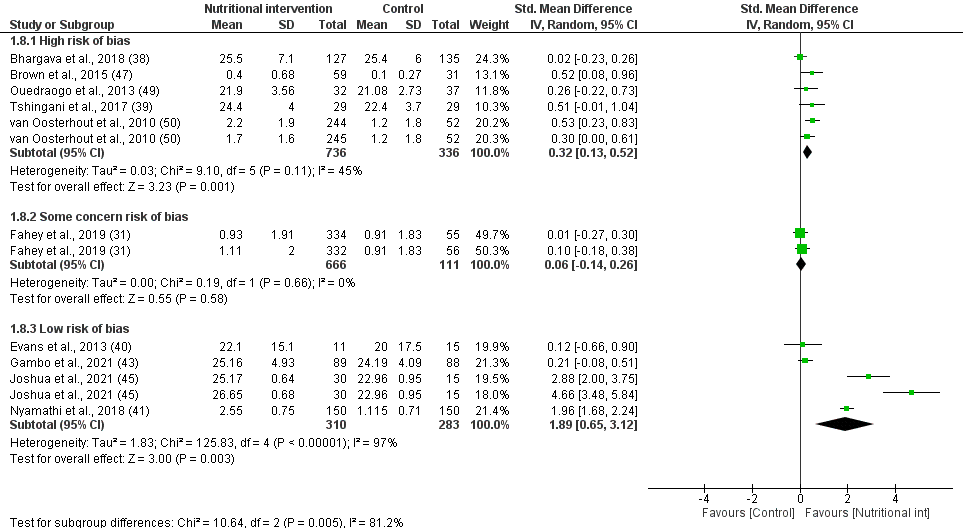

Supplement: S7 Fig — (TIF) [file pone.0319843.s014.tif]

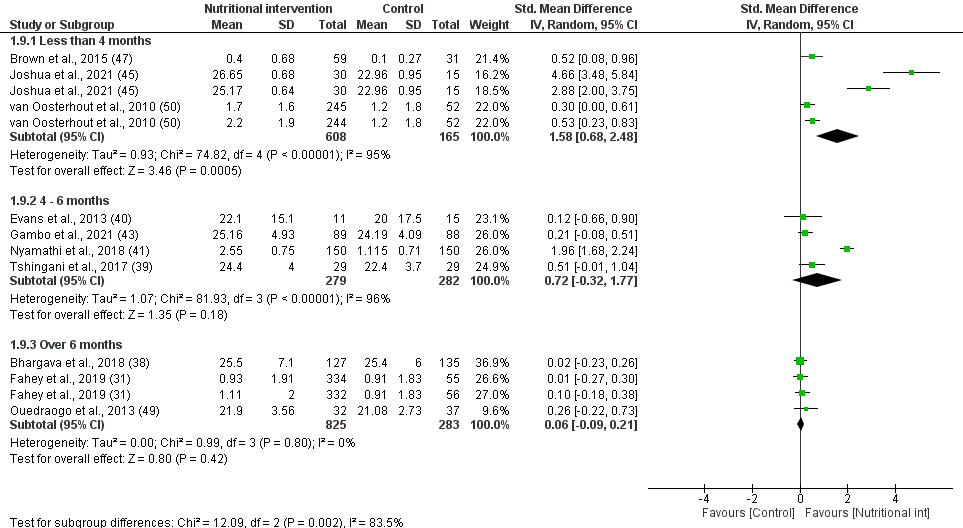

Supplement: S8 Fig — (TIF) [file pone.0319843.s015.tif]

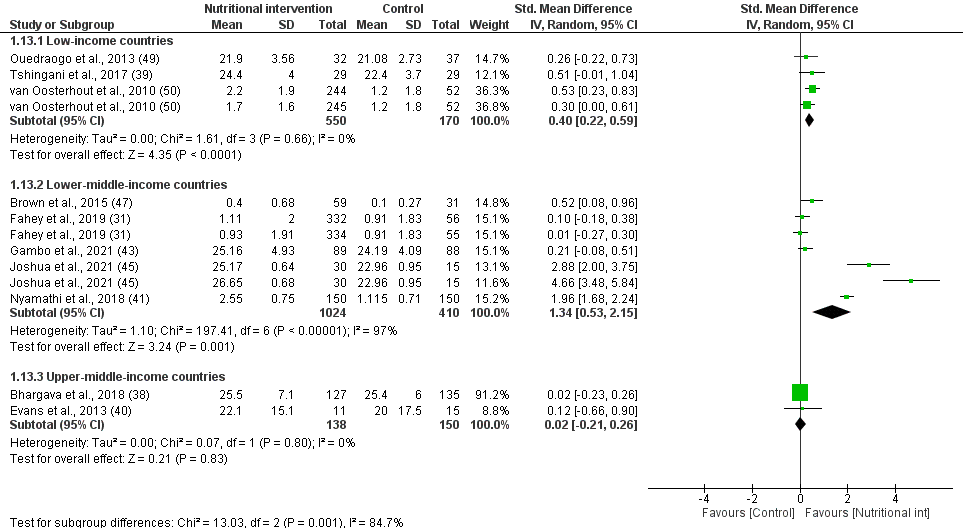

Supplement: S9 Fig — (TIF) [file pone.0319843.s016.tif]

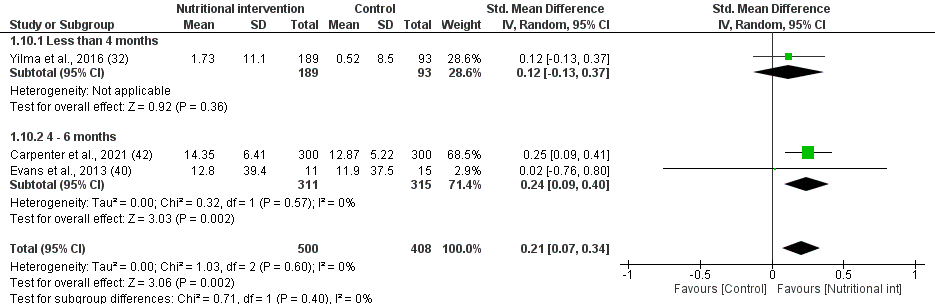

Supplement: S10 Fig — (TIF) [file pone.0319843.s017.tif]

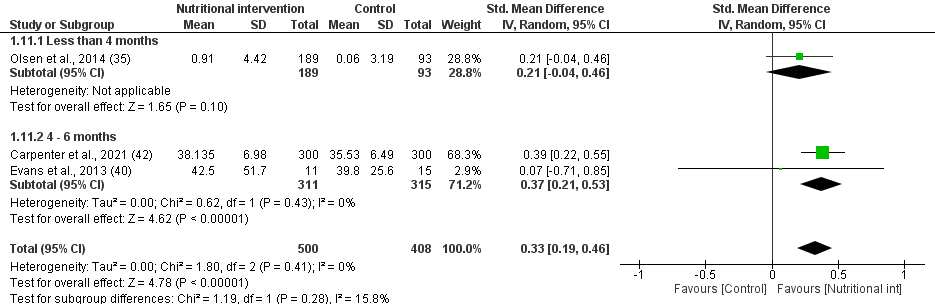

Supplement: S11 Fig — (TIF) [file pone.0319843.s018.tif]

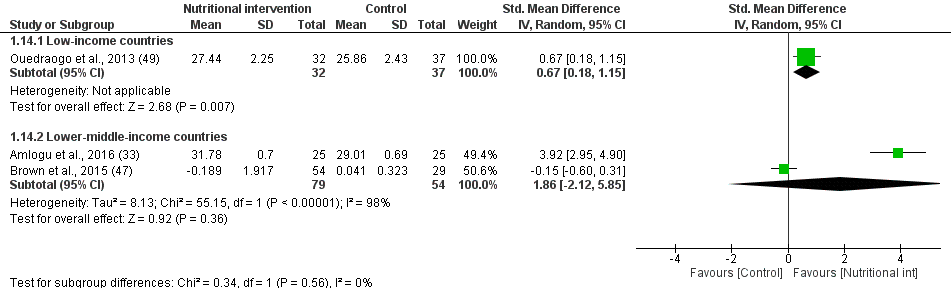

Supplement: S12 Fig — (TIF) [file pone.0319843.s019.tif]

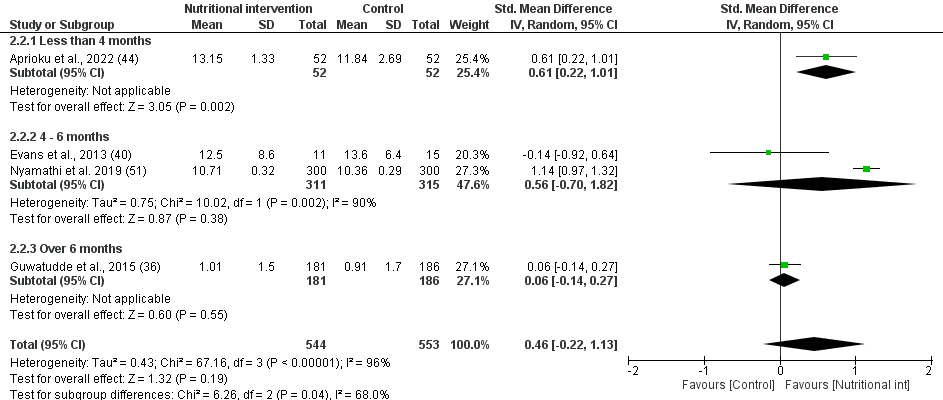

Supplement: S13 Fig — (TIF) [file pone.0319843.s020.tif]

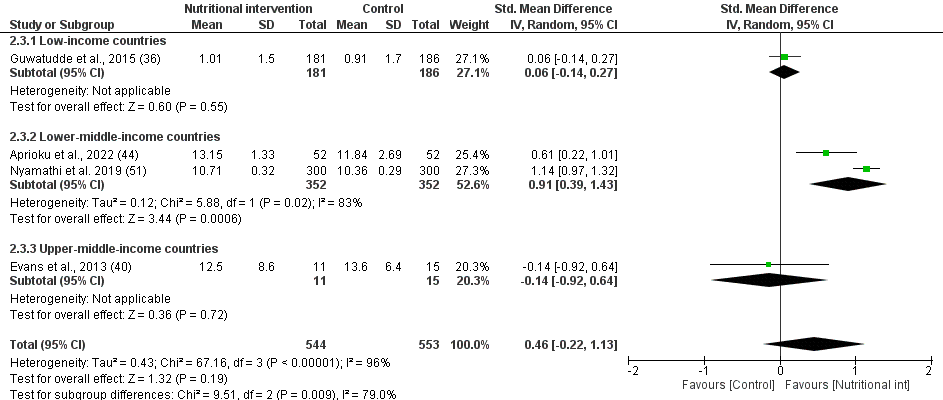

Supplement: S14 Fig — (TIF) [file pone.0319843.s021.tif]

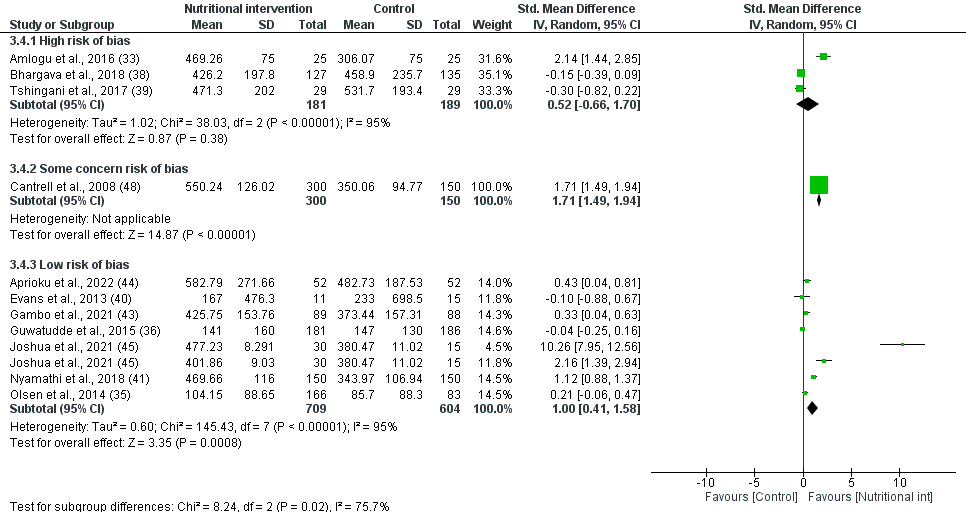

Supplement: S15 Fig — (TIF) [file pone.0319843.s022.tif]

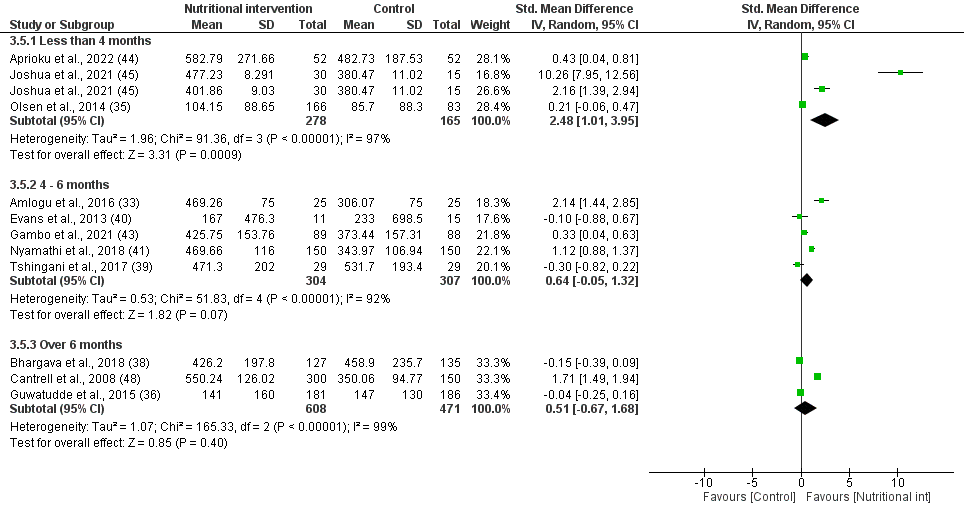

Supplement: S16 Fig — (TIF) [file pone.0319843.s023.tif]

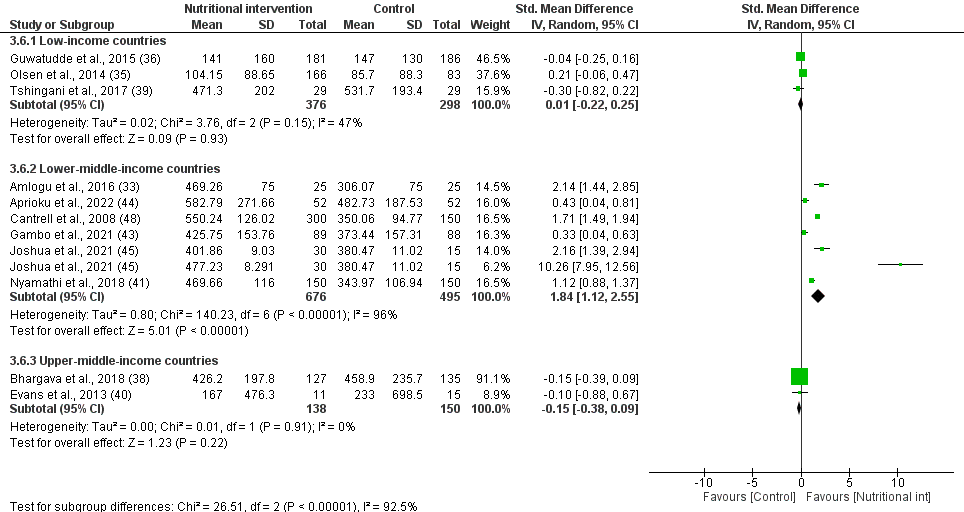

Supplement: S17 Fig — (TIF) [file pone.0319843.s024.tif]

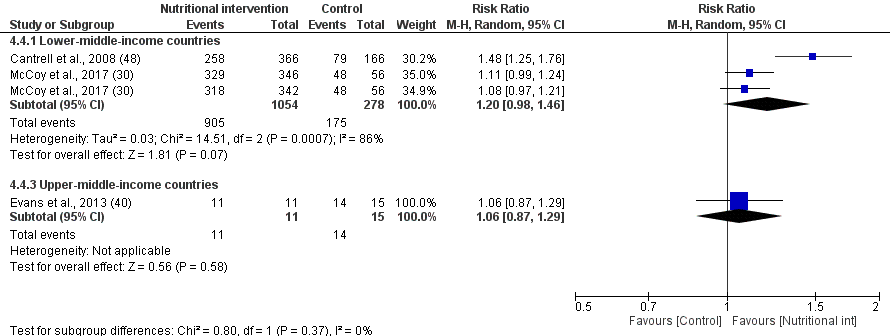

Supplement: S18 Fig — (TIF) [file pone.0319843.s025.tif]
