# Supplementary material for: Effects of nutritional interventions on nutritional and immunological status and adherence to antiretroviral treatment among adults living with HIV in low- and middle-income countries: Systematic review and meta-analysis
Source: PLoS One. 2025 Jun 3;20(6):e0319843. doi: 10.1371/journal.pone.0319843 (PMC12132990; doi:10.1371/journal.pone.0319843)
Supplement: Attachment — Submitted filename: PlosOne Response.docx [file pone.0319843.s027.docx]

PONE-D-24-28337

Effects of nutritional interventions on nutritional and immunological status and adherence to antiretroviral treatment among adults living with HIV in low- and middle-income countries: Systematic Review and Meta-Analysis

Dear Editor

# We thank the reviewers for their constructive comments, which helped us to improve our paper. Our responses to the reviewers are provided below, and all changes in the manuscript are marked with track changes. We hope that the revisions meet your expectations and that the manuscript will now be acceptable for publication.

Sincerely,

Alain Nahaskida

**Response to the editor:**

When submitting your revision, we need you to address these additional requirements.

1. Please ensure that your manuscript meets PLOS ONE's style requirements, including those for file naming. The PLOS ONE style templates can be found at

<https://journals.plos.org/plosone/s/file?id=wjVg/PLOSOne_formatting_sample_main_body.pdf> and

<https://journals.plos.org/plosone/s/file?id=ba62/PLOSOne_formatting_sample_title_authors_affiliations.pdf>

**Response**: The outline of the manuscript, including file naming is revised following the guideline.

2. We suggest you thoroughly copyedit your manuscript for language usage, spelling, and grammar. If you do not know anyone who can help you do this, you may wish to consider employing a professional scientific editing service.

The American Journal Experts (AJE) (<https://www.aje.com/>) is one such service that has extensive experience helping authors meet PLOS guidelines and can provide language editing, translation, manuscript formatting, and figure formatting to ensure your manuscript meets our submission guidelines. Please note that having the manuscript copyedited by AJE or any other editing services does not guarantee selection for peer review or acceptance for publication.

Upon resubmission, please provide the following:

The name of the colleague or the details of the professional service that edited your manuscript

A copy of your manuscript showing your changes by either highlighting them or using track changes (uploaded as a *supporting information* file)

A clean copy of the edited manuscript (uploaded as the new *manuscript* file)”.

**Response**: We have thoroughly revised the manuscript for language, spelling, and grammar by Scribendi <https://www.scribendi.com/>. Our submission incudes both revised and clean versions.

3. Thank you for stating the following financial disclosure:

[This work was conducted under the PhD studies of AN, whose scholarship was funded by the International Atomic Energy Agency (IAEA, Vienna)].

Please state what role the funders took in the study.  If the funders had no role, please state: ""The funders had no role in study design, data collection and analysis, decision to publish, or preparation of the manuscript.""

If this statement is not correct you must amend it as needed.

Please include this amended Role of Funder statement in your cover letter; we will change the online submission form on your behalf.

**Response**: The Role of the funder has been correctly added now to the system stating “The funders had no role in study design, data collection and analysis, decision to publish, or preparation of the manuscript.”

4. As required by our policy on Data Availability, please ensure your manuscript or supplementary information includes the following:

A numbered table of all studies identified in the literature search, including those that were excluded from the analyses.

For every excluded study, the table should list the reason(s) for exclusion.

If any of the included studies are unpublished, include a link (URL) to the primary source or detailed information about how the content can be accessed.

**Response:** The studies identified in the literature search were reported in two different tables. The excluded studies were reported in the Supplementary table 6, including the reference, title, and the reason for the exclusion. Table 1 summarizes the included studies.

A table of all data extracted from the primary research sources for the systematic review and/or meta-analysis. The table must include the following information for each study:

**Response:** Table 1 included all the characteristics of the studies included in the systematic review. Table 2 has been added to the results section, showing all the original extracted data.

Name of data extractors and date of data extraction

**Response:** Name of data extractors and date of data extraction are added to the text.

Confirmation that the study was eligible to be included in the review.

**Response:** A column indicating whether a study was eligible to be included in the meta-analysis or not was added to the table 1.

All data extracted from each study for the reported systematic review and/or meta-analysis that would be needed to replicate your analyses.

**Response:** All data extracted for the systematic review and meta-analysis are now shown in Table 2.

If data or supporting information were obtained from another source (e.g. correspondence with the author of the original research article), please provide the source of data and dates on which the data/information were obtained by your research group.

**Response:** Not applicable.

If applicable for your analysis, a table showing the completed risk of bias and quality/certainty assessments for each study or outcome. Please ensure this is provided for each domain or parameter assessed. For example, if you used the Cochrane risk-of-bias tool for randomized trials, provide answers to each of the signalling questions for each study. If you used GRADE to assess certainty of evidence, provide judgements about each of the quality of evidence factor. This should be provided for each outcome.

**Response:** Table 3 is added including the risk of bias of the studies by outcome.

An explanation of how missing data were handled.

**Response:** Not applicable.

This information can be included in the main text, supplementary information, or relevant data repository. Please note that providing these underlying data is a requirement for publication in this journal, and if these data are not provided your manuscript might be rejected.

**Reviewer #1:**

Thank you for reviewing the manuscript and providing valuable comments and suggestions. We will address all your comments in the following sections.

Line 138: what are controlled trials? aren't they already included under RCTs?

**Response**: Controlled trials emphasize the presence of a control group but do not specify the method of group assignment. While these studies include a control group for comparison, the lack of randomization increases the risk of bias and confounding variables, potentially affecting the validity of the findings. Controlled trials can include cohorts (one control and one intervention), quasi-experimental, historical control groups, and RCTs. RCT considers random allocation of the study participants, allowing a degree of rigor in the comparison.

Line 139: need to mention definition/criteria of LMIC used.

**Responded**: The definition of LMICs is based on the World Bank's classification of countries. A reference has now been included in the document.

Line 143: What about studies which included both children and adults and also reported subgroup data for adults?

**Response:** In our review, we focused on studies involving adult populations. However, we also included studies that encompassed both adults and children, provided that subgroup data for adults were available. For example, the study conducted in Vietnam by Brown et al. (2015) included participants across all age groups but reported results separately for adults and children, allowing us to include it in our analysis. In contrast, the study by Mallewa et al. (2018) [Effect of ready-to-use supplementary food on mortality in severely immunocompromised HIV-infected individuals in Africa initiating antiretroviral therapy (REALITY): an open-label, parallel-group, randomized controlled trial], which also involved both adults and children, presented combined outcomes for all age groups and was therefore excluded from our review. Additionally, we ensured that our criteria for study inclusion were clearly outlined in the methods section of the document.

For PRISMA, the 2020 PRISMA figure for databases + other sources should be used.

**Response:** We agree that the 2020 PRISMA figure is more appropriate. We have now used it as figure 1.

Reviewer #2: Dear Editor,

I appreciate the opportunity to review the manuscript, "Effects of nutritional interventions on nutritional and immunological status and adherence to antiretroviral treatment among adults living with HIV in low- and middle-income countries: systematic review and meta-analysis." In addition to contributing to the worldwide literature and raising intriguing ideas, the article aids in the achievement of Sustainable Development Goal (SDG) goal 3.3, which calls for the eradication of the HIV epidemic by 2030.Before endorsing for publication, I have included some of my thoughts and recommendations that may strengthen the article.

Thank you for your positive feedback. We will address your comments and recommendations in the following paragraphs.

**Abstract**

Although the abstracts were written well, the authors should have included a few of the meta-analysis models and techniques they employed for evaluating the outcome variable.

**Response**: We thank you for the reviewing the manuscript and for your valuable comments. We added the models of analysis in the abstract

The study's focus is on low- and middle-income countries, but it's also important to see how many of these countries are included in the abstract (or results) section.

**Response**: The studies were done in 12 countries (Malawi, Tanzania, Ethiopia, Nigeria, Uganda, South Africa, Demographic Republic of Congo, Zambia, and Burkina Faso, Vietnam, India and Honduras). We only added the number of countries to the abstract section because of the word limit in the abstract section. In the result section, we developed on this further. It reads now “The studies were conducted in 12 low- and middle-income countries (LMICs), including five low-income countries (Burkina Faso, Malawi, Ethiopia, the Democratic Republic of the Congo, and Uganda), six lower-middle-income countries (Tanzania, Nigeria, Zambia, Vietnam, India, and Honduras), and one upper-middle-income country (South Africa).”

**Introduction**

The introduction was well-written and presented; however, some of the lengthy statements needed to be supported by several citations (for e.g., lines 70-77).

**Response:** Thank you for drawing our attention to this important gap. We have supported all the statements with appropriate references including:

Reference 6: Bekker LG, Alleyne G, Baral S, Cepeda J, Daskalakis D, Dowdy D, et al. Advancing global health and strengthening the HIV response in the era of the Sustainable Development Goals: the International AIDS Society-Lancet Commission. Lancet. 2018;392(10144):312-58.

Reference 7: World Health Organization. "HIV/AIDS." Global Health Observatory (GHO) Data 2023 [January 10, 2025]. Available from: <https://www.who.int/data/gho/data/themes/hiv-aids>.

**Methods and Materials**

Why were pregnant and breastfeeding mothers or comorbidities excluded from the research population? Why were the authors unwilling to do sub-group analysis to determine the influence on policy? Were there any differences in the exclusion of those groups? The authors should have provided an explanation for their exclusion in the method section of their manuscript.

**Response**: They were mainly three categories of the research population that were not included in our review, that aimed to be more specific on adults, in food insecure settings. These were :1) pregnant and lactating women, 2) children, and 3) people living with HIV/AIDS who are suffering from co-morbidities. The main reason for their exclusion is the additional and specific needs that they might require in addition to ART.

We added this information to the method section that reads now “There were no restriction on the type or duration of the nutritional intervention, the language, or the year of publication. However, studies focusing exclusively on groups requiring additional nutritional care and non-ART medical treatment for co-morbidities were excluded. These include children under 18 years of age, pregnant or lactating women enrolled in the Prevention of Mother-to-Child Transmission program (for these programs include as well nutritional support), or individuals with comorbidities (e.g., tuberculosis). Studies that encompassed both adults and children, provided that subgroup data for adults were available, were included.”

**Result, discussion, and conclusion**

The results were well-written and narrated, and they discussed on the effect of nutritional interventions on PLWHA adherence to ART, food security, weight, BMI, body composition (including fat and fat-free mass), and MUAC. What about the findings that were made about particular countries? analysis of subgroups by gender or by country? Which countries or gender require more focus? What about gender-specific or country-specific policy implications? Are low-middle-income countries affected similarly by the findings? No environmental or geographic impact? or differences within health institutions? how the authors came up with those differences as their subject matter pertaining to LMICs.

**Response:** While we acknowledge the importance of gender- and country-specific factors in understanding the relevance, uptake, and scalability of these results, our review serves as preliminary work to highlight the critical role of nutrition in programs targeting the treatment of HIV/AIDS. The heterogeneity in interventions, settings, and population characteristics posed significant challenges, making further subgroup analyses less robust.

Our initial hypothesis—that food insecurity and malnutrition negatively impact the uptake of health services, particularly those aimed at preventing and eliminating HIV/AIDS—remains central and warrants continued emphasis. Unfortunately, gender-based analyses were not feasible in most cases due to the lack of separately reported results, except for limited instances in regression analyses.

That is said, we fully agree with your observation on the importance of these factors when targeting specific groups. As such, we have conducted additional sub-group analyses based on the income status of the country, when possible. We also included these considerations in the limitations section of the review.

The recommendation is not feasible; who can benefit? low or middle-income countries?

**Response**: Our study had limitations that did not allow us to make firm recommendations, but even these few nutritional and immunological indicators could be useful for low- and middle-income countries. Although we did conduct additional analyses based on the income status of the country, the results varied depending on the outcome. We have now re-wrote the conclusion statement and we hope that this answers your concern.
